# Supplementary material for: Multiple-testing corrections in selection scans using identity-by-descent segments
Source: Am J Hum Genet. 2025 Sep 26;112(11):2751–71. doi: 10.1016/j.ajhg.2025.09.004 (PMC12668784; doi:10.1016/j.ajhg.2025.09.004)
Supplement: Document S1. Figures S1–S19, Tables S1–S3, and supplemental acknowledgments [file mmc1.pdf]

The American Journal of Human Genetics, Volume *112*

Supplemental information

Multiple-testing corrections in selection scans using identity-by-descent segments

Seth D. Temple and Sharon R. Browning

Supplemental figures

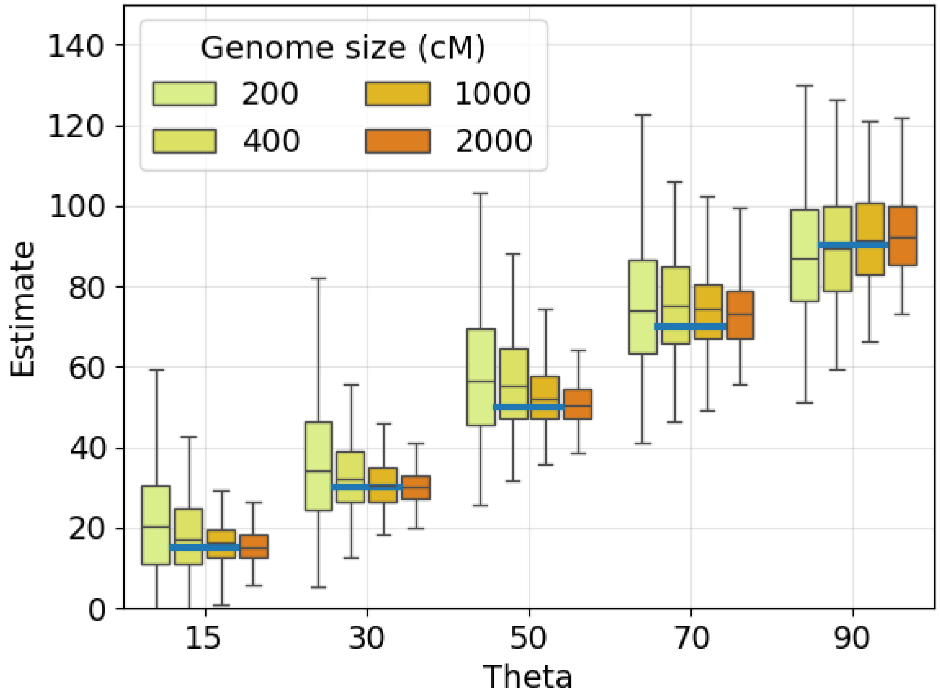

Figure S1: Estimating the exponential decay parameter θ from simulated Ornstein-Uhlenbeck processes. The 1st, 25th, 50th, 75th, and 99th percentiles of estimates $\hat{\theta}$ (y-axis) are shown for true θ (x-axis and horizontal blue lines). We estimate θ with different genome lengths (colors in legend) and step size 0.02 cM. Percentiles are taken over five hundred simulations for each θ.

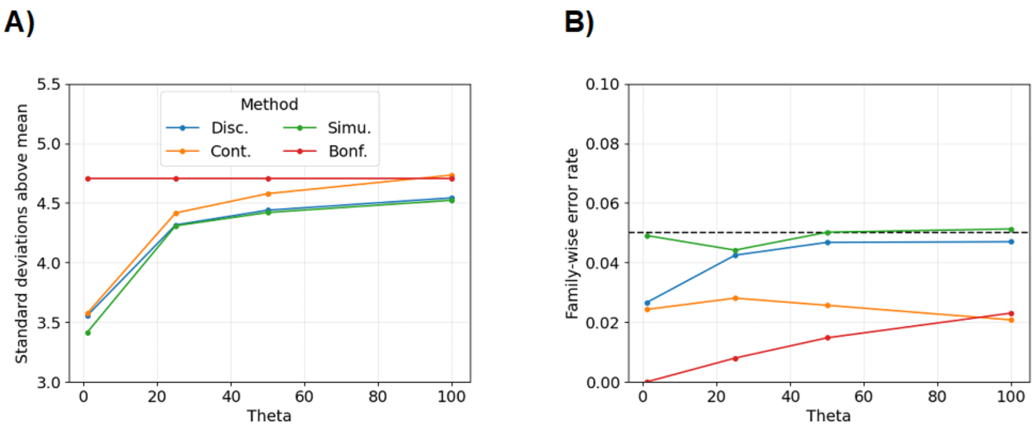

Figure S2: Multiple-testing approaches in simulations of Ornstein-Uhlenbeck processes. Line plots show A) standard deviations above the mean thresholds or B) family-wise error rates (y-axis) with different θ (x-axis). The multiple-testing approaches are (blue) the discrete-spacing analytical approximation, (orange) the continuous-spacing analytical approximation, (green) the simulation-based approach, and (red) the Bonferroni correction. The simulation-based approach is based on ten thousand simulations. The step size is hypothesis testing every 0.05 cM (50 kb). The data for each simulation is equivalent to twenty chromosomes, each of length 100 cM.

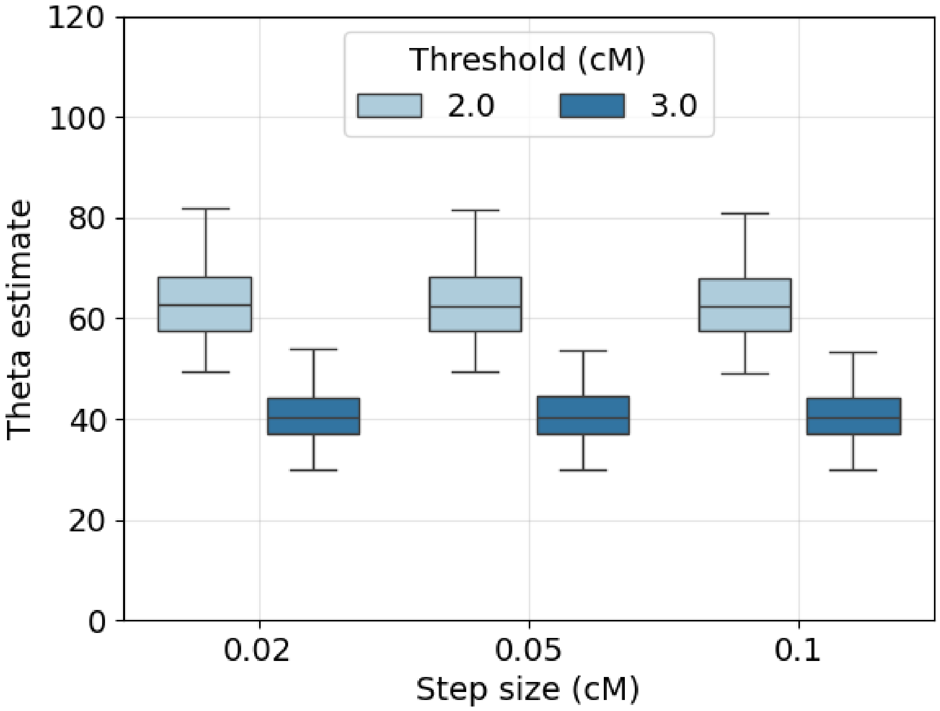

Figure S3: Estimating the exponential decay parameter θ from simulated IBD rate processes with different cM length thresholds. Box plots show the 1st, 25th, 50th, 75th, and 99th percentiles of estimates $\hat{\theta}$ using the IBD rate processes with simulated true IBD segments (dark blue) ≥ 2.0 cM and (light blue) ≥ 3.0 cM from `tskibd`. Estimates $\hat{\theta}$ are based on autocovariances calculated at different step sizes (x-axis). There are fifteen hundred simulations for each step size. The demographic model is the population bottleneck. The data for each simulation is equivalent to ten chromosomes of uniform length 100 cM.

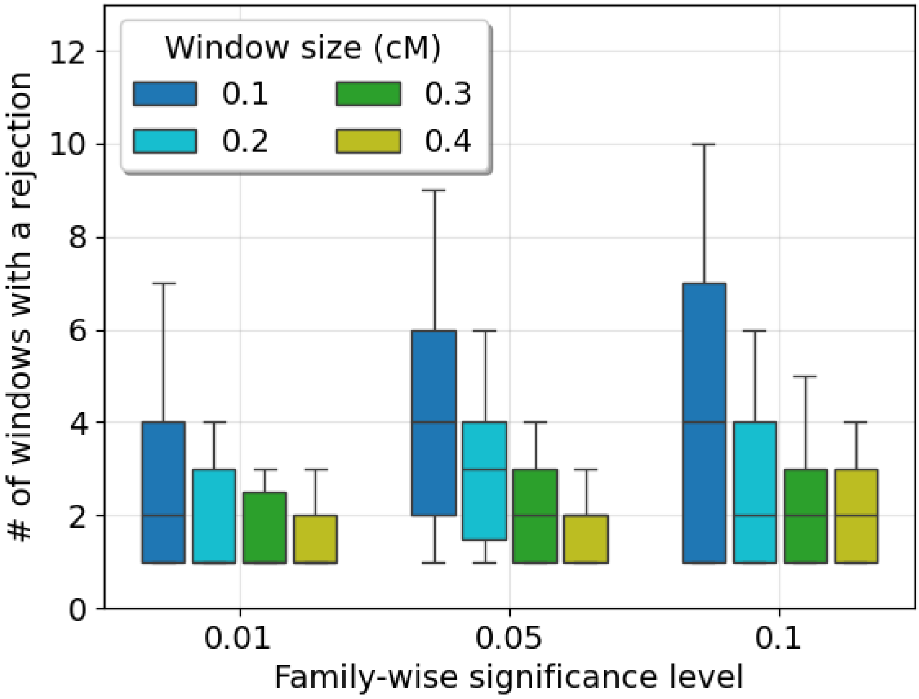

Figure S4: The number of windows with a rejected hypothesis test. Box plots show the 10th, 25th, 50th, 75th, and 90th percentiles of the number of non-overlapping windows with at least one rejection of the null hypothesis (y-axis). Windows sizes are 0.1, 0.2, 0.3, and 0.4 cM (colors in legend) with IBD rates calculated every 0.02 cM. Simulations in which there are no genome-wide significant tests are not included in the box plots. The multiple-testing method is the discrete-spacing analytical approximation using true IBD segments $\geq$ 2.0 cM. There are five hundred simulations for each family-wise significance level (x-axis). The demographic model is the population bottleneck. The data for each simulation is equivalent to ten chromosomes of uniform length 100 cM.

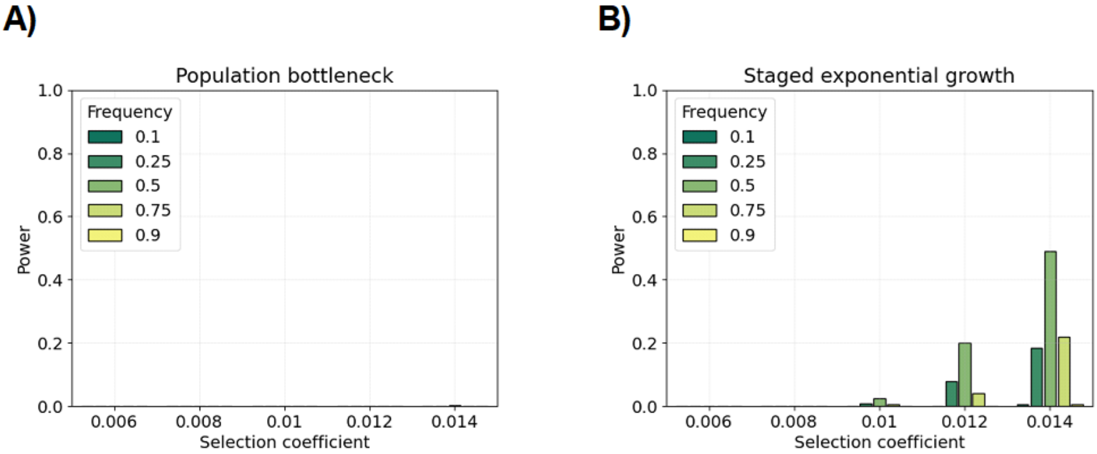

Figure S5: Power simulations for the ≥ 3.0 cM scan in different demographic models. Bar plots show the statistical power (y-axis) in the A) population bottleneck and B) staged exponential growth models using true IBD segments ≥ 3.0 cM overlapping the selected allele. Power is the proportion of tests where the null model is rejected at the p value threshold corresponding to the 0.05 family-wise significance level. Hypothesis testing is based on the discrete-spacing analytical threshold using the step size 0.02 cM. There are two hundred simulations for each pair of selection coefficient (x-axis) and current-day allele frequency (colors in legend).

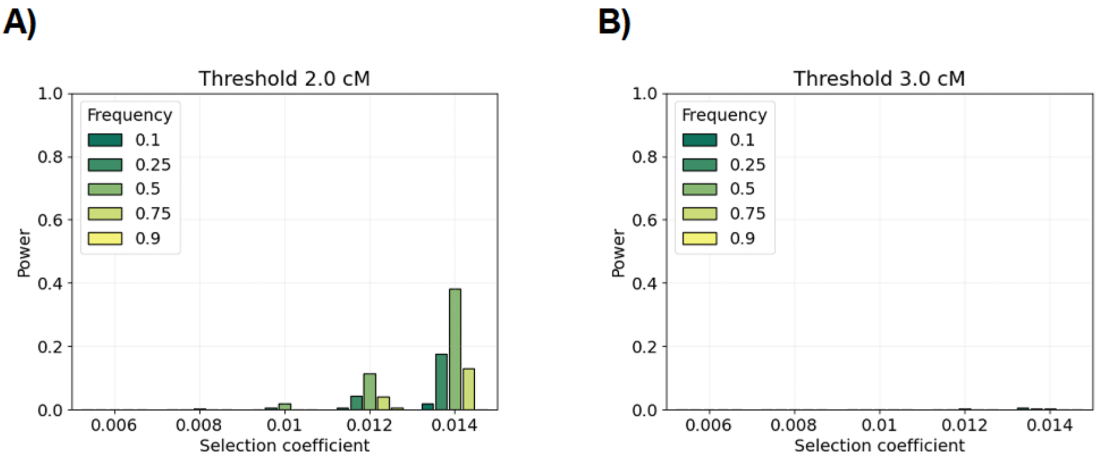

Figure S6: Power simulations in a constant size population. Bar plots show the statistical power (y-axis) using true IBD segments A) $\geq$ 2.0 cM or B) $\geq$ 3.0 cM overlapping the selected allele. Power is the proportion of tests where the null model is rejected at the p value threshold corresponding to the 0.05 family-wise significance level. Hypothesis testing is based on the discrete-spacing analytical threshold using the step size 0.02 cM. There are two hundred simulations for each pair of selection coefficient (x-axis) and current-day allele frequency (colors in legend).

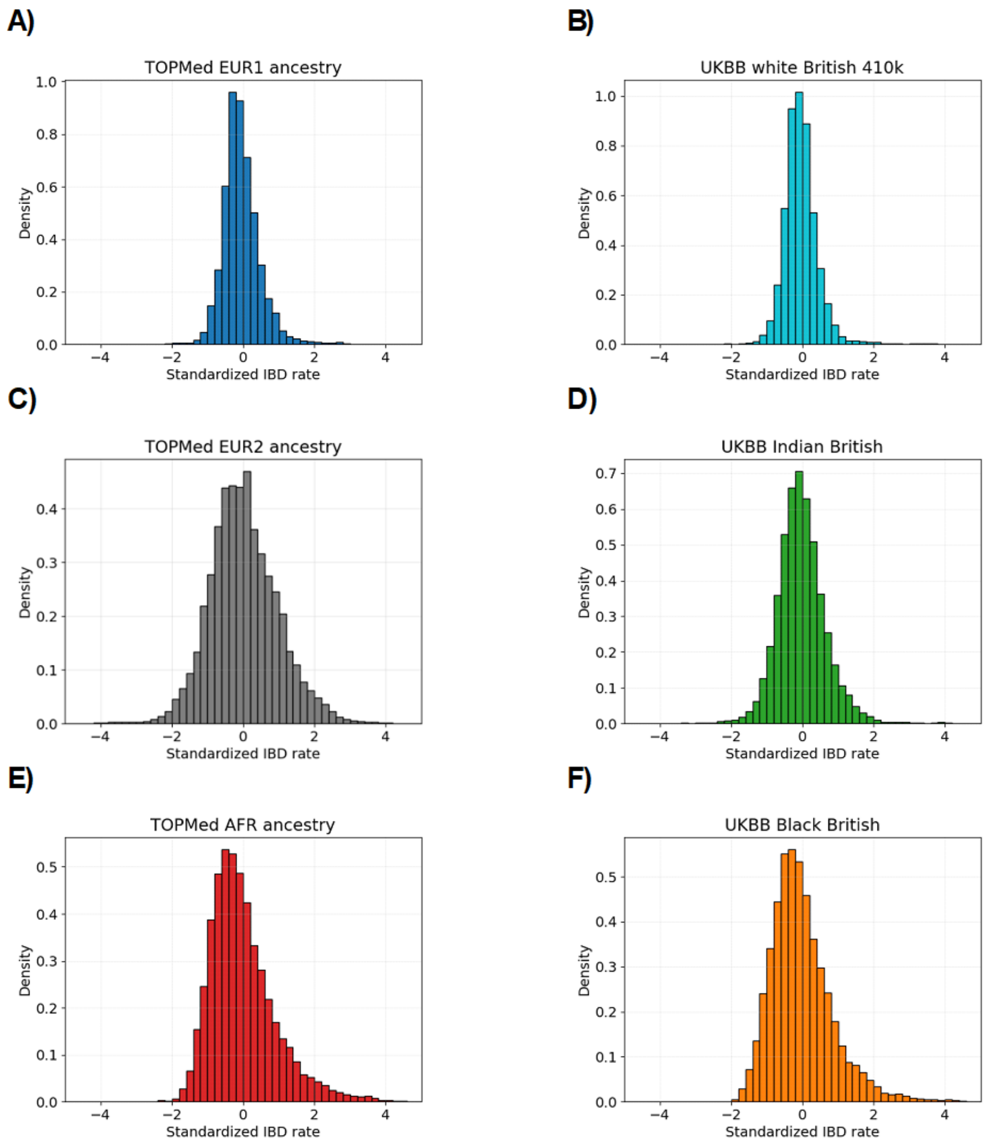

Figure S7: Histograms of IBD rates in human populations. The standardized IBD rates ≥ 2.0 cM (x-axis) are shown for A) TOPMed EUR1, B) UKBB white British, C) TOPMed EUR2, D) UKBB Indian British, E) TOPMed AFR ancestry, and F) UKBB Black British sample sets. Each histogram has fifty bins, and the x-axes range from -5 to 5.

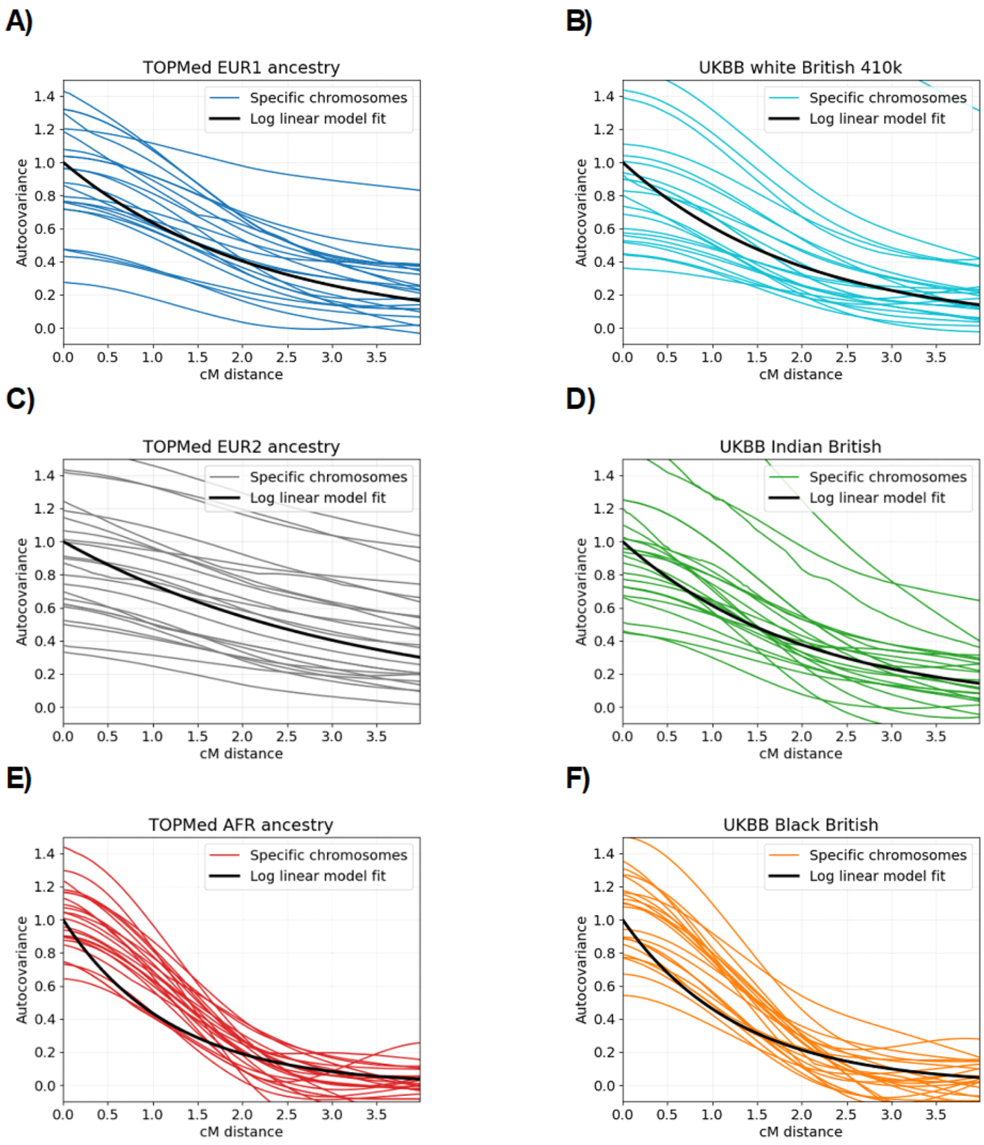

Figure S8: Estimating exponential decay parameter θ in real data. Each faint colored line shows estimated autocovariances (y-axis) for different cM distances (x-axis) and a specific chromosome. The black lines show the predicted autocovariances from the fitted Ornstein-Uhlenbeck processes using estimates $\hat{\theta}$. The data for each subplot is based on A) TOPMed EUR1 ancestry, B) UKBB white British, C) TOPMed EUR2 ancestry, D) UKBB Indian British, E) TOPMed AFR ancestry, and F) UKBB Black British sample sets.

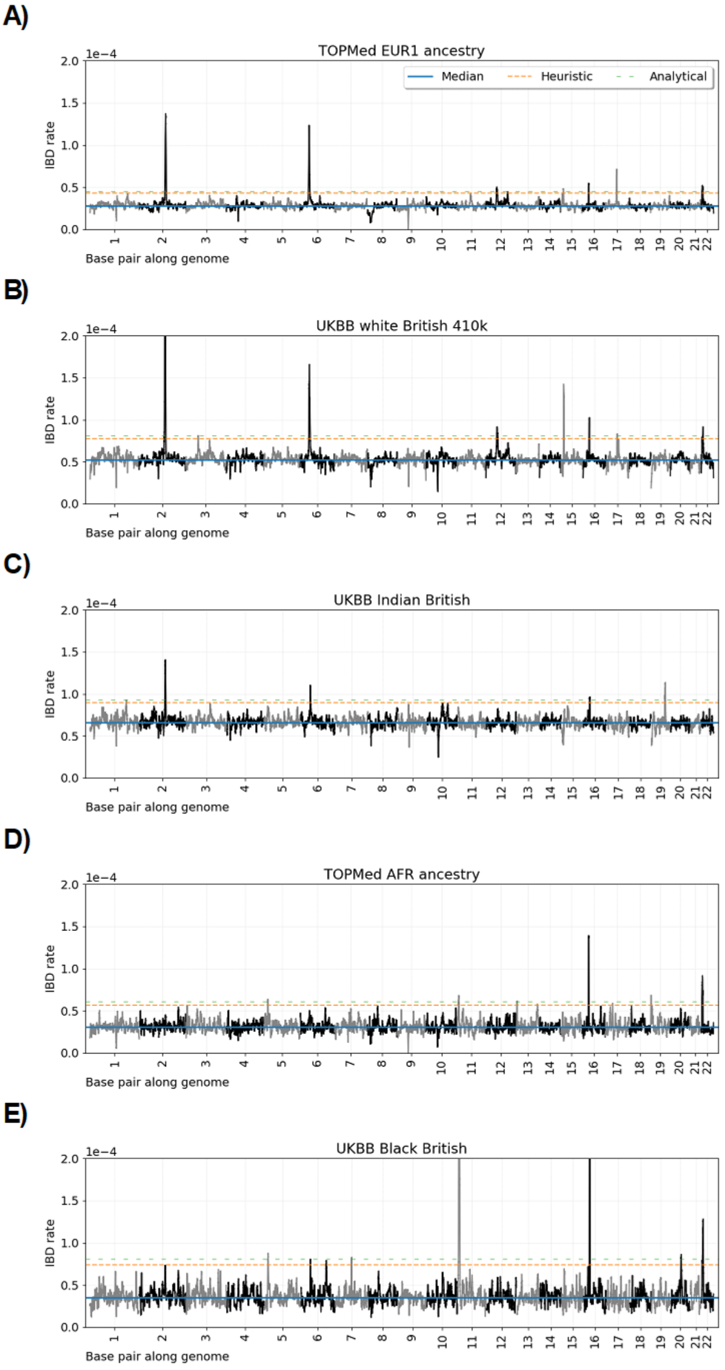

Figure S9: Genome-wide IBD rate scans using the ≥ 3.0 cM threshold. Line plots show IBD rates every 0.02 cM (y-axis) for base pair positions along twenty-two human autosomes. The data for each subplot is based on A) TOPMed EUR1 ancestry, B) UKBB white British, C) UKBB Indian British, D) TOPMed AFR ancestry, and E) UKBB Black British sample sets. Horizontal dashed lines show (blue) the genome-wide median IBD rate, (orange) the heuristic threshold of four standard deviations above the median, and (green) the analytical multiple-testing threshold.

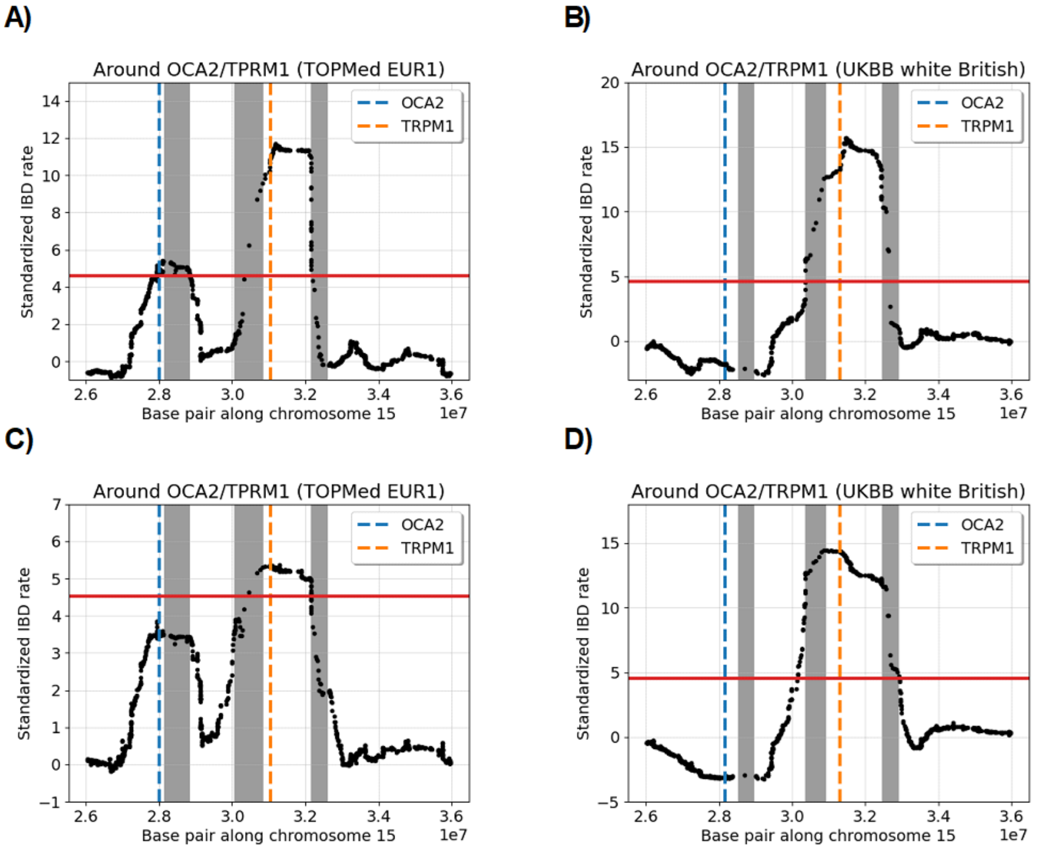

Figure S10: IBD rates around pigmentation genes in European ancestry samples. Scatter plots show the standardized IBD rates by physical base pair (chromosome 15) for A,C) TOPMed EUR1 and B,D) UKBB white British samples. The IBD segment detection thresholds are A-B) 2.0 cM and C-D) 3.0 cM. The horizontal red lines mark the genome-wide significance thresholds. The vertical blue and orange dashed lines mark the approximate midpoints of *OCA2* and *TRPM1*. The y-axis scales may differ to accentuate the dataset-specific signal. The physical base pairs are with respect to different assemblies. Problematic regions are shaded in gray.

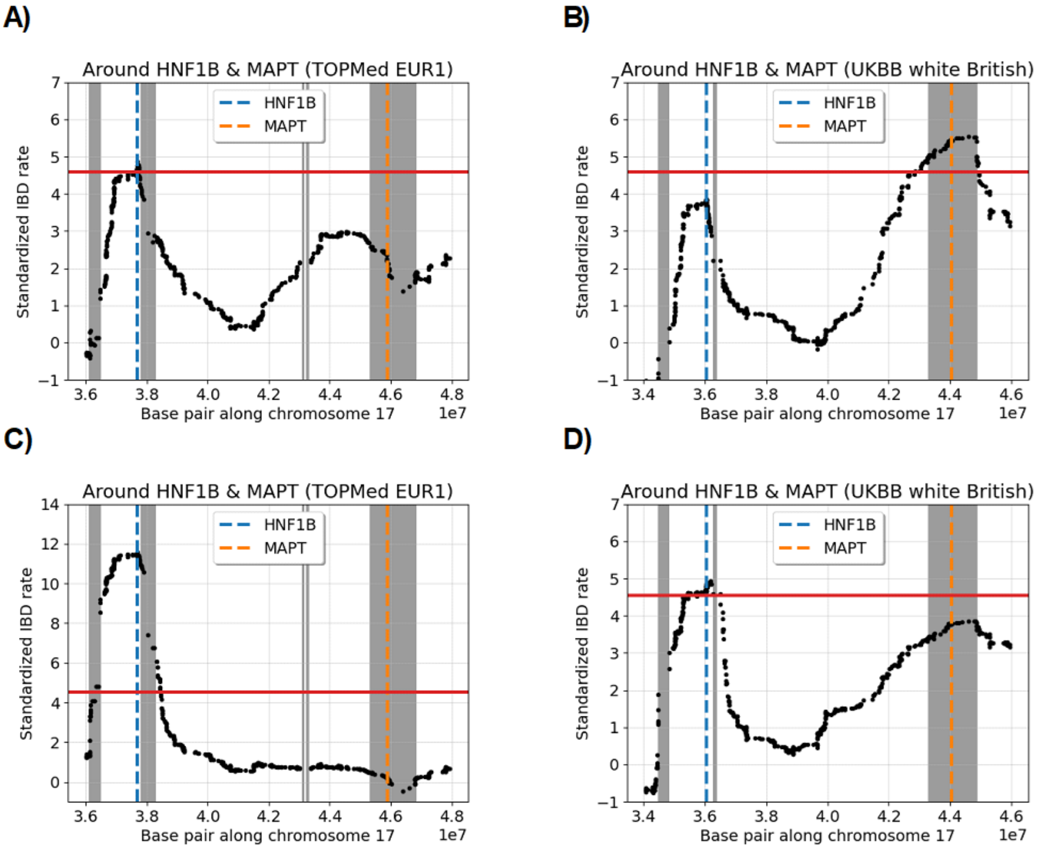

Figure S11: IBD rates around *HNF1B* and *MAPT* in European ancestry samples. Scatter plots show the standardized IBD rates by physical base pair (chromosome 17) for A,C) TOPMed EUR1, and B,D) UKBB white British samples. The IBD segment detection thresholds are A-B) 2.0 cM and C-D) 3.0 cM. The horizontal red lines mark the genome-wide significance thresholds. The vertical blue and orange dashed lines mark the approximate midpoints of *HNF1B* and *MAPT*. The y-axis scales may differ to accentuate the dataset-specific signal. The physical base pairs are with respect to different assemblies. Problematic regions are shaded in gray.

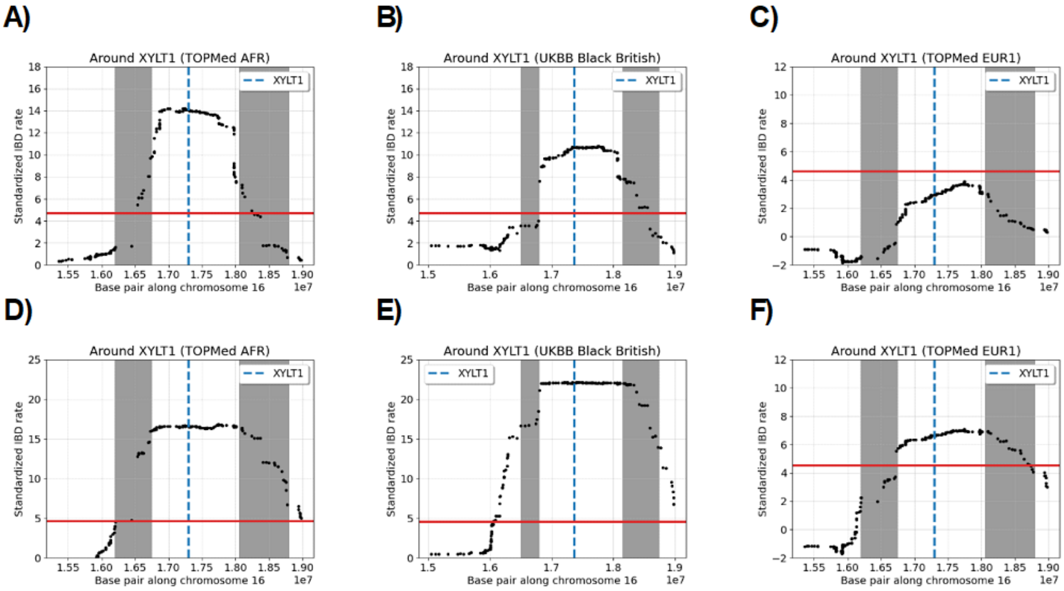

Figure S12: IBD rates around *XYLT1* in African and European ancestry samples. Scatter plots show the standardized IBD rates by physical base pair (chromosome 16) for A,D) TOPMed AFR, B,E) UKBB Black British, and C,F) TOPMed EUR1 samples. The IBD segment detection thresholds are A-C) 2.0 cM and D-F) 3.0 cM. The horizontal red lines mark the genome-wide significance thresholds. The vertical blue dashed lines mark the approximate midpoint of *XYLT1*. The y-axis scales may differ to accentuate the dataset-specific signal. The physical base pairs are with respect to different assemblies. Problematic regions are shaded in gray.

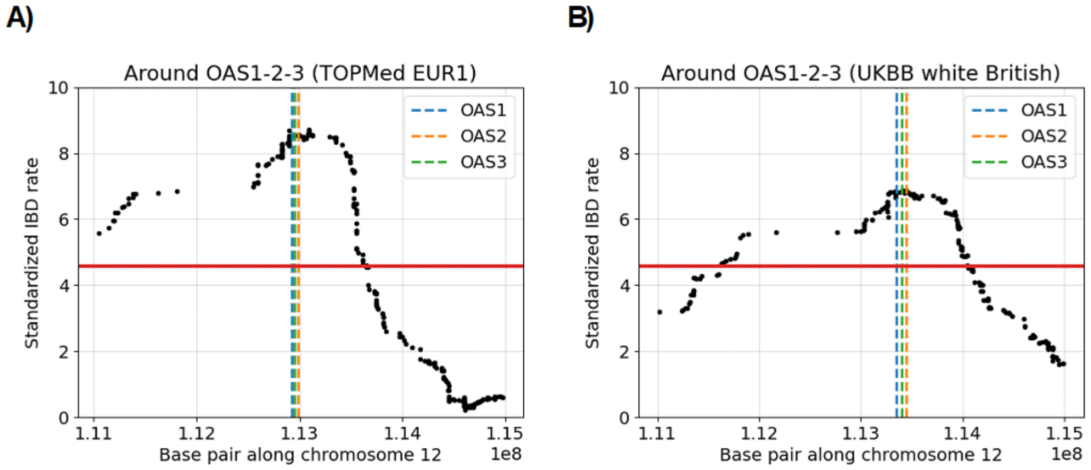

Figure S13: IBD rates around *OAS1-2-3* in European ancestry samples. Scatter plots show the standardized IBD rates by physical base pair (chromosome 12) for A) TOPMed EUR1 and B) UKBB white British samples. The IBD segment detection thresholds is 2.0 cM. The horizontal red lines mark the genome-wide significance thresholds. The vertical blue, orange, and green dashed lines mark the approximate midpoints of *OAS1-2-3*. The physical base pairs are with respect to different assemblies. There are no large problematic regions around this locus.

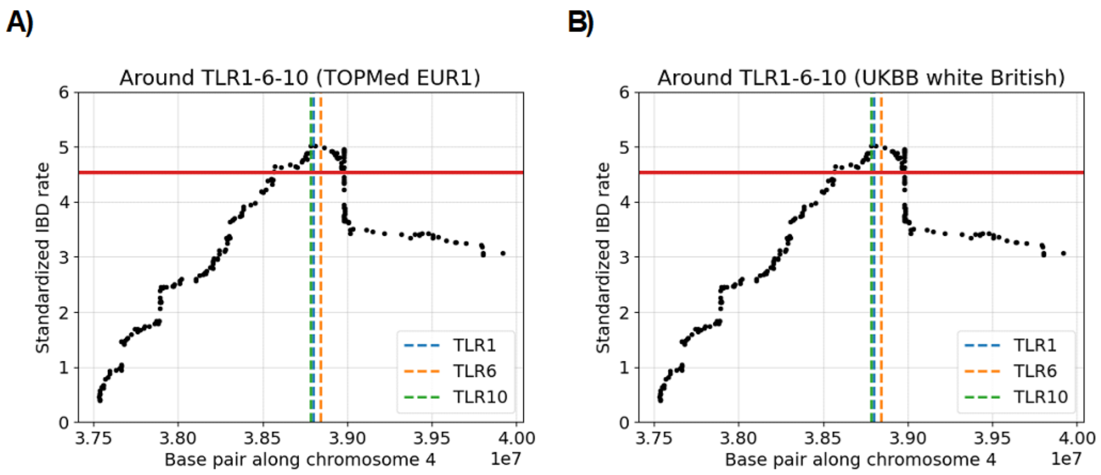

Figure S14: IBD rates around toll-like receptor genes in European ancestry samples. Scatter plots show the standardized IBD rates by physical base pair (chromosome 4) for A) TOPMed EUR1 and B) UKBB white British samples. The IBD segment detection threshold is 2.0 cM. The horizontal red lines mark the genome-wide significance thresholds. The vertical blue, orange, and green dashed lines mark the approximate midpoints of *TLR1-6-10*. The physical base pairs are with respect to different assemblies. There are no large problematic regions around this locus.

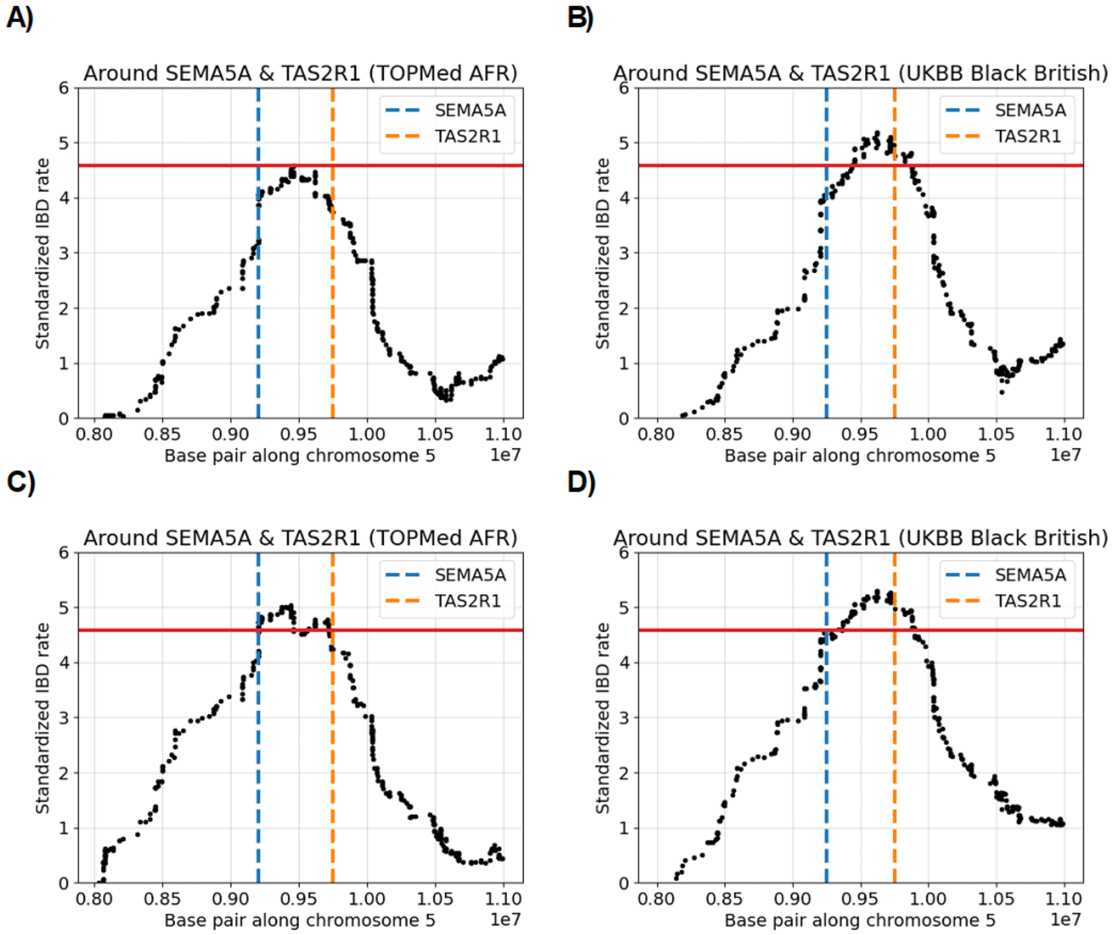

Figure S15: IBD rates around *SEMA5A* and *TAS2R1* in African ancestry samples. Scatter plots show the standardized IBD rates by physical base pair (chromosome 5) for A,C) TOPMed AFR, and B,D) UKBB Black British samples. The IBD segment detection thresholds are A-B) 2.0 cM and C-D) 3.0 cM. The horizontal red lines mark the genome-wide significance thresholds. The vertical blue and orange dashed lines mark the approximate midpoints of *SEMA5A* and *TAS2R1*. The physical base pairs are with respect to different assemblies. There are no large problematic regions around this locus.

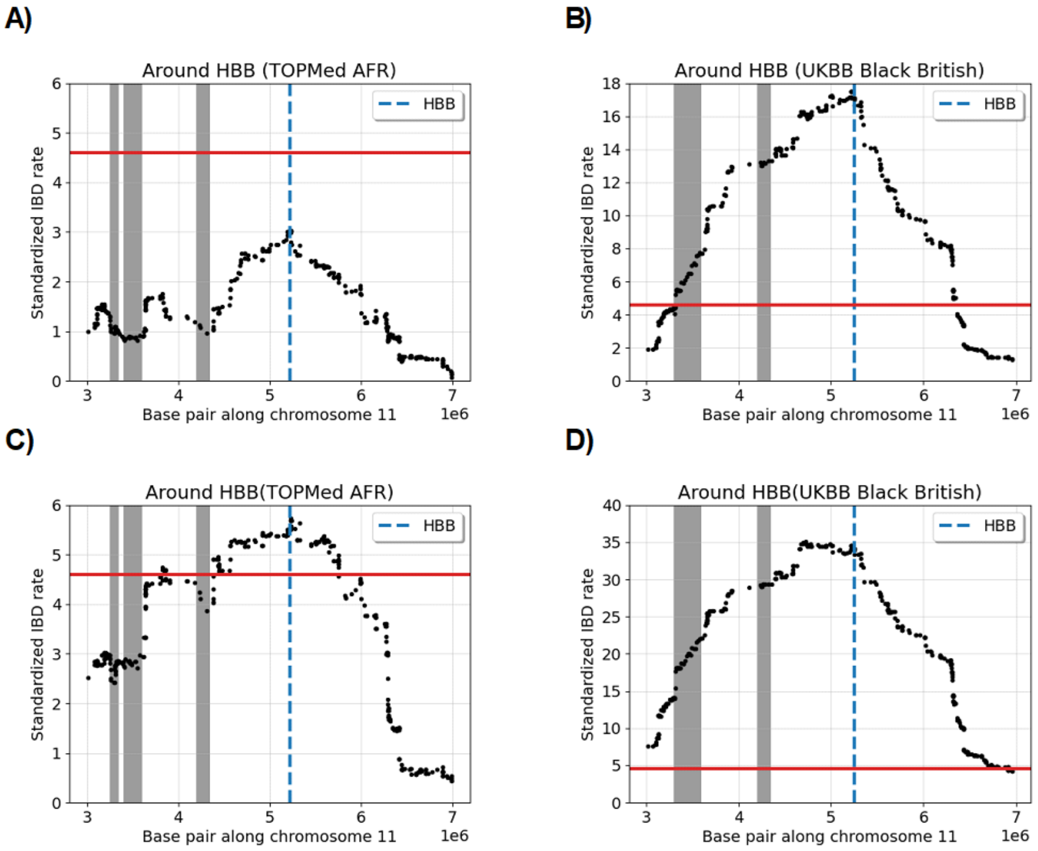

Figure S16: IBD rates around hemoglobin beta genes of African ancestry samples. Scatter plots show the standardized IBD rates by physical base pair (chromosome 11) for A,C) TOPMed AFR and B,D) UKBB Black British samples. The IBD segment detection thresholds are A-B) 2.0 cM and C-D) 3.0 cM. The horizontal red lines mark the genome-wide significance thresholds. The vertical blue dashed lines mark the approximate midpoint of *HBB*. The y-axis scales may differ to accentuate the dataset-specific signal. The physical base pairs are with respect to different assemblies. Problematic regions are shaded in gray.

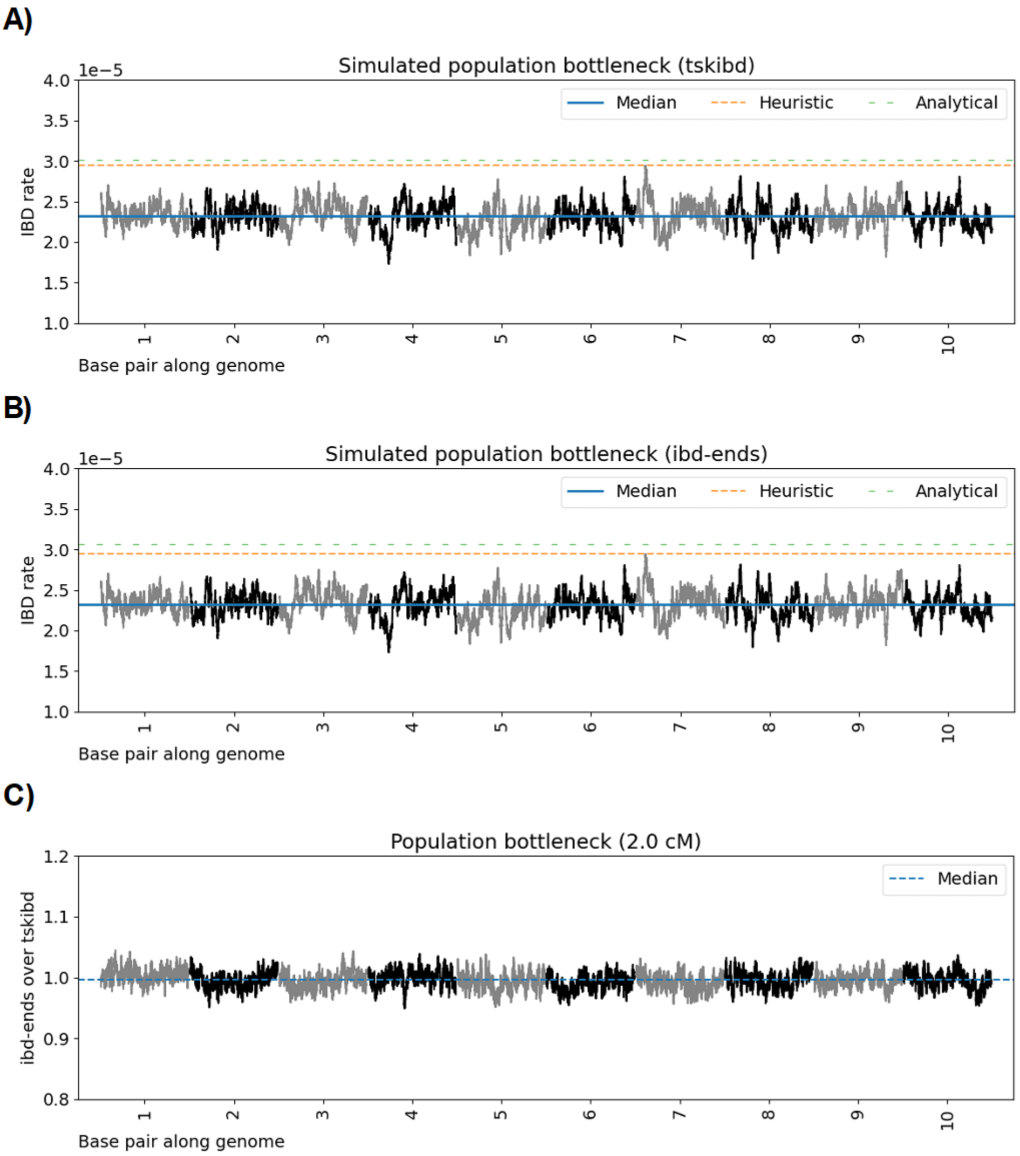

Figure S17: Genome-wide IBD rate scan in a simulated population bottleneck scenario. Line plots show $\geq$ 2.0 cM IBD rates (y-axis) for cM positions along ten simulated chromosomes. Scans are based on A) `tskibd` true IBD segments [6] or B) `ibd-ends` inferred IBD segments [24]. In C), we divide the IBD rates in B) from those in A). Each chromosome is 100 cM. The IBD rate is calculated every 0.02 cM. Data is based on twenty-five hundred diploid samples from the simulated population bottleneck demographic scenario. Horizontal dashed lines show (blue) the genome-wide median IBD rate, (orange) the heuristic threshold of four standard deviations above the median, and (green) the discrete-spacing analytical threshold). The family-wise significance level is 0.05.

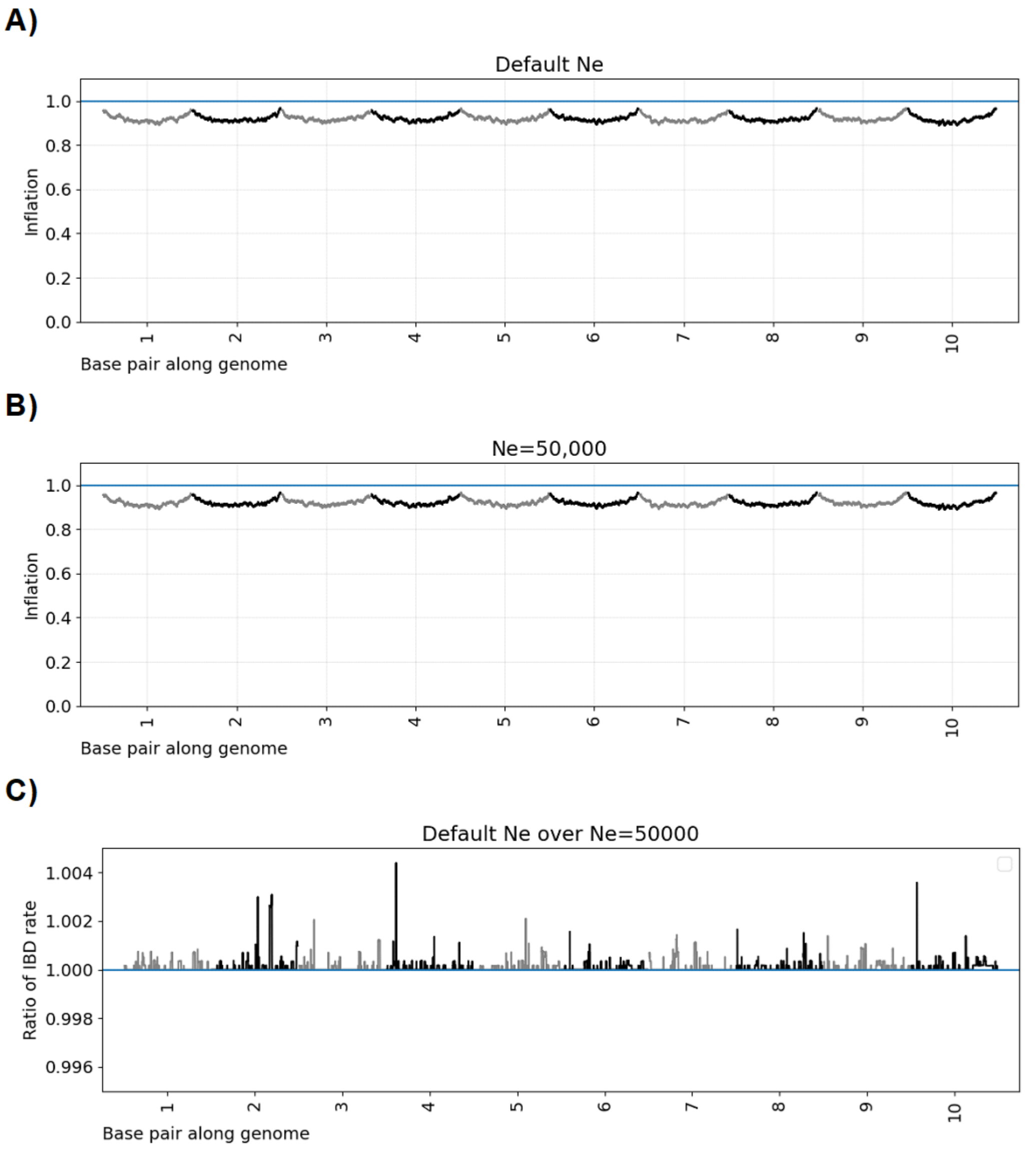

Figure S18: Genome-wide IBD rate scan in a simulated constant population size scenario. Line plots show inferred IBD rates over true IBD rates (y-axis) for cM positions along ten simulated chromosomes. Scans are based on using `ibd-ends`'s A) default `ne` setting versus B) `ne=50000`. In C), we divide the inferred IBD rates in A) and B). Each chromosome is 100 cM. The IBD rate is calculated every 0.02 cM. Data is based on twenty-five hundred diploid samples from the simulated scenario of a constant population of fifty thousand individuals. The IBD segment detection threshold is ≥ 2.0 cM.

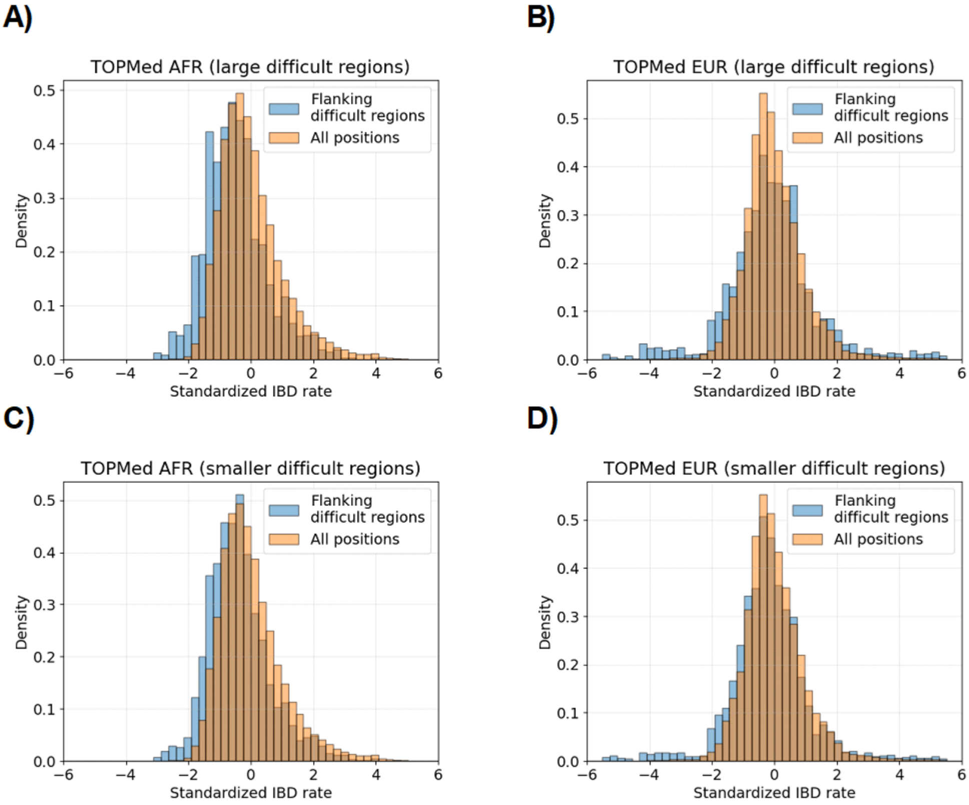

Figure S19: IBD rates flanking low mappability regions. Histograms show standardized IBD rates genome-wide (orange) and near low mappability regions (blue) in the TOPMed AFR A,C) and TOPMed EUR1 B,D) groups. We considered big low mappability regions from the GIAB problematic regions if they spanned A,B) more than 100 kb (referred to "large") or C,D) 50 kb (referred to as "small"). We concatenated "large" and "smaller" regions if they were within 50 and 25 kb of each other, respectively. By flanking, we mean that the IBD rates are up to 1 Mb directly to the left and right of a GIAB problematic region.

Supplemental tables

Family-wise level	Adjusted Analytical	Simulation	Bonferroni	FWER Analytical	Simulation
0.01	1.58e-6	2.01e-6	2.08e-7	0.008	0.012
0.05	9.23e-6	1.06e-5	1.04e-6	0.030	0.034
0.10	2.03e-5	2.29e-5	2.08e-6	0.066	0.078

Table S1: Significance levels and family-wise error rates after multiple-testing corrections with IBD segments $\geq$ 3.0 cM. Significance levels are adjusted for multiple testing based on scans over 10 chromosomes of size 100 cM and tests every 0.02 cM (50,000 total tests). The multiple-testing analytical and simulation-based thresholds are based on a fitted Ornstein-Uhlenbeck process. Each simulation has a different threshold as a result of estimating θ. Family-wise error rate (FWER) is the percentage of five hundred genome-wide scans with at least one statistically significant result. The demographic scenario is the population bottleneck.

Dataset	Chr	Rate (1e-4)	Region size (cM)	Position (Mb)	Genes	p value
TOPMed EUR1 (GRCh38)	2	1.37	7.94	134.84 (132.29-140.09)	*LCT*†	2.59e-187
	6	1.23	8.04	31.03 (23.91-36.38)	*MHC**†	2.14e-143
	17	0.71	3.44	37.68 (36.33-38.44)	*HNF1B**†	2.59e-31
	16	0.54	3.00	17.74 (16.73-18.72)	*XYLT1**	8.50e-13
	22	0.52	5.12	20.23 (19.15-21.10)	.	6.37e-11
	12	0.50	5.10	51.38 (48.80-53.20)	*KRT*†	1.74e-9
	15	0.48	2.28	31.30 (30.46-32.15)	*TRPM1**†	4.19e-8
UKBB white British 410k (GRCh37)	2	3.98	8.72	135.91 (132.86-141.34)	*LCT*†	$<$ 5e-324
	6	1.65	7.92	30.80 (23.91-36.34)	*MHC**†	2.63e-74
	15	1.42	3.50	30.94 (30.16-32.92)	*TRPM1**†	1.26e-47
	16	1.02	3.28	18.25 (16.37-18.95)	*XYLT1**	6.40e-16
	22	0.91	3.46	21.53 (20.98-21.61)	.	1.91e-10
	12	0.91	5.12	51.78 (49.38-53.76)	*KRT*†	2.04e-10
	17	0.83	1.26	36.18 (35.44-36.49)	*HNF1B**†	4.16e-7
UKBB Indian British (GRCh37)	2	1.41	5.12	136.97 (134.36-139.51)	*LCT*†	1.69e-36
	19	1.12	4.92	50.23 (48.47-50.74)	.	5.25e-16
	6	1.10	3.12	33.96 (33.02-36.34)	*MHC**†	4.70e-14
	16	0.96	2.80	18.06 (16.83-18.28)	*XYLT1**	1.65e-7
TOPMed AFR (GRCh38)	16	1.39	3.44	17.73 (16.45-19.09)	*XYLT1**	1.31e-63
	22	0.92	5.56	20.26 (18.95-21.10)	.	3.30e-21
	19	0.69	1.98	1.78 (1.72-2.10)	.	3.56e-9
	11	0.68	2.74	5.23 (3.83-5.75)	*HBB*†	4.83e-9
	5	0.37	1.56	9.44 (9.20-9.72)	*TAS2R1*	2.34e-7
UKBB Black British (GRCh37)	11	3.79	7.78	4.72 (2.76-6.92)	*HBB*†	2.78e-271
	16	2.52	4.14	17.40 (16.06-19.14)	*XYLT1**	4.12e-109
	22	1.28	4.74	21.54 (19.64-22.33)	.	1.68e-21
	5	0.88	1.64	9.62 (9.34-9.90)	*TAS2R1*	5.77e-8
	20	0.86	1.54	40.93 (39.47-40.99)	.	1.30e-7
	7	0.83	0.50	80.35 (80.08-80.40)	*SEMA3C*	7.61e-7

Table S2: Loci detected in the $\geq$ 3.0 cM selection scans. We report loci where identity-by-descent (IBD) rates exceed the discrete-spacing analytical thresholds of 0.45e-4, 0.81e-4, 0.93e-4, 0.61e-4, and 0.81e-4 for the TOPMed EUR1 ancestry, UKBB white British, UKBB Indian British, TOPMed AFR ancestry, and UKBB Black British sample sets, respectively. The maximum IBD rate is given for each locus. Physical positions for the location of the maximum IBD rate and the span of excess IBD rates are shown in megabases (Mb). We report the size in centiMorgan (cM) of each region, which is defined to be a contiguous stretch of IBD rates exceeding the genome-wide significance threshold. Pedigree-based recombination maps from Halldorsson et al. [83] and Bhérer et al. [85] aligned to the GRCh38 and GRCh37 reference genomes are used for inferring IBD segments in the TOPMed and UKBB sample sets, respectively. p values are calculated assuming the null model that IBD rates are normally distributed. Annotated genes or gene complexes are discussed in the main text. The signals with asterisks overlap hotspots of recurrent copy number variation and/or have multiple alternate locus sequences that have been added by the Genome Reference Consortium. The signals with daggers have previously been reported in the literature. The signals with dots are not discussed with respect to specific genes or gene complexes.

Dataset	Genes	Count of outlier clusters	Proportion of largest cluster	Proportion of second largest cluster	Proportion of all clusters
TOPMed AFR	*XYLT1*	4	2.1	1.4	5.1
UKBB Black British	*XYLT1*	6	2.2	1.2	6.5
	HBB	3	8.6	1.6	11.5
	SEMA3C	3	5.7	1.0	7.7
UKBB Indian British	*LCT*	2	15.2	1.9	17.1
	OCA2	12	1.6	0.6	5.2
	MHC	12	3.4	2.4	13.2
	XYLT1	15	0.6	0.5	4.8
TOPMed EUR	*LCT*	1	65.1	.	65.1
	*OCA2**	1	68.5	.	68.5
	OAS	8	9.4	3.1	19.3
	MHC	7	12.4	8.8	29.5

Table S3: Fine mapping candidate alleles, or lack thereof, for positive selection. We applied the Temple et al. [14] suite of methods to detect unusually large clusters sharing IBD haplotypes. We used the default parameter settings. The sample proportions in the IBD clusters are with respect to the entire sample. We only investigated some loci to compare the signal at *XYLT1* against known positively selected loci *LCT* and *OCA2* and the complex *MHC* region under balancing selection. The *OCA2* result (asterisk) is from the Temple et al. [14] analysis of the Women's Health Initiative data in TOPMed. A more exhaustive analysis of the TOPMed EUR1 results is in Temple et al. [14]. For *LCT* and *OCA2*, there is broad consensus in the literature supporting the selective sweep hypothesis. The dot denotes that there there is not a second excess IBD sharing cluster.

Supplemental acknowledgements

We gratefully acknowledge the individual studies and participants who provided biological samples and data for the TOPMed project. Funding for the Barbados Asthma Genetics Study (BAGS) was provided by the National Institutes of Health (NIH) R01HL104608, R01HL087699, and HL104608 S1. The Mount Sinai BioMe Biobank (BioMe) has been supported by The Andrea and Charles Bronfman Philanthropies and in part by funds from the NHLBI and the National Human Genome Research Institute (NHGRI) (U01HG00638001; U01HG007417; X01HL134588); genome sequencing was funded by contract HHSN268201600037I. The Cleveland Clinic Atrial Fibrillation study (CCAF) was supported by NIH grants R01 HL 090620 and R01 HL 111314, the NIH National Center for Research Resources for Case Western Reserve University and Cleveland Clinic Clinical and Translational Science Award UL1-RR024989, the Cleveland Clinic Department of Cardiovascular Medicine philanthropy research funds, and the Tomsich Atrial Fibrillation Research Fund; genome sequencing was supported by R01HL092577. The Framingham Heart Study (FHS) was supported by contracts NO1-HC-25195, HHSN268201500001I, and 75N92019D00031 from the NHLBI and grant supplement R01 HL092577-06S1; genome sequencing was funded by HHSN268201600034I and U54HG003067. The Hypertension Genetic Epidemiology Network Study (HyperGen) is part of the NHLBI Family Blood Pressure Program; collection of the data represented here was supported by grants U01 HL054472, U01 HL054473, U01 HL054495, and U01 HL054509; genome sequencing was funded by R01HL055673. The Jackson Heart Study is supported and conducted in collaboration with Jackson State University (HHSN268201300049C and HHSN268201300050C), Touga-

loo College (HHSN268201300048C), and the University of Mississippi Medical Center (HHSN268201300046C and HHSN268201300047C) contracts from NHLBI and the National Institute for Minority Health and Health Disparities (NIMHD); genome sequencing was funded by HHSN268201100037C. The My Life, Our Future samples (MLOF) and data are made possible through the partnership of Bloodworks Northwest, the American Thrombosis and Hemostasis Network, the National Hemophilia Foundation, and Bioverativ; genome sequencing was funded by HHSN268201600033I and HHSN268201500016C. The Venous Thromboembolism project (VTE) was funded in part by grants from the NIH, NHLBI (HL66216 and HL83141), and the NHGRI (HG04735). The Vanderbilt Genetic Basis of Atrial Fibrillation study (VUAF) was supported by grants from the American Heart Association (EIA 0940116N) and grants from the National Institutes of Health (HL092217, U19 HL65962, and UL1 RR024975), and by CTSA award (UL1TR000445) from the National Center for Advancing Translational Sciences; genome sequencing was funded by R01HL092577. The Women's Health Initiative program (WHI) is funded by NHLBI through contracts 75N92021D00001, 75N92021D00002, 75N92021D00003, 75N92021D00004, 75N92021D00005; genome sequencing was funded by HHSN268201500014C.
