# Supplementary material for: Multiple-testing corrections in selection scans using identity-by-descent segments
Source: Am J Hum Genet. 2025 Sep 26;112(11):2751–71. doi: 10.1016/j.ajhg.2025.09.004 (PMC12668784; doi:10.1016/j.ajhg.2025.09.004)
Supplement: Document S2. Article plus supplemental information [file mmc2.pdf]

ARTICLE

Multiple-testing corrections in selection scans using identity-by-descent segments

Authors

Seth D. Temple, Sharon R. Browning

Correspondence

sethtem@umich.edu

By modeling correlations along the genome, we determined significance levels for a scan of excess identity-by-descent sharing that help avoid reporting false positive results. Some large signals observed in multiple different ancestry groups are unlikely to be examples of recent genetic adaptation but instead concentrated around structural variants.

Temple & Browning, 2025, The American Journal of Human Genetics *112*, 2751–2771

November 6, 2025 © 2025 The Author(s). Published by Elsevier Inc. on behalf of American Society of Human Genetics.

https://doi.org/10.1016/j.ajhg.2025.09.004

CellPress

Multiple-testing corrections in selection scans using identity-by-descent segments

Seth D. Temple[1,2,3,*] and Sharon R. Browning[4]

Summary

Failing to correct for multiple testing in selection scans can lead to false discoveries of recent genetic adaptations. The scanning statistics in selection studies are often too complicated to theoretically derive a genome-wide significance level or empirically validate control of the family-wise error rate (FWER). By modeling the autocorrelation of identity-by-descent (IBD) rates, we propose a computationally efficient method to determine genome-wide significance levels in an IBD-based scan for recent positive selection. In whole-genome simulations, we show that our method has approximate control of the FWER and can adapt to the spacing of tests along the genome. We also show that these scans can have more than 50% power to reject the null model in hard sweeps with a selection coefficient greater than or equal to 0.01 and a sweeping allele frequency between 25% and 75%. Many human genes and gene complexes have statistically significant excesses of IBD segments in thousands of samples of African, European, and South Asian ancestry groups from the Trans-Omics for Precision Medicine project and the United Kingdom Biobank. Among the significant loci, two excess IBD signals in regions enriched for deletions are shared across ancestry groups.

Introduction

Positive natural selection has been suggested to be the primary mechanism of phenotypic adaptation.[1] Many reported instances of positive selection in human populations concern adaptive evolution on immunity-related genes.[2,3] There is also evidence in bacterial, parasite, and insect vector populations of genic selection to evade public health efforts.[4–6] These examples indicate that the adversarial dynamics between macro-organisms and their microbial pathogens may be a powerful force driving genetic changes in populations. Learning about these genetic changes could be helpful in the design of new vaccines, therapeutics, and interventions in the environment.

Decades of genetics and evolution research have provided many methods to detect positive selection. In general, a statistic is devised to capture different alternative hypotheses from the neutral theory of Kimura[7] or the slightly deleterious theory of Ohta,[8] and then the statistic is calculated across the genome to scan for significant evidence against a null model. Some examples of alternative models are selective sweeps[5,9–12] and balancing selection.[13] Vitti et al.[1] and Temple et al.[14] categorize these methods into several groups: amino acid substitution rates,[15,16] population differentiation,[17,18] frequency,[19–21] linkage disequilibrium (LD),[14,22–31] coalescent,[32–35] approximate Bayesian computation,[36] time series,[37,38] and machine learning-based methods.[39–44] On the one hand, these methods are designed to detect natural selection at different evolutionary timescales or under different mechanisms. On the other hand, the lack of statistical models may have led to the development of many *ad hoc* summary statistics.[45] For instance, some methods clarify that summary statistics a few standard deviations above a genome-wide mean do not have p values[22,31] and, equally so, no adjustment for multiple testing.

We aim to develop a hypothesis testing framework for the selection statistic proposed in Browning and Browning[24] and studied in Temple et al.[14] One major approach to developing multiple-testing adjustments is to control the family-wise error rate (FWER). The FWER is the probability of rejecting the null hypothesis one or more times when the null hypothesis is true,[46] whose control is more conservative than that of the false discovery rate.[47] Because concluding that a locus is/was under strong selection could have a societal impact if misappropriated,[48] we opt to derive FWER-based multiple-testing corrections.

The p value threshold of 5e−8 is commonly used in genome-wide association studies (GWASs). The 5e−8 genome-wide significance level comes from the Bonferroni correction at the 0.05 significance level based on an assessment of the number of effective hypothesis tests in human genotype array data from the early 2000s.[49,50] Some population genetics studies use this *de facto* significance level, even though their test statistic and data differ from those of the GWAS study design. For instance, in their selection tests, Field et al.[20] and Speidel et al.[33] used the 5e−8 p value threshold. The effective number of tests and the multiple-testing correction depend on the test statistic and its correlation along the genome.

Permutation or simulation-based approaches can provide interpretable p values and control the FWER under valid permutation or simulation frameworks. Still, these procedures can be computationally intensive and

[1]Department of Statistics, University of Washington, Seattle, WA, USA; [2]Department of Statistics, University of Michigan, Ann Arbor, MI, USA; [3]Michigan Institute for Data and AI in Society, University of Michigan, Ann Arbor, MI, USA; [4]Department of Biostatistics, University of Washington, Seattle, WA, USA

*Correspondence: sethtem@umich.edu
https://doi.org/10.1016/j.ajhg.2025.09.004.
© 2025 The Author(s). Published by Elsevier Inc. on behalf of American Society of Human Genetics.
This is an open access article under the CC BY license (http://creativecommons.org/licenses/by/4.0/).

challenging to design.[51–56] To remain feasible, some of these simulation-based approaches were applied to sample sizes less than a few thousand,[51,55] or they leveraged the fact that Wald and score statistics from linear models are asymptotically normally distributed.[52,54] Implementing a simulation-based approach can be infeasible for selection tests that are already computationally intensive in one scan.

Another approach is to model the test statistics under the null hypothesis as a stochastic process and use the properties of that process to determine the threshold. In an identity-by-descent (IBD) mapping study, Browning and Thompson[51] approximated transitions between IBD and non-IBD states as a Markov process and derived an analytical genome-wide significance threshold under their model. In an admixture mapping study, Grinde et al.[54] approximated their Wald test statistics as an Ornstein-Uhlenbeck (OU) process and then calculated the genome-wide significance level with an analytical solution.[56,57] The Siegmund and Yakir[56] calculation of the genome-wide significance level applies to any scan that can be reasonably modeled as an OU process.

Multiple testing addresses scientific discovery in a single study, whereas much of the consensus scientific progress comes from replicated findings. For example, most scans for recent positive selection in European ancestry populations have detected the *LCT* (MIM: 603202) signal,[58] which can be as large as thirty-five standard deviations greater than the median of a genome-wide scanning statistic.[14] Indeed, many scans have detected several overlapping selection signals in European ancestry populations.[24,28,31–33,37,38] Fewer studies have explored recent positive selection in non-European ancestry populations. Albrechtsen et al.[23] identified the major histocompatibility complex (*MHC*) region as having extreme rates of alleles inferred to be IBD in all human populations. Taliun et al.[59] used the Field et al.[20] method to identify a few loci putatively under recent selection in African and East Asian ancestry samples. In yet another example, Granka et al.[60] enumerated some extreme values of the cross-population extended haplotype homozygosity statistic[17] found in African ancestry populations, but without a multiple-testing adjustment, they exercised caution in the interpretation of their findings. Temple et al.[14] advise that analyzing selection in non-European ancestry samples should proceed with multiple-testing adjustments.

To control the FWER when scanning the genome for excess IBD rates, we propose analytical and simulation-based significance thresholds from an estimated OU process model.[14,24] We show that the adjusted significance thresholds should approximately control the FWER under some central limit theorem conditions.[61] The IBD rate scan is computationally efficient; hence, we can measure its FWER in simulation studies. We also demonstrate the effects of various analysis decisions on the empirical FWER and statistical power, including user-defined centimorgan (cM) spacings and IBD segment detection thresholds. We used a ≥ 2.0 cM segment detection threshold in our real data analyses, which only provides the signal from the past few hundred generations.[14,62] We show that the heuristic four standard deviations above the autosome-wide median threshold used in the Browning and Browning[24] and Temple et al.[14] studies may have been reasonable for European ancestry populations but that the genome-wide significance threshold should be more stringent for some African ancestry populations. Nevertheless, after adjusting for multiple testing, we observed fewer than twelve signals of recent positive selection in any given cohort.

Material and methods

Hypothesis testing framework

First, we define the implicit hypothesis test in the IBD rate scan.[14,24] When modeling the spatial process, we use the same mathematical notation as Temple and Thompson,[61] with minor revisions. Let $Y_{a,b}(m)$ be the indicator that the IBD segment between haplotypes a and b is longer than a detection threshold and overlaps the mth focal position. The IBD rate at the mth locus is $\overline{Y}_m = f(n)^{-1}\sum_{(a,b)} Y_{a,b}(m)$, where $f(n) = 2n(2n-1)/2 - n$ in diploids and $f(n) = \binom{n}{2}$ in haploids. The hypothesis test we consider is

$$H_0 : \mathbb{E}[\overline{Y}_m] = \mu_0 \qquad \text{(Equation 1)}$$

$$H_1 : \mathbb{E}[\overline{Y}_m] > \mu_0, \qquad \text{(Equation 2)}$$

where μ_0 is a genome-wide mean IBD rate around a locus. This null model is consistent with no positive selection. The alternative model is consistent with positive selection *or* other evolutionary mechanisms.

Let $\hat{\mu}_{1:M}$ and $\hat{\sigma}_{1:M}$ be the sample mean and sample standard deviation of M IBD rates along the genome:

$$\hat{\mu}_{1:M} := M^{-1}\sum_{m=1}^{M}\overline{Y}_m; \qquad \text{(Equation 3)}$$

$$\hat{\sigma}_{1:M} := \sqrt{(M-1)^{-1}\sum_{m=1}^{M}\left(\overline{Y}_m - \hat{\mu}_{1:M}\right)^2}. \qquad \text{(Equation 4)}$$

Browning and Browning[24] and Temple et al.[14] have suggested a heuristic threshold of $\hat{\mu}_{1:M} + 4 \times \hat{\sigma}_{1:M}$ as strong evidence against the null model. (They used the genome-wide median, not the mean, which can be more robust to outliers like *LCT* selection.) Under asymptotic conditions on sample size, population demography, and the detection threshold, the standardized IBD rate $\tilde{Z}_m$ around the mth locus is normally distributed.[61] The heuristic threshold corresponds to a significance level of $1 - \Phi(4) = 3.17 \times 10^{-5}$.

We use the same test statistic as Browning and Browning[24] and Temple et al.,[14] except we adapt the number of standard deviations to the correlation structure in a distinct sample:

$$\begin{array}{ll} \overline{Y}_m - \hat{\mu}_{1:M} > z_{\alpha^*} \times \hat{\sigma}_{1:M} & \rightarrow \text{reject } H_0 \\ \overline{Y}_m - \hat{\mu}_{1:M} \leq z_{\alpha^*} \times \hat{\sigma}_{1:M} & \rightarrow \text{fail to reject } H_0. \end{array} \qquad \text{(Equation 5)}$$

This test corresponds to a one-sample one-sided t test or a z test when the number of tests M is large. The significance level α^*

comes from a multiple-testing correction at the family-wise significance level α, and z_{α^*} is the corresponding standard normal quantile.

To determine multiple-testing corrections, we model standardized IBD rates along the genome

$$\{\bar{\bar{Z}}\}_{1:M} := \frac{(\{\bar{Y}\}_{1:M} - \widehat{\mu}_{1:M})}{\widehat{\sigma}_{1:M}}, \quad \text{(Equation 6)}$$

as a correlated OU process. This model has previously been used to determine multiple-testing corrections in admixture mapping[53,54] and linkage analysis.[57] The OU process is normally distributed at every point, is spatially homogeneous, and has the first-order Markov property. Assuming normality at every point is supported by the Temple and Thompson[61] central limit theorems and may be reasonable in human genetics studies. Spatial homogeneity is an assumption consistent with, but does not require, (nearly) neutral evolution[7,8,13] and uniform IBD segment detection accuracy. Background selection could explain 60% of the variation in nucleotide diversity,[63] which violates the neutral model. We thus assume that this violation does not substantially impact genome-wide IBD patterns. Compared to the Grinde et al.[54] admixture mapping statistics, which are provably Markov, the IBD rate along the chromosome is not a Markov process (Temple[62] gives a simple counterexample). Therefore, we assume that the IBD rate process is nearly Markov, at least so much so that the violation does not affect our multiple-testing corrections.

The standard OU process has a specific correlation pattern. Namely, if the genetic distance between consecutive focal positions is set to be constant Δ, then the covariance between standardized IBD rates $\bar{\bar{Z}}_{m_1}$ and $\bar{\bar{Z}}_{m_2}$ at different loci is

$$\text{Cov}(\bar{\bar{Z}}_{m_1}, \bar{\bar{Z}}_{m_2}) = \exp(-\theta \cdot \Delta (m_2 - m_1)), \quad \text{(Equation 7)}$$

where θ is an exponential decay parameter. The exponential decay parameter θ is not known for the IBD rate process but must be estimated, whereas θ is the time of admixture in Grinde et al.,[54] which can be estimated or assumed from prior knowledge.

Multiple-testing corrections

Analytical approximation

To control the FWER, we must determine the multiple-testing quantile z_{α^*} such that $P(\max_m \bar{\bar{Z}}_m \geq z_{\alpha^*}) = \alpha$. Let L be the total length of the genome (in morgans), C the number of chromosomes, and Φ and ϕ the cumulative distribution and density functions of the standard normal random variable, respectively. Siegmund and Yakir[56] provide the FWER-based analytical approximation

$$P\left(\max_{1 \leq m \leq M} \bar{\bar{Z}}_m \geq z\right) \approx 1 - \exp\left(-C[1 - \Phi(z)] - \theta \cdot L \cdot z \cdot \phi(z) \cdot \nu\left(z\{2\theta\Delta\}^{1/2}\right)\right), \quad \text{(Equation 8)}$$

where $\nu(\cdot)$ accommodates the discretization of the continuous stochastic process. When the morgan step size $\Delta \rightarrow 0$ (the continuous process), $\nu(0) = 1$. We determine z_{α^*} from Equation 8 with a root solver, which runs in seconds. This approach is an example of finding the first hitting time of a stochastic process.

Simulation-based approach

Another way to control the FWER is to simulate the OU process for known or estimated θ. Let J be the number of simulations and $M := \lfloor L \div \Delta \rfloor$. The simulation approach goes as follows.

Algorithm 1:

(1) Let $\mathbf{z}_{1:J}$ be an empty vector.
(2) For j in 1 to J:
 (a) Draw $z_1 = Z_1 \sim N(0,1)$.
 (b) For m in 2 to M:
  i. Draw
$z_m = Z|z_{m-1} \sim N(z_{m-1} \cdot \exp(-\theta \cdot \Delta), 2 - 2 \cdot \exp(-\theta \cdot \Delta))$.
 (c) Append $\max_m z_m$ to the vector $\mathbf{z}_{1:J}$.
(3) Return the $(1-\alpha)$% quantile of $\mathbf{z}_{1:J}$.

For family-wise significance levels like 0.01 or 0.05, this whole-genome simulation approach requires a few thousand simulations and runs within a few minutes (depending on the genome length L) on an Intel 2.60 GHz core processing unit (CPU). This multiple-testing correction is valid when the true model is the OU process. A precise algorithm would simulate individual OU processes for different chromosome lengths, but for simplicity, we simulate a single chromosome of the total genome length instead.

Estimator of the exponential decay parameter

Before standardizing the IBD rates, we adjust for extreme outliers that could be present in real genetic data. First, we compute an initial genome-wide median IBD rate plus four standard deviations. Second, we compute a revised genome-wide mean IBD rate and standard deviation, excluding the IBD rates that exceed the initial threshold. We standardize the IBD rates with the revised mean and standard deviation estimates. This step is suitable for the reproducible workflow of Temple et al.,[14] whereas filtering out known exceptions like *LCT* selection in European ancestry populations is less amenable to automation.[64]

To estimate the exponential decay parameter θ, we regress estimated autocovariances on genetic position. We apply linear interpolation to the recombination map to hold the spacings between IBD rates constant. Then, we estimate the covariance between standardized IBD rates at genetic positions Δ times some integer constant apart, excluding IBD rates that exceed the initial threshold. The integer scalars increment by one until the covariance is between positions a maximum of 4.0 cM apart. We fit a simple log-linear model with no intercept, where the integer-scaled Δs are the covariates and the estimated autocovariances are the response variables. The fitted slope parameter is an estimator $\widehat{\theta}$ of the exponential decay parameter.

Simulating IBD rate processes

Null hypothesis model

We evaluated control of the FWER and the accuracy of our estimator $\widehat{\theta}$ with large-scale coalescent simulations. We used `msprime`[65] to simulate ten chromosomes, each of length 100 cM, and we used `tskibd`[6] to get IBD segment lengths longer than 2.0 and 3.0 cM from the tree sequence output by `msprime`. We set the constant recombination rate to 1e−8. We considered previously defined demographic scenarios of a population bottleneck, a constant population of 50,000 individuals in size, and staged exponential growth.[14,61,62,66] The demographic scenario affects the exponential decay parameter θ. Unless otherwise specified, our default demographic scenario was the population bottleneck.

We estimated θ from the autocovariances of simulated IBD segments, and then we used the estimate $\widehat{\theta}$ to calculate our

multiple-testing adjusted thresholds. For these calculations of the genome-wide significance level, we considered different step sizes of 0.02, 0.05, and 0.10 cM. Unless otherwise specified, the default step size was 0.02 cM. The estimator $\widehat{\theta}$ should be agnostic to the cM spacing, but the genome-wide significance level should decrease monotonically with the cM spacing.

To empirically measure the FWER, we considered 500 simulations of entire genomes from 2,500 diploids. The FWER was calculated as the percentage of the 500 null model simulations with at least one significant result. We explored the family-wise significance levels of 0.01, 0.05, and 0.10. Unless otherwise specified, we used the 0.05 family-wise significance level. We used the discrete-spacing analytical approximation as our default multiple-testing correction.

The data from our simulations amounts to 1 terabyte (TB) compressed disk storage, predominantly due to the `msprime` tree sequences. We were unable to make VCF marker data for all our simulations and, therefore, to infer IBD segments, which would create many more TB of additional disk memory. In Appendix A, we analyzed the accuracy of IBD segment detection in VCF marker data.

Selective sweep alternative model

To calculate statistical power, we considered hard sweeps as the alternative model. This evolutionary scenario concerns a single advantageous allele increasing in frequency, with the rate of change parameterized by the selection coefficient s.[67–69] For the population bottleneck and staged exponential growth scenarios, we simulated IBD segments overlapping a focal point for hard sweeps with $s \geq 0.006$ and current-day allele frequency $p(0) = 0.10, 0.25, 0.50, 0.75,$ and 0.90 with the Temple et al.[66] algorithm. Based on the results of Temple et al.,[14] we believe that the algorithm in Temple et al.[66] simulates IBD rates around a locus similar to those drawn from tree sequences by `tskibd`, which itself has not been independently benchmarked. For the constant population size scenario, we did consider tree sequences, and therefore `tskibd` segments, simulated with positive selection, which is an `msprime` feature only available for constant populations.[65,70]

Power was calculated as the proportion of our selective sweep simulations (alternative hypotheses) in which we reject the null model. The threshold in our power calculations was the average of the multiple-testing adjusted thresholds in our 500 neutral simulations (at the 0.05 family-wise significance level). We estimated power using 200 simulations for each pair of selection coefficient and current-day sweeping allele frequency. Because the IBD rates mainly increase when allele frequencies rapidly change in the most recent 100 generations, our test should have similar power in soft-sweep simulations from starting frequencies less than 5%.[14,62]

Pre-processing genetic data

In our study, we focused on selection scans in African, European, and South Asian ancestry groups from the Trans-Omics for Precision Medicine (TOPMed) project[59] and the United Kingdom Biobank (UKBB).[71] The TOPMed data that we analyzed include more than 30,000 whole-genome sequences from multiple ethnic groups represented in the US, combining samples from various cohort studies. We used the 318,858,817 filtered autosomal markers from the TOPMed data phased with `Beagle` 5.2 in Browning et al.[72] The UKBB is a biomedical database containing genotype array data from nearly 500,000 participants between 40 and 69 years of age. We used the 711,651 filtered autosomal markers from the UKBB single-nucleotide polymorphism (SNP) array data in Browning et al.[72] The TOPMed and UKBB datasets were kept separate in all analyses.

TOPMed

We analyzed the whole-genome sequences of multiple ancestry groups inferred by Temple et al.[14] These ancestry groups were defined by principal-component analysis (PCA)[73,74] and validated with `ADMIXTURE`.[75] Individuals inferred to be third-degree or closer relatives were excluded.[14] One of our subsets is the 13,778 European ancestry samples studied by Temple et al.,[14] which we now refer to as the EUR1 ancestry group.

Another European ancestry group we defined is EUR2, comprising 1,719 samples whose principal components are near but distinct from those of the samples in the EUR1 group. 64% of these samples come from the BioMe Biobank cohort study at Mt. Sinai School of Medicine in New York City, which is a dataset known to contain many samples inferred to have Ashkenazi Jewish ancestry.[76] For this group, we inferred a demographic history that sharply drops to an effective size as small as 1,000 in the most recent thirty generations (`IBDNe` using $\geq$2.0 cM IBD segments[77]). In an Ashkenazi Jewish sample, Carmi et al.[78] inferred a recent bottleneck of the effective size of a few hundred diploids, which Tian et al.[79] say is consistent with their demographic inference of a Framingham Heart Study subset. Carmi et al.[78] state that the Ashkenazi Jewish population is most genetically similar to European and Middle Eastern populations, which is consistent with the Temple et al.[14] PCA and the `fastSTRUCTURE` analysis[80] done by Wu et al.[76]

Using the first principal component, we defined an inferred African ancestry group (AFR) of 1,737 samples. Based on the `ADMIXTURE` validation study of Temple et al.,[14] these samples have minimum and mean global ancestry proportions of 0.88 and 0.93, respectively, with respect to the Yoruba in Ibadan, Nigeria (YRI) reference panel.[81,82] 54% of these samples self-report as Black or African American, and 46% self-report as other. Only samples from the Barbados Asthma Genetics Study (BAGS), Jackson Heart Study (JHS), and Hypertension Genetic Epidemiology Network Study (HyperGen) cohorts are represented in this subset. Afro-Caribbeans living in Barbados are in the BAGS study, whereas African Americans living in the southern continental US are in the JHS and HyperGen studies.

To detect IBD segments in the TOPMed sample sets, we used the algorithm parameters in the Temple et al.[14] workflow. Note that `ibd-ends` refines the endpoints of IBD segment calls in light of genotyping error rates and low-marker-density regions.[24] In the EUR1 ancestry group, we used the IBD segments previously inferred by Temple et al.[14] We performed preliminary analyses of chromosomes 19–22 with `ibd-ends`[24] to get estimates of the error rate parameter, eventually specifying the error rate `err = 1.5e-4` for all three groups. All TOPMed analyses used the 2019 pedigree-based genetic map from deCODE Genetics.[83] This recombination map is aligned to the GRCh38 reference genome.

UKBB

We also analyzed subsets of the UKBB samples who self-report as various non-White ethnic groups. The first subset includes 5,660 individuals who self-report as Indian British.[71] The second subset consists of 3,202 individuals who self-report as Black British (African in Bycroft et al.[71]). We phased the sample sets individually with `Beagle` v.5.4. Based on genetic relatedness inference in Cai et al.,[84] we removed closely related individuals from both subsets, resulting in 5,374 Indian British and 3,146 Black British samples.

Table 1. Genome-wide significance levels and family-wise error rates after multiple-testing corrections

Family-wise level	Genome-wide analytical	Simulation	Bonferroni	FWER analytical	Simulation
0.01	1.08e−6	1.30e−6	2.08e−7	0.024	0.028
0.05	6.24e−6	7.03e−6	1.04e−6	0.088	0.098
0.10	1.36e−5	1.49e−5	2.08e−6	0.140	0.146

Family-wise significance levels are adjusted for multiple testing based on scans over ten chromosomes, each of a size of 100 cM, and tests every 0.02 cM (50,000 total tests). The multiple-testing analytical and simulation-based thresholds are based on a fitted Ornstein-Uhlenbeck process. The family-wise error rate (FWER) is the percentage of 500 genome-wide scans with at least one statistically significant result. The demographic scenario is the population bottleneck. The IBD segment detection threshold is 2.0 cM.

We also analyzed the 408,891 UKBB White British samples previously studied in Browning and Browning.[24] (The group definition "White" comes from a combination of self-reported British ethnic background and similar scores in a PCA.[71]) The SNP array data were previously phased with `Beagle 5.2`, as described in Browning et al.[72]

To detect IBD segments in the UKBB sample sets, we modified our `hap-ibd` settings to `min-seed` = `1.8`, `min-extend` = `0.5`, `min-output` = `1.8`, and a minor-allele frequency of 0.001. We have not explored the accuracy of these settings in simulated array data. Still, we show in our results that our analyses of array data are consistent with our analyses of sequence data and with the existing literature on some selected loci. In the White British, Indian British, and Black British groups, we performed preliminary analyses of chromosomes 19–22 with `ibd-ends` to get estimates of the error rate parameter, eventually specifying the error rate `err` = `3.0e−4` for all groups. All UKBB analyses used the Bhérer et al.[85] pedigree-based genetic map. This recombination map is aligned to the GRCh37 reference genome.

Results

Simulated OU processes

We conducted a simple validation study to determine if the discrete-spacing analytical approximation and simulation-based genome-wide significance levels control the FWER when data are simulated from an OU process. The simulation settings are shown in Figures S1 and S2. Figure S1 shows that estimates $\hat{\theta}$ of the exponential decay parameter are approximately equal to the true value when $30 \leq \theta \leq 90$ and the genome size is $\geq$ 400 cM. For $\theta = 15$ and genome sizes less than or equal to 1,000 cM, some estimates $\hat{\theta}$ are close to or exactly 0, which would correspond to autosomal IBD rates with a positive correlation of 1. Figure S2 shows FWERs using the multiple-testing corrections at a family-wise significance level of 0.05. The FWERs from the discrete-spacing analytical approach are between 0.04 and 0.05 and less than 0.03 when $\theta \geq 30$ and $\theta = 1$, respectively. Grinde et al.[54] also find that the discrete-spacing analytical approximation is conservative when $\theta \approx 10$. The FWERs from the simulation-based approach are approximately 0.05 for all θ. We thus recommend using the simulation-based approach if $\theta \leq 20$. While the discrete-spacing analytical approach may be slightly conservative compared to the simulation-based approach, simulating 500 OU processes of a size equal to the 22 human autosomes can take as much as 10 min on an Intel 2.60 GHz CPU.

Simulated IBD rate processes

Estimating the exponential decay parameter

The boxplots in Figure S3 show the percentiles of estimates $\hat{\theta}$ using IBD segments $\geq$ 2.0 and $\geq$ 3.0 cM from data simulated under the null hypothesis with the population bottleneck scenario. Regardless of the step size Δ, the distribution of estimates $\hat{\theta}$ is the same, which is expected. The medians of estimates $\hat{\theta}$ for the $\geq$ 2.0 and $\geq$ 3.0 cM processes are roughly 62.5 and 40, respectively. As θ increases and while holding the genetic distance between two positions constant, the covariance between the two IBD rates decreases, which could be interpreted as fewer detectable IBD segments overlapping nearby loci on average. Estimates for θ are larger in the $\geq$ 2.0 cM scan versus the $\geq$ 3.0 cM scan because an IBD segment $\geq$ 2.0 cM is less likely to also overlap the next focal point than an IBD segment of $\geq$ 3.0 cM.

For the staged exponential growth scenario, the medians of estimates $\hat{\theta}$ are 74.75 and 56.78 for the $\geq$ 2.0 and $\geq$ 3.0 cM IBD rate processes, respectively. For the population of a constant size of 50,000 diploid individuals, the medians of estimates $\hat{\theta}$ are 58.84 and 44.97 for the $\geq$ 2.0 and $\geq$ 3.0 cM IBD rate processes, respectively. We expect different true θ and therefore different estimates $\hat{\theta}$ because demography influences the IBD segment length distribution.[62,77,84]

FWERs

Table 1 reports the multiple-testing adjusted significance levels and the empirical FWERs for the discrete-spacing analytical approximation and simulation-based approaches in the $\geq$ 2.0 cM IBD rate processes. The adjusted significance levels from the analytical and simulation-based approaches are nearly an order of magnitude larger than those using the Bonferroni correction.[62] At the 0.05 family-wise significance level, the FWERs of our analytical and simulation-based approaches are inflated by more than 150%. In contrast, the FWERs of the Bonferroni method with testing every 0.02 cM are deflated.[62] At the 0.10 family-wise significance level, the average standard deviations above the mean of the analytical and simulation-based approaches are 4.196 and 4.176. Temple and Thompson[61] give one plausible explanation for the

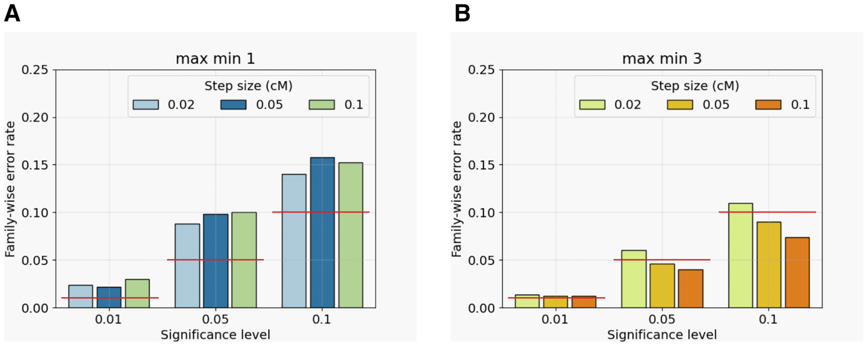

Figure 1. Family-wise error rates for genome-wide hypothesis testing in null model simulations
Bar plots show family-wise error rates (y axis) using true IBD segments ≥ 2.0 cM from simulated IBD rate processes. The hypothesis testing method is the discrete-spacing analytical approximation. In each non-overlapping window of size (A) 1 or (B) 3 marginal test statistics, we compute the minimum of IBD rates at each step, and the test is if the maximum over all windows is less than or greater than the multiple-testing quantile. There are 500 simulations for each combination of significance level (x axis) and step size (colors in legend). Family-wise significance levels are denoted with horizontal red lines. The demographic model is the population bottleneck. The amount of data for each simulation is equal to ten chromosomes, each of a uniform length of 100 cM.

anti-conservativeness of the hypothesis test with ≥ 2.0 cM segments, which is that the upper tail of the IBD rate's distribution may be heavier than the upper tail of a normal distribution.

Table S1 reports the adjusted significance levels and FWERs of the multiple-testing approaches using the 3.0 cM threshold. In this case, the IBD rate overlapping a locus may be better approximated by a normal distribution than in the ≥ 2.0 cM selection scan (conditions on the detection threshold in Temple and Thompson[61]). The FWERs of the analytical and simulation-based approaches are indeed conservative in the ≥ 3.0 cM excess IBD rate scan. We thus remark that there are two counteracting factors affecting FWER control: the multiple-testing adjustments are conservative in true OU processes (Figure S2), but the test could be anti-conservative if the OU process is a poor approximation for the IBD rate process.

For the anti-conservative ≥ 2.0 cM excess IBD rate scan, we considered modifying the test to explore whether the significant results *barely* exceed the threshold. We calculated at each locus the minimum of its value and the flanking values to its left and right. Next, we calculated the maximum over the entire genome of these aggregated minimum values:

$$\max_{1 \leq m \leq M} \min\{\bar{\bar{Z}}_{m-1}, \bar{\bar{Z}}_m, \bar{\bar{Z}}_{m+1}\}. \qquad \text{(Equation 9)}$$

Figure 1 shows that FWERs decrease when using the max-min statistic with the same threshold as the original scan. This result indicates that a considerable proportion of the family-wise errors correspond to marginally significant results. We used the max-min statistic to diagnose the behavior of our anti-conservative scan, but in practice, the effective number of tests is smaller than the original scan. (We could determine a valid threshold for the max-min scan from the simulation approach with the estimate $\hat{\theta}$.)

Next, when there is a significant result, we investigated how many significant results there are. Since the IBD rate process has non-negligible correlations, we anticipated that multiple significant results would be adjacent to each other. Across non-overlapping windows of varying sizes, we counted the number of windows that had a significant result. Figure S4 shows that the number of windows with a significant result decreases to a median of 1 when the window size is ≥ 0.20 cM and the family-wise significance level is ≤ 0.05. Altogether, we tend to find only one or a few marginally significant results in aggregated regions less than 0.5 cM when a type 1 error is made.

At the 0.05 family-wise significance level, the average standard deviations above the mean using the discrete-spacing analytical approximation are 4.00 and 4.35 for the ≥ 2.0 and ≥ 3.0 cM IBD rate processes, respectively, in the staged exponential growth scenario. The average genome-wide significance levels are 5.41e−6 and 6.82e−6, and the FWERs are 0.148 and 0.036. For the population of a constant size of 50,000 diploid individuals, the average quantiles using the analytical approximation are 4.36 and 4.31 for the ≥ 2.0 and ≥ 3.0 cM IBD rate processes, respectively. The average genome-wide significant levels are 6.56e−6 and 8.38e−6, and the FWERs are 0.114 and 0.034. Regardless of the demographic scenario, the ≥ 2.0 and ≥ 3.0 cM scans may have anti-conservative and conservative control of the FWER, respectively.

Statistical power in selective sweeps

Figures 2 and S6A show the power estimates for the ≥ 2.0 cM IBD rate scan in the population bottleneck, staged exponential growth, and constant population size scenarios with selection coefficients $0.006 \leq s \leq 0.014$ and current-day allele frequencies $0.10 \leq p(0) \leq 0.90$. For the population bottleneck simulations with $0.25 \leq p(0) \leq 0.75$, the power estimates are less than 5% when $s \leq 0.008$ but are greater than 90% when $s \geq 0.014$. In between these extremes, power estimates range from 15% to 40% when $s = 0.010$ and from 55% to 85% when $s = 0.012$. For constant population size

simulations with $0.25 \leq p(0) \leq 0.75$, power estimates are between 0% and 10% when $s \leq 0.012$ but as high as 40% when $s = 0.014$ and $p(0) = 0.50$. Depending on s and $0.25 \leq p(0) \leq 0.75$, the power estimates are 10%–30% higher in the staged exponential growth simulations than they are in the population bottleneck simulations.

Power estimates are uniformly greater with the current-day allele frequency $p(0) = 0.50$ as opposed to $p(0) \in \{0.10, 0.25, 0.75, 0.90\}$. When $s \leq 0.012$ and $p(0) = 0.10$ or $p(0) = 0.90$, power estimates are mostly less than 0.10. The increased ability to detect positive selection when the sweep is at an intermediate present-day frequency is consistent with the analyses in Temple et al.[14]

Across all experiments, we observed power increases over the Bonferroni method as high as 10%; crucially, the Bonferroni significance level depends on our *ad hoc* choice of test spacing, whereas our method adapts to genomic correlations. The parameter boundaries $s \leq 0.01$ and $s > 0.01$ mark a transition consistent across all our demographic scenarios when the $\geq$ 2.0 cM scan has some nonzero statistical power. Temple et al.[14] estimated selection coefficients for sweeps in the TOPMed EUR1 group that exceed 0.015, which our test has high power to detect.

Figures S5 and S6B show the power estimates for the $\geq$ 3.0 cM scan in the population bottleneck, staged exponential growth, and constant population size scenarios. In the population bottleneck and constant population size simulations, we measure zero power for all combinations of selection coefficients and allele frequencies. In the staged exponential growth simulations, we measure power between 10% and 50% for selection coefficients $s > 0.01$ and zero for selection coefficients $s \leq 0.008$. Regardless of demography, rejecting the null model in the $\geq$ 3.0 cM scan could be evidence of an exceptionally strong sweep.

Multiple-testing corrections for human ancestry groups

We modified the Temple et al.[14] workflow to incorporate the analytical approximation and simulation-based approaches for multiple testing. We also provide genome-wide significance levels under the null model that IBD rates are normally distributed. (IBD rates are asymptotically normally distributed under some conditions on large sample size and population size.[61])

For each sample set (summarized in Table 2), we computed IBD rates every 0.02 cM for IBD segments $\geq$ 2.0 and $\geq$ 3.0 cM. Figure S7 indicates that the empirical distributions of IBD rates around a locus resemble normal distributions in our sample sets. The positively skewed IBD rates in the African ancestry samples could be due to population substructure or other unexplained genomic variation.

Figure S8 shows the estimated autocovariances and fitted exponential curves for all our ancestry and ethnicity groups. Upon visual inspection, the fitted exponential curves match the chromosome-specific autocovariances well in the plots for the European ancestry and UKBB Indian British sample sets. In the TOPMed AFR ancestry and UKBB Black British groups, the fitted exponential curves fit the long-range autocovariances well but not the short-range autocovariances.

For IBD segments $\geq$ 2.0 cM, the exponential decay parameter estimates $\widehat{\theta}$ are 45, 30, 50, 49, 83, and 78 for the TOPMed EUR1 ancestry, TOPMed EUR2 ancestry, UKBB white British 410k, UKBB Indian British, TOPMed AFR ancestry, and UKBB Black British groups, respectively. The corresponding discrete-spacing analytical thresholds are IBD rates 1.94e−4, 5.89e−3, 2.66e−4, 1.82e−4, 2.64e−4, and 3.55e−4, and the corresponding genome-wide significance levels are 2.27e−6, 3.27e−6, 2.13e−6, 2.16e−6, 1.36e−6, and 1.46e−6. For each of these estimates of the exponential decay parameter, the discrete-spacing analytical and simulation-based approaches should provide similar genome-wide significance levels (Figure S2).

For IBD segments $\geq$ 3.0 cM, the exponential decay parameter estimates $\widehat{\theta}$ are 33, 36, 39, 53, and 45 for the TOPMed EUR1 ancestry, UKBB white British 410k, UKBB Indian British, TOPMed AFR ancestry, and UKBB Black British groups, respectively. The corresponding discrete-spacing analytical thresholds are IBD rates 4.49e−5, 8.08e−5, 9.31e−5, 6.10e−5, and 8.07e−5, and the corresponding genome-wide significance levels are 3.05e−6, 2.87e−6, 2.70e−6, 2.02e−6, and 2.35e−6.

Selection scans for human ancestry groups

Figure 3 shows the $\geq$ 2.0 cM IBD rates along the autosomes, the autosome-wide median, the heuristic four standard deviations above the median threshold, and the multiple-testing adjusted thresholds for the TOPMed EUR1 ancestry, UKBB White British, UKBB Indian British, and TOPMed EUR2 ancestry groups. Figure 4 shows the $\geq$ 2.0 cM IBD rates along the autosomes, the autosome-wide median, the heuristic four standard deviations above the median threshold, and the multiple-testing adjusted thresholds for the TOPMed AFR ancestry and UKBB Black British groups.

In Tables 3 and 4, we report loci where IBD rates exceed the genome-wide significance threshold for a contiguous stretch of 0.50 cM. We annotated loci with genes or gene complexes if they have been previously reported in the literature, are shared across analyses, or contain only a couple of genes. We caution that the annotated genes are not necessarily causally driving the excess IBD sharing. We calculated p values under the null model for the position in a region with the highest IBD rate. Some of the genome-wide significant signals were replicated by Temple et al.[86] in a selection scan of genetically similar European and African ancestry cohorts, including three of the signals in African ancestry cohorts.

Sequencing and alignment difficulties could affect downstream IBD segment detection and thereby violate

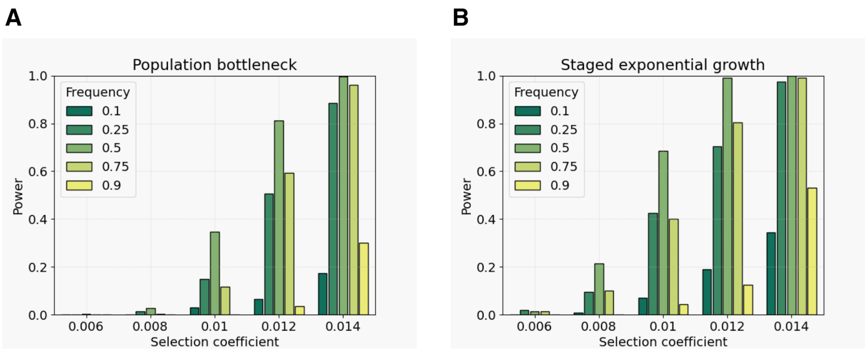

Figure 2. Power simulations for different selection coefficients and current-day sweeping allele frequencies

Bar plots show statistical power (y axis) using true IBD segments $\geq$ 2.0 cM overlapping the selected allele in the (A) population bottleneck and (B) staged exponential growth demographic scenarios. Hypothesis testing is based on the discrete-spacing analytical threshold with a step size of 0.02 cM. Power is the proportion of tests where the null model is rejected at the p value threshold corresponding to the 0.05 family-wise significance level. There are 200 simulations for each pair of selection coefficient (x axis) and current-day allele frequency (colors in legend).

the scan's null model assumptions. Four loci on the chromosome bands 16q12.3, 22q11.21, 15q13.3, and 17q12 that are significant in multiple cohorts also lie in highly variable regions where the Genome Reference Consortium has added alternate locus sequences (Tables 3 and 4). Figures S10–S12 focus on genome-wide significant loci where there are sizable ENCODE blacklist regions,[87] USCS unusual regions, or low mappability in the Genome in a Bottle study.[88] `ibd-ends` tends to underestimate the IBD rates in low-mappability regions as opposed to inflating significance.[24] In Appendix B, we show that the distribution of IBD rates flanking low-mappability regions has more weight for low IBD rates than that of the genome-wide distribution. Lastly, some centromeric regions (e.g., chromosomes 1 and 9) have extremely low IBD rates for all groups (Figures 3 and 4) because length-based IBD detection is difficult in these cases.[24]

Using the original Temple et al.[14] selection scan workflow, twenty-four loci exceed the heuristic threshold of four standard deviations above the autosome-wide median in the $\geq$ 2.0 cM scan for the TOPMed AFR ancestry data.[62] Using our modified workflow with the multiple-testing corrections, we find that only four of these twenty-four loci are genome-wide significant. Similarly, nineteen loci exceed our heuristic threshold of four standard deviations above the autosome-wide median in the $\geq$ 2.0 cM scan for the UKBB Black British data,[62] only ten of which exceed our multiple-testing adjusted threshold.

Except for a 0.02 cM stretch of excess IBD rates in the *MHC* region, no loci are genome-wide significant in the TOPMed EUR2 ancestry data. The mean IBD rate is an order of magnitude larger for this group than for any other group. Recall that this European ancestry sample set is likely made up of descendants from a small founder population. In such a demographic scenario, *de novo* sweeping alleles are more likely to be lost than in large populations.

Figure S9 shows the $\geq$ 3.0 cM IBD rates along the autosomes, the autosome-wide median, the heuristic four standard deviations above the median threshold, and the multiple-testing adjusted thresholds for the TOPMed EUR1, UKBB White British, UKBB Indian British, TOPMed AFR ancestry, and UKBB Black British groups. We report the statistically significant results of the $\geq$ 3.0 cM scan in Table S2.

As a contrast, we studied further significant loci on chromosome bands 7q21.11, 11p15.4, and 16q12.3 that were replicated in our African ancestry analyses versus *LCT* and *OCA2* (MIM: 203200) (believed to be hard sweeps[37]) and *MHC* (some form of balancing selection[23] or multiple sweeps[37]) in our European ancestry analyses. We applied the Temple et al.[14] suite of methods to fine-map candidate alleles for positive selection. One of the Temple et al.[14] methods searches for unusually large clusters sharing an IBD haplotype at the locus of interest and identifies alleles that are predominantly found in the outlier clusters. Table S3 reports the number and proportions of IBD

Table 2. Metadata for analysis cohorts

Name	Ancestry reference	Geographic location	Sample size	Sequencing type	Assembly
TOPMed AFR	African	US	1,737	WGS	GRCh38
UKBB Black British	African	United Kingdom	3,146	SNP array	GRCh37
TOPMed EUR1	European	US	13,778	WGS	GRCh38
TOPMed EUR2	European	US	1,719	WGS	GRCh38
UKBB White British	European	United Kingdom	408,891	SNP array	GRCh37
UKBB Indian British	South Asian	United Kingdom	5,374	SNP array	GRCh37

The cohorts come from the Trans-Omics for Precision Medicine (TOPMed) and United Kingdom British Biobank (UKBB) consortia data. Geographic location refers to where the data were collected. We use the Bhérer et al.[85] and the deCODE 2019[83] genetics maps for the GRCh37 and GRCh38 assemblies, respectively. WGS, whole-genome sequencing.

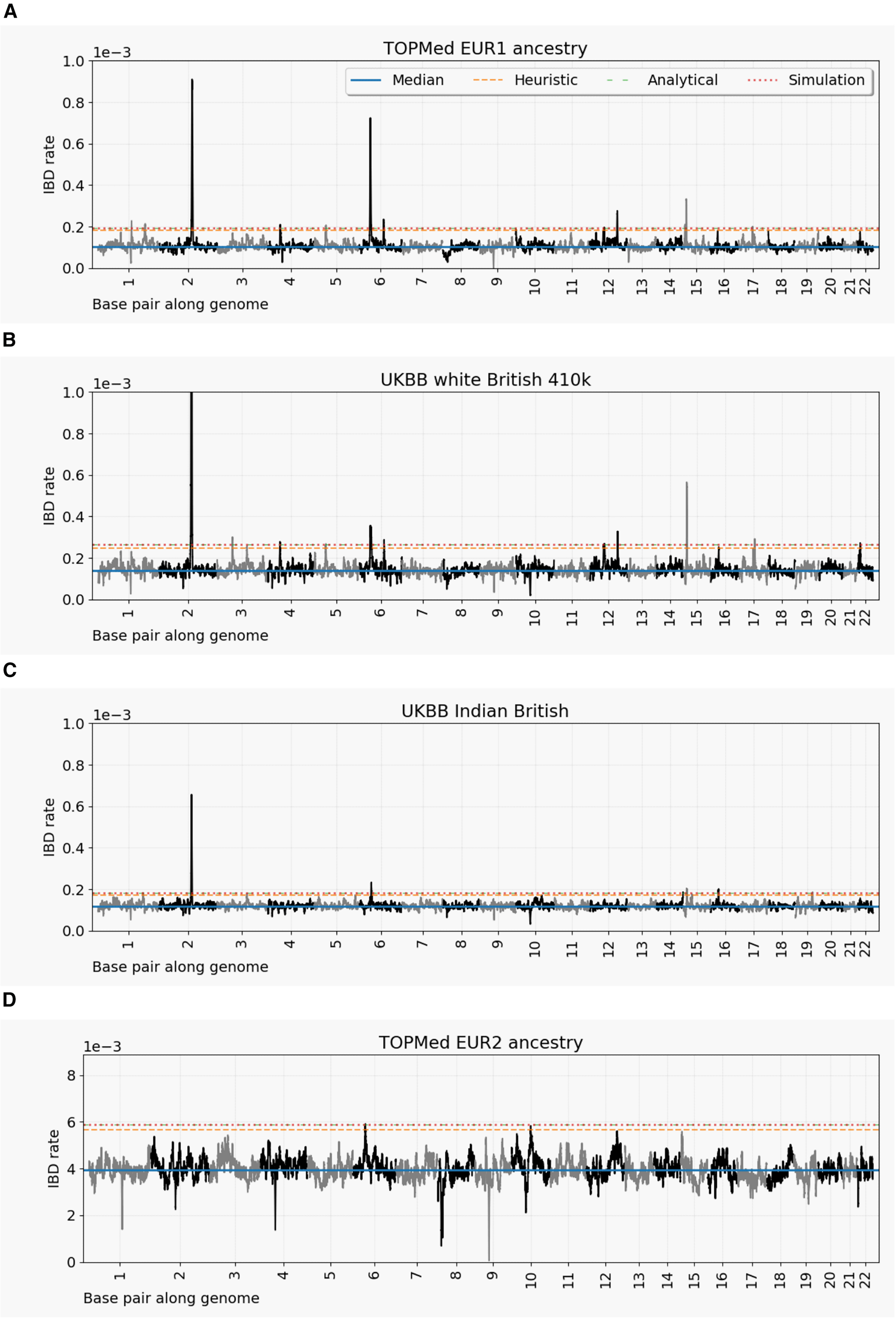

Figure 3. Genome-wide IBD rate scans in European ancestry and Indian British samples
(A–D) Line plots show IBD rates (y axis) every 0.02 cM along the twenty-two human autosomes. The dataset analyzed is given in the subplot titles. Horizontal lines show (blue) the genome-wide median IBD rate, (orange) the heuristic threshold of four standard deviations above the median IBD rate, (green) the discrete-spacing analytical threshold, and (red) the simulation-based threshold. The analytical and simulation-based thresholds are less than 5e−6 apart.

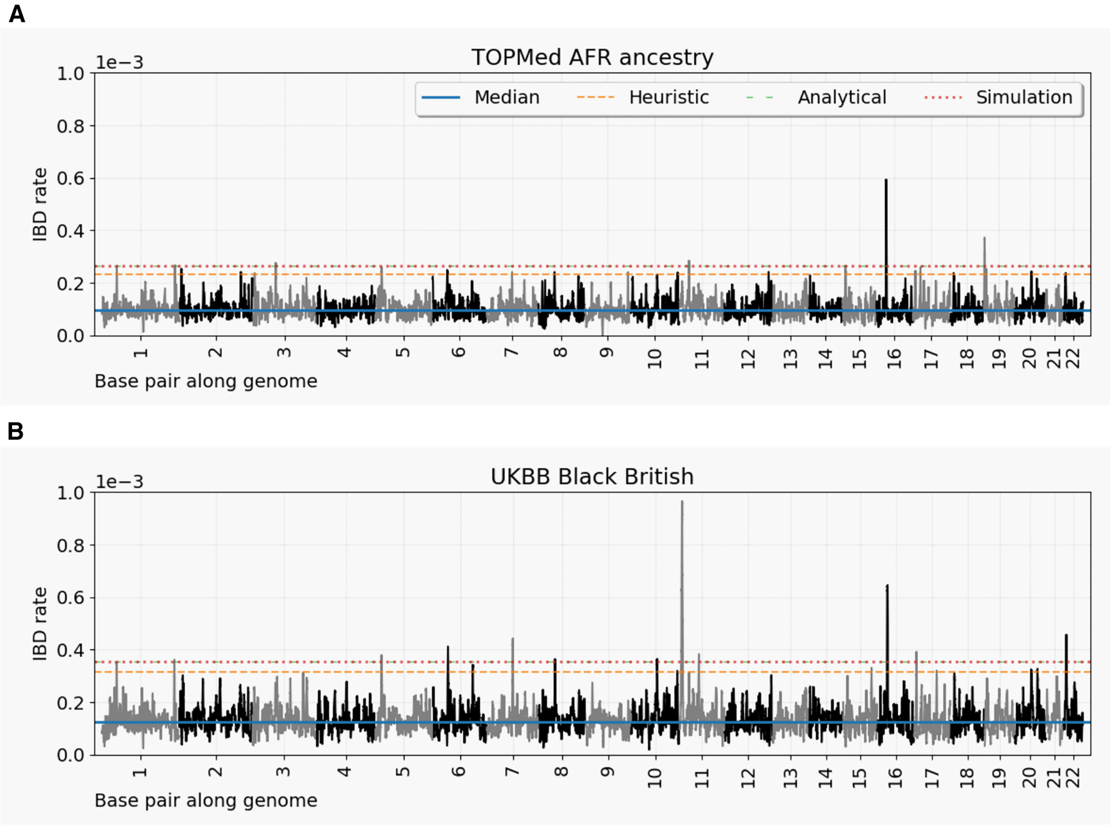

Figure 4. Genome-wide IBD rate scans in African ancestry and Black British samples
(A and B) Line plots show IBD rates (*y* axis) every 0.02 cM along the twenty-two human autosomes. The dataset analyzed is given in the subplot titles. Horizontal lines show (blue) the genome-wide median IBD rate, (orange) the heuristic threshold of four standard deviations above the median IBD rate, (green) the discrete-spacing analytical threshold, and (red) the simulation-based threshold. The analytical and simulation-based thresholds are less than 5e−6 apart.

clusters at the chosen subset of loci in the African ancestry samples versus those in the European ancestry samples. Having a few to one IBD cluster comprising more than 20% of samples is strong evidence of a recent sweep.[14] We observe this phenomenon at *LCT* and *OCA2* in the European ancestry samples—two loci broadly believed to be under selection[37,58]—but not in the significant loci chosen for the African ancestry samples (Table S3).

Replicating selection signals in European ancestry groups

We previously reported eight of the eleven statistically significant loci in the TOPMed EUR1 selection scan.[14] For instance, Figure S13A shows that the maximum IBD rates on chromosome band 12q24.13 are squarely on *OAS1*, *OAS2*, and *OAS3* (MIM: 164350, 603350, and 603351), whose signal may be due to adaptive introgression.[3] There are high IBD rates around the nearby pigmentation genes *TRPM1* (MIM: 603576) and *OCA2* (Figure S10A), but there are also large gaps of detected IBD segments as a result of low marker density and low mappability.[14,87,88] Putatively selected variants around *TRPM1*[14] are not GWAS signals in a multiethnic study of more than 7,000 phenotypes.[89] Structural variation could be another explanation for this signal, as *TRPM1* lies on a chromosome band (15q13.3) containing recurrent deletions,[90] which could increase IBD rates by selection disfavoring the recombination of haplotypes with long deleted tracts. While the region around *OCA2* has low mappability and presents challenges in sequencing, the rapid frequency changes of some *OCA2* variants in ancient DNA provide strong evidence of positive selection.[37] Finally, the *p* value for *LCT* is so small that it cannot be represented in the 64-bit floating-point system.

The three loci not reported in our prior analysis of the TOPMed EUR1 ancestry data have been reported in other studies to be under selection. *TLR1*, *TLR6*, and *TLR10* (MIM: 601194, 605403, and 606270) encode Toll-like receptors that help initiate an immune response and may have been under selection in ancient Eurasians.[38] The high IBD rates on chromosome band 4p14 are centered on *TLR1-6-10* (Figure S14A). Gittelman et al.[91] have suggested that an introgressed Neanderthal haplotype covering *TLR1-6-10* may have been under selection. Multiple late cornified envelope (*LCE*) genes in the human epidermal complex are a few tens of kb from the significant locus on chromosome band 1q21.3 and are highly expressed in skin. The high IBD rates on chromosome band 17q12 are centered about *HNF1B* (MIM: 189907) (Figure S11A), which includes rare deletions[90] and is associated with diabetes and prostate cancer.[92,93]

Table 3. Loci detected in European ancestry and UKBB Indian British selection scans

Dataset	Chr	Rate (1e−4)	Region size (cM)	Position (Mb)	Genes	*p* value
TOPMed EUR1 (GRCh38)	2	9.10	7.38	134.84 (132.52–139.90)	*LCT*	<5e−324
TOPMed EUR1 (GRCh38)	6	7.24	6.94	30.80 (24.10–36.13)	*MHC* [a]	8.18e−222
TOPMed EUR1 (GRCh38)	15	3.33	2.44	31.18 (30.34–32.16)	*TRPM1*[a]	5.50e−32
TOPMed EUR1 (GRCh38)	12	2.75	2.60	113.08 (110.89–113.65)	*OAS1-2-3*	1.50e−18
TOPMed EUR1 (GRCh38)	6	2.35	1.86	105.98 (105.76–106.47)	*PRDM1*	1.35e−11
TOPMed EUR1 (GRCh38)	1	2.28	0.82	152.47 (151.48–152.56)	*LCE*	1.64e−10
TOPMed EUR1 (GRCh38)	1	2.14	2.40	206.62 (205.49–207.02)	·	1.32e−8
TOPMed EUR1 (GRCh38)	15	2.10	1.18	28.09 (27.93–28.85)	*OCA2*[a]	3.40e−8
TOPMed EUR1 (GRCh38)	4	2.09	1.50	38.75 (38.28–38.97)	*TLR1-6-10*	4.24e−8
TOPMed EUR1 (GRCh38)	5	2.06	1.62	33.96 (32.99–33.99)	*SLC45A2*	1.13e−7
TOPMed EUR1 (GRCh38)	17	2.00	0.64	37.63 (37.66–37.74)	*HNF1B*[a]	5.55e−7
UKBB White British 410k (GRCh37)	2	16.69	7.96	135.87 (133.23–140.91)	*LCT*	<5e−324
UKBB White British 410k (GRCh37)	15	5.65	2.88	31.47 (30.36–32.64)	*TRPM1*[a]	7.31e−56
UKBB White British 410k (GRCh37)	6	3.56	6.70	25.44 (24.14–35.71)	*MHC*[a]	1.17e−15
UKBB White British 410k (GRCh37)	12	3.27	2.18	113.41 (111.63–114.05)	*OAS1-2-3*	3.25e−12
UKBB White British 410k (GRCh37)	3	3.01	1.64	47.59 (45.82–51.94)	*CCR9*	2.05e−9
UKBB White British 410k (GRCh37)	17	2.92	1.10	44.62 (42.87–44.92)	*MAPT*[a]	1.53e−8
UKBB White British 410k (GRCh37)	4	2.78	0.86	38.81 (38.57–38.98)	*TLR1-6-10*	2.51e−7
UKBB Indian British (GRCh37)	2	6.62	5.62	136.98 (134.23–139.81)	*LCT*	<5e−324
UKBB Indian British (GRCh37)	6	2.40	3.24	33.92 (32.97–36.34)	*MHC*[a]	2.34e−17
UKBB Indian British (GRCh37)	15	2.09	2.56	31.48 (30.80–32.51)	*TRPM1*[a]	2.84e−10
UKBB Indian British (GRCh37)	16	2.05	2.60	17.83 (16.93–18.26)	*XYLT1*[a]	7.43e−10
UKBB Indian British (GRCh37)	19	1.86	0.60	50.30 (50.23–50.45)	·	4.42e−7

We report loci where identity-by-descent (IBD) rates exceed the multiple-testing analytical thresholds of 1.94e−4, 2.66e−4, and 1.82e−4 for the TOPMed EUR1 ancestry, UKBB White British, and UKBB Indian British sample sets, respectively. The maximum IBD rate is given for each locus. Physical positions for the location of the maximum IBD rate and the span of excess IBD rates are shown in megabases (Mb). We report the size in centimorgan (cM) of each region, which is defined to be a contiguous stretch of IBD rates exceeding the genome-wide significance threshold. Pedigree-based recombination maps from Halldorsson et al.[83] and Bhérer et al.[85] aligned to the GRCh38 and GRCh37 reference genomes are used for inferring IBD segments in the TOPMed and UKBB sample sets, respectively. *p* values are calculated assuming the null model that IBD rates are normally distributed. Annotated genes or gene complexes are discussed in the main text and have also previously been reported in the literature. The cells that contain dots are signals that are not discussed with respect to specific genes or gene complexes. The IBD segment detection threshold is 2.0 cM.

[a]These signals overlap hotspots of recurrent copy-number variation and/or have multiple alternate locus sequences that have been added by the Genome Reference Consortium.

Based on our simulation study of statistical power, we expect that hard sweeps from a single beneficial allele that are detected in the $\geq$ 3.0 cM scan will also be detected in the $\geq$ 2.0 cM scan. In the TOPMed EUR1 ancestry data, four significant loci in the $\geq$ 3.0 cM scan are also significant loci in the $\geq$ 2.0 cM scan. The signal near *HNF1B* is barely genome-wide significant in the $\geq$ 2.0 cM scan but is the third most significant in the $\geq$ 3.0 cM scan (Table S2; Figure S11C). There are also flanking regions that have low mappability for hundreds of kb,[87,88] raising the possibility that this 1.5-Mb-long signal is a technical artifact. At the same time, Browning and Browning[24] show that low mappability tends to decrease IBD rates, and it is unclear under what circumstances low mappability would increase IBD rates. The three loci significant in the $\geq$ 3.0 cM scan but not in the $\geq$ 2.0 cM scan include a family of keratin genes on chromosome 12 (*KRT*), a few hundred kb upstream of the immunoglobulin lambda genes (*IGL*), and a gene-sparse region on chromosome band 16q12.3 (Figure S12F).

In the UKBB White British data, we observed $\geq$ 2.0 and $\geq$ 3.0 cM IBD rates exceeding our genome-wide significance threshold at many of the same loci significant in the TOPMed EUR1 ancestry analysis (Tables 3 and S2), including the putative examples of adaptive introgression at *OAS1-2-3* and *TLR1-6-10* (Figures S13B and S14B). Fewer IBD segments were detected around and flanking *OCA2* (Figure S10B), which is a low-mappability and low-marker-density region.[87,88] Five of the twelve primary selection signals and none of the secondary selection signals in the Browning and Browning[24] analysis of the UKBB White British data are genome-wide significant in our scan.

Table 4. Loci detected in African ancestry selection scans

Dataset	Chr	Rate (1e−4)	Region size (cM)	Position (Mb)	Genes	*p* value
TOPMed AFR (GRCh38)	16	5.93	2.94	17.01 (16.53–18.24)	*XYLT1*[a]	5.94e−46
TOPMed AFR (GRCh38)	19	3.72	2.08	1.74 (1.67–2.01)	·	2.78e−15
TOPMed AFR (GRCh38)	11	2.85	0.92	19.94 (19.88–20.00)	·	6.05e−8
TOPMed AFR (GRCh38)	3	2.77	1.02	60.59 (60.49–60.76)	·	2.12e−7
UKBB Black British (GRCh37)	11	9.66	5.12	5.22 (3.32–6.35)	*HBB*[b]	5.57e−69
UKBB Black British (GRCh37)	16	6.46	2.94	17.76 (16.81–18.55)	*XYLT1*[a]	1.95e−27
UKBB Black British (GRCh37)	22	4.57	2.06	21.41 (20.96–22.03)	·	4.64e−12
UKBB Black British (GRCh37)	7	4.43	1.80	80.35 (79.89–80.62)	*SEMA3C*	3.55e−11
UKBB Black British (GRCh37)	6	4.12	1.26	34.41 (31.92–37.71)	*MHC*[a,b]	2.27e−9
UKBB Black British (GRCh37)	17	3.92	0.96	3.69 (3.64–3.80)	·	2.54e−8
UKBB Black British (GRCh37)	11	3.83	0.84	61.29 (60.84–61.62)	·	7.15e−8
UKBB Black British (GRCh37)	5	3.79	1.16	9.62 (9.45–9.88)	*TAS2R1*	1.11e−7
UKBB Black British (GRCh37)	10	3.64	0.50	79.47 (79.21–79.49)	·	5.54e−7
UKBB Black British (GRCh37)	8	3.64	0.58	37.18 (37.12–37.47)	·	6.00e−7

We report loci where identity-by-descent (IBD) rates exceed the multiple-testing analytical thresholds of 2.63e−4 and 3.55e−4 for the TOPMed AFR ancestry and UKBB Black British sample sets, respectively. The maximum IBD rate is given for each locus. Physical positions for the location of the maximum IBD rate and the span of excess IBD rates are shown in megabases (Mb). We report the size in centimorgan (cM) of each region, which is defined to be a contiguous stretch of IBD rates exceeding the genome-wide significance threshold. Pedigree-based recombination maps from Halldorsson et al.[83] and Bhérer et al.[85] aligned to the GRCh38 and GRCh37 reference genomes are used for inferring IBD segments in the TOPMed and UKBB sample sets, respectively. *p* values are calculated assuming the null model that IBD rates are normally distributed. Annotated genes or gene complexes are discussed in the main text. The cells that contain dots indicate signals that are not discussed with respect to specific genes or gene complexes. The IBD segment detection threshold is 2.0 cM.

[a]These signals overlap hotspots of recurrent copy-number variation and/or have multiple alternate locus sequences that have been added by the Genome Reference Consortium.

[b]These signals have previously been reported in the literature.

Two loci are genome-wide significant in the UKBB White British scan but not in the TOPMed EUR1 ancestry scan. *CCR9* (MIM: 604738) encodes a chemokine receptor that plays an essential role in the mucosal immune system[94] and has been associated with increased COVID-19 outcome severity, especially in Europeans.[95] At this locus, Browning et al.[96] and Ding et al.[97] have suggested that introgressed Neanderthal haplotypes may be selected for in South and East Asians, respectively. *MAPT* (MIM: 157140) on chromosome band 17q21.31 is contained within a 900 kb polymorphic inversion (Figure S11B) that may have been subject to recent selection in European ancestry populations.[98] IBD rates are high around *MAPT* in the TOPMed EUR1 samples as well but at least one standard deviation removed from genome-wide significance (Figure S11A). Genotyping errors at this chromosomal inversion could result in false positive or false negative signals in functional genomics studies,[87,88] but it is unclear how they could increase the IBD rate. Another possibility is that recurrent deletions in adjacent *KANSL1* (MIM: 612452) reduce recombination.[90]

Shared selection signals across ancestry groups

In the UKBB Indian British data, we also observed excess $\geq$ 2.0 and $\geq$ 3.0 cM IBD rates at *LCT*, *MHC*, and *TRPM1* regions (Tables 3 and 4). Romero et al.[99] have suggested that northern European haplotypes carrying a putatively selected allele at *LCT* may be identical by descent to haplotypes in Indian pastoralists. Using the methods in Temple et al.,[14] we inferred an excess IBD outgroup comprising 17% of the samples (Table S3), which would be in the range of the selected allele frequency in Indian pastoralists in Romero et al.[99] The rates of IBD alleles near the human leukocyte antigen (*HLA*) genes are known to be high in all HapMap populations,[23] which is consistent with our selection scan results near the *HLA* genes. Excess IBD rates in the UKBB Indian British samples only overlap two of the three *HLA* regions reported to be under selection by Mathieson and Terhorst.[37] In contrast, excess IBD rates in the European ancestry samples overlap all three selected loci. Browning et al.[96] previously reported evidence of archaic selection around *CCR9* in a South Asian ancestry group, but we did not observe a genome-wide significant signal of recent selection in our UKBB Indian British scan. *TRPM1* is a couple of Mb away from *OCA2*, which has geographic patterns of population genetic variation indicative of strong selection.[100]

In the $\geq$ 2.0 cM scan for the UKBB Black British group and in the $\geq$ 3.0 cM scan for the TOPMed EUR1, UKBB White British, TOPMed AFR ancestry, and UKBB Black British groups, we observed a genome-wide significant locus on chromosome band 22q11.21. Contiguous stretches of excess IBD rates span between 2.06 and 5.56 cM in the

different analyses, which is larger than many of the other genome-wide significant regions (Tables 3, 4, and S2). The locations of maximum IBD rates are at roughly 21.50 and 20.25 Mb between analyses using GRCh37 versus GRCh38 reference builds, which do not map to the same sets of genes. In their analysis of the UKBB White British data, Browning and Browning[24] reported that the selection signal is close to *UBE2L3* (MIM: 603721), which is associated with multiple autoimmune diseases.[101] The *IGL* genes involved in the adaptive immune system are also a few hundred kb downstream of this region. The chromosomal band 22q11.21 also has a cluster of low-copy repeats that mediate non-allelic homologous recombination, leading to recurrent copy-number variants (CNVs).[90,102] The most common CNV is a roughly 3 Mb deletion that causes DiGeorge syndrome and is estimated to have a prevalence of 1 in 4,000 births.[102] Overall, there is no clear indication across analyses of which genes within this gene-dense region could explain this signal.

IBD rates spanning a couple of Mb on chromosome band 16p12.3 are genome-wide significant in the $\geq$ 2.0 cM scans for UKBB Indian British, TOPMed AFR ancestry, and UKBB Black British groups and in the $\geq$ 3.0 cM scans for TOPMed EUR1 ancestry and UKBB White British groups (Tables 3, 4, and S2; Figures S9 and S12). This region's most extreme $\geq$ 2.0 cM IBD rates are 14.17 and 10.78 standard deviations above the autosome-wide means in the TOPMed AFR ancestry and UKBB Black British groups (Figure 4). This region's maximum $\geq$ 2.0 cM IBD rate is only 6.05 standard deviations above the autosome-wide mean in the UKBB Indian British data. (For reference, the IBD rate at *TRPM1* is 11.71 standard deviations above the autosome-wide mean in the TOPMed EUR1 ancestry group.) Excess IBD rates span at least 2.5 cM of this region in all of these analyses. Applying the subgroup anomaly detection method in Temple et al.[14] to the TOPMed AFR ancestry data, we failed to detect a singular excess IBD-sharing cluster at this locus (Table S3), which would have been indicative of a hard selective sweep.

This 1.5 Mb genomic region contains few genes, with the excess IBD rates entirely spanning the more than 300 kb *XYLT1* (MIM: 608124). There are regions hundreds of kb long around *XYLT1* with unresolved conflicting genotypes,[88] but the IBD rates decrease along those flanking regions (Figure S12). *XYLT1* encodes the xylosyltransferase 1 enzyme, which initiates a chain reaction in the early maturation of skeletal cells, and a couple recessive missense mutations in *XYLT1* are connected to dwarfism.[103,104] *XYLT1* is just downstream of a genomic hotspot for deletions (chromosomal band 16q13.11), with some deletions spanning more than 3 Mb and many variants associated with neurodevelopmental diseases.[90,105]

African ancestry-specific recent selection signals

Some genome-wide significant loci are only found in the African ancestry analyses. For example, excess IBD rates also cover most of *SEMA5A* (MIM: 609297) and *TAS2R1* (MIM: 604796) on chromosome bands 5p15.31–5p15.2 (Figure S15). *SEMA5A* encodes a protein specifically expressed around retinal axons in the optic nerve and helps maintain the axons' structural integrity.[106] *TAS2R* genes mediate bitter taste perception, and their high rates of amino acid substitutions and diversity between human populations could be due to selection.[107]

Around the genome-wide significant signal on chromosome band 7q21.11 in the UKBB Black British selection scans, we observed a subset of SNPs (GRCh37, chr7:80390598, 80624286, and 80715067) strongly differentiated between a small group of excess IBD sharing (Table S3) and the rest of the sample.[14] These SNPs have frequencies between 72% and 79%, 15% and 20%, and 20% and 25% in the excess IBD-sharing group, the rest of the sample, and the entire sample, respectively. The SNPs lie in *SEMA3C* (MIM: 602645). This gene encodes a protein involved in neuronal guidance. Expression of this gene is positively correlated with Wnt pathway activation, which is often dysregulated in brain tumor cancers.[108]

We observed a genome-wide significant locus on chromosome band 11p15.4 in the $\geq$ 3.0 cM scan for TOPMed AFR ancestry samples and in the $\geq$ 2.0 and $\geq$ 3.0 cM scans for the UKBB Black British samples (Figure S16). This locus has more extreme IBD rates than *XYLT1* in the UKBB Black British data. At this locus, we applied the Temple et al.[14] methods to the UKBB Black British data to detect a subset of SNPs strongly differentiated between a group of excess IBD sharing and the rest of the sample. We observed various well-differentiated SNPs (GRCh37, chr11:5221233, 5223750, and 5214301) within tens of kb of *HBB* (MIM: 141900). These SNPs have frequencies between 81% and 85%, 14% and 19%, and 22% and 27% in the excess IBD-sharing group, the rest of the sample, and the entire sample, respectively. Hemoglobins are proteins in red blood cells that transport oxygen to cells and tissues.[109] Mutations in the cluster of genes encoding the hemoglobin beta subunits are suspected to be targets of selection to reduce susceptibility to infections and malaria but also cause sickle cell anemia and beta thalassemia disorders.[110]

Discussion

In this paper, we modeled the correlation of detectable IBD segments along chromosomes to determine approximate genome-wide significance levels for an IBD rate-based selection scan. One of our approaches calculates the genome-wide significance level analytically, compared to permutation- and simulation-based approaches that are common in genetic studies but can be computationally intensive or intractable. Developing valid multiple-testing approaches is important for complex haplotype-based analyses instead of using the

GWAS significance level of 5e−8, lest we inflate type 1 errors or decrease the power to reject false null models. By properly accounting for correlations between test statistics, we can perform hypothesis tests finely spaced along the autosomes, thereby increasing statistical power.

Due to the speed of the msprime and tskibd methods for simulating IBD segments along entire chromosomes, we could measure the FWER in different demographic scenarios and under various experimental conditions. Many methods to detect recent selection have not measured the FWER in simulation studies, in large part because of the immense computation that would be involved, nor have they proposed multiple-testing corrections.[17,20,26,30–35] We find that our $\geq$ 2.0 and $\geq$ 3.0 cM scans have slightly anti-conservative and conservative control of the FWER, respectively. The asymptotic conditions of Temple and Thompson[61] are less valid in the $\geq$ 2.0 cM scan, which may explain its anti-conservative behavior. The asymptotic conditions of Temple and Thompson[61] are more reasonable in the $\geq$ 3.0 cM scan, but the Siegmund and Yakir[56] analytical approximation is conservative for true OU processes.

Unless the genetic data have low coverage or poor genotyping quality such that detecting IBD segments less than 3.0 cM is inaccurate,[111,112] we recommend using the anti-conservative $\geq$ 2.0 cM scan over the conservative $\geq$ 3.0 cM scan, which has limited power. The $\geq$ 3.0 cM scan has limited power to detect hard sweeps of $s<0.015$, which Schrider and Kern[43] describe as strong selection. On the other hand, we found that the $\geq$ 2.0 cM scan has some power when $s \leq 0.010$ and considerable power when $s > 0.01$. Indeed, the heuristic threshold of Temple et al.[14] corresponds to the expected IBD rate of an $s = 0.017$ sweep in the TOPMed EUR1 ancestry samples. Some methods claim to have the power to detect sweeps where $s<0.010$.[34,35,39,40] However, these methods do not address multiple testing. We suggest that selection coefficients $s <$ 0.01 and $s \geq 0.010$ may describe undetectable and detectable recent sweeps once multiple testing is accounted for.

We considered the hard-sweep model in our power simulations, which is one of many alternative models that could explain excess IBD rates. The pairwise IBD rate test does not resolve the classification of hard and soft sweeps versus recurrent sweeps versus balancing selection versus other mechanisms, which is a topic of growing interest in the field.[40,43,113] We observed that hard sweeps detected in the $\geq$ 3.0 cM scan were almost always detected in the $\geq$ 2.0 cM scan, in which case loci significant in the $\geq$ 3.0 cM scan but not in the $\geq$ 2.0 cM scan may not be the result of a hard sweep. In practice, we should account for the fact that conducting scans with multiple different segment length thresholds is another form of multiple testing (Appendix C). Temple et al.[14] have also proposed various diagnostics as characteristic of a hard sweep, particularly that of a single majority haplotype cluster with excess IBD rates and a reduction in the diversity of common variants, which we did not observe at the African ancestry-specific signals (Table S3).

Failing to adjust for multiple testing properly can be a cause for concern in discovery studies. In our study, we investigated signals of natural selection in human populations, in which significant findings could be misinterpreted or misappropriated.[48] After adjusting for multiple testing, we identified eleven or fewer statistically significant results in any given ancestry or ethnicity cohort. In contrast, Akbari et al.[114] reported more than 300 independent significant results of recent selection using a novel rescaling to address genomic inflation. We have validated control of the FWER in simulation studies, whereas Akbari et al.[114] have not. At the same time, the Akbari et al.[114] method is designed for slightly older selection (~300 generations ago) than our method (~100 generations ago).

The four standard deviations above the autosome-wide median IBD rate is a heuristic threshold used in our previous work.[14] For the TOPMed EUR1 ancestry samples, our new multiple-testing approach results in a nearly identical threshold. However, the four standard deviation threshold is not large enough for studies on other ancestry groups. Indeed, we suggest that the many loci with IBD rates exceeding four standard deviations could be false positives in African ancestry cohorts. Even so, many, but not all, of the genome-wide significant loci in the TOPMed AFR, the UKBB Black British, and an African ancestry cohort analyzed by Temple et al.[86] were not replicated. Population structure could increase IBD sharing within a cohort, consistent with heavier tails in the African ancestry analyses (Figures S7D and S7E), and thereby create false positives despite our adjustment. To maintain an adequate sample size in the TOPMed AFR cohort, there is certainly an unaccounted-for structure consisting of Afro-Caribbeans in Barbados and African Americans in the southwest US (TOPMed).

Scans for haplotype homozygosity are often called selection scans but rather indicate regions of high, long-range, or unusual LD—from whatever evolutionary or molecular mechanisms, structural variation, or bioinformatic effects—and could be important to account for in downstream analyses.[29,64,86] We detected excess IBD rates on the chromosome bands 16p12.3 and 22q11.21 in all ancestry groups. Gusev et al.[115] observed that these regions on the chromosome bands 16p13.11–16p12.3 and 22q.11.21 have significantly elevated IBD sharing within *and between* ancestrally diverse HapMap populations. They did not identify specific genes that could explain their IBD-sharing signal but rather conjectured that the abundance of long haplotypes could be due to reduced inter-haplotype recombination or selection against recombinants.[115] Their work provides supporting evidence to our results that these regions harbor long IBD segments in many ancestry groups. Most of the other signals shared across cohorts, as well as these two aforementioned loci, contain regions with recurrent copy-number or structural variation (Tables 3, 4, and S2). One caveat is that these particular regions are difficult to sequence, but so are *MHC*, *OCA2*, and *MAPT*, which are believed to be under

some form of selection.[23,37,98] Because the overall trend is that IBD rates decrease around low-mappability and otherwise blacklisted/problematic regions (Figures S10–S12 and S19; Appendix B), we think that a (non-sweep) selection mechanism from structural variants is a more plausible explanation for the excess IBD rates on chromosome bands 16q12.3 and 22q11.21 than are technical artifacts like inaccurate IBD segment detection in the presence of CNVs, low mappability, or alternate locus sequences.[90,105]

The hypothesis test and our multiple-testing corrections are so far limited to analyzing the autosomes of samples from large populations with panmixia. Fine-scale population structure exists even within broad-level ancestry groups[71,116] and is often adjusted for in association studies, but our simple scan has no direct way to account for such. Admixture is another form of population structure that can affect the distribution of IBD sharing. We thus evaluated how the upper percentiles of the standardized IBD rates in admixed cohorts compare to those of a Gaussian random variable. We reran the selection scan on chromosomes 13, 14, 17, 18, 20, and 21 (limited evidence of selection) for five (overlapping) subsets of TOPMed AFR samples with a minimum of 76%, 80%, 84%, 88%, and 92% genome-average admixture proportions (YRI reference panel). For the standard normal distribution, the 99.997th percentile is 4.00; in contrast, we calculated the 99.997th percentiles of the standardized IBD rates, which were 4.42, 4.36, 4.49, 5.15, and 5.12 for the minimum 76%, 80%, 84%, 88%, and 92% African ancestry cohorts, respectively. Since the upper percentiles decrease as the cohort becomes less admixed, we argue that admixture is not the primary explanation for inflated IBD rates in the African ancestry cohorts (Figure S7). Future work could compare IBD rates between different ancestry groups, akin to cross-population extended haplotype homozygosity.[17]

Two additional limitations of our selection scan are genome size and sample size. To reliably estimate the autosome-wide mean and standard deviation and the exponential decay parameter, we require more than 400 cM of genetic data. Additionally, the IBD rates along the chromosomes should not be zero, which happens when the sample size is too small to observe IBD segments $\geq$ 2.0 cM. For human genetics studies, using 1,000 samples is likely sufficient to apply our methodology,[14,62] albeit we recommend the analysis of at least a few thousand samples when available. In the case of small samples, one can review the scan plots output from the automated workflow to assess if the IBD rates are zero.

Finally, our modeling assumptions are unreasonable in samples from a small population. When the Temple and Thompson[61] assumption on large effective population size is violated, the upper tail probabilities of high IBD rates can be greater than those of normal distributions. Modeling higher variance processes, like a Lévy-driven OU process,[117] may be necessary to control the FWER of our selection scan when studying samples from founder or domesticated populations.

Replicating genome-wide significant results in different datasets and using different parameter configurations helps validate scientific results. Around many significant loci, we show excess IBD rates in datasets of similar ancestry compositions but with different sequencing technologies. Running our selection scan in other European and African ancestry datasets or in other ancestry groups could corroborate our results and/or existing selection studies, for instance, selection at the *FADS* genes (MIM: 606148, 606149)[38,118] and *EDAR* (MIM: 604095).[34,119]

Analyzing chromosome 2 for the 1,737 whole-genome sequences in the TOPMed African ancestry data took less than half a day with 8 Intel 2.60 GHz CPUs, and analyzing chromosome 2 for 2,500 Indian British samples in the UKBB SNP array data took less than 30 min with 8 Intel 2.60 GHz CPUs. Temple[62] shows that the $\geq$ 2.0 cM selection scan for 2,000 randomly selected samples from the UKBB White British 410k data provides similar results to our analysis of the entire dataset. Compared to GWAS, where using more samples leads to a smaller standard error and thereby more power to detect a nonzero regression effect, our selection scan is a test of neutrality for a stochastic process. Using more samples than necessary can lead to substantial runtime, random access memory (RAM), and disk memory costs: analyzing chromosome 2 for all UKBB White British samples took nearly a week with 16 Intel 2.60 GHz CPUs and 256 GB RAM, and the analysis of all autosomes left a memory footprint of 2.8 TB.

Data and code availability

The multiple-testing corrections for the analytical and simulation-based approaches and the selection scan are implemented as "multiple-testing-analytical.py" and "multiple-testing-simulation.py" under the directories "scripts/scan" and "workflow/scan-selection," respectively, of commit 89800b7 on the main branch (https://github.com/sdtemple/isweep/tree/main). Scripts to conduct the simulation studies are available in the v1.0 tag (https://github.com/sdtemple/isweep/releases/tag/v1.0) under the directory "isweep-1.0/papers/mult-test-paper."

Acknowledgments

This research has received funding from the US National Human Genome Research Institute of the National Institutes of Health under award number HG005701. S.D.T. also acknowledges funding support from the US Department of Defense National Defense Science and Engineering Graduate Fellowship, the US National Institutes of Health T32 GM081062 Predoctoral Training Grant in Statistical Genetics, and The Eric and Wendy Schmidt AI in Science Postdoctoral Fellowship by Schmidt Sciences, LLC. This research has used the UK Biobank Resource under application number 19934. Molecular data for the Trans-Omics in Precision Medicine (TOPMed) program were supported by the National Heart, Lung, and Blood Institute (NHLBI). The content of this article is solely the responsibility of the authors and does not necessarily represent the official views of the National Institutes of Health. Core support, including centralized genomic-read mapping and genotype calling, along with variant quality metrics and filtering, was provided

by the TOPMed Informatics Research Center (3R01HL-117626-02S1; contract HHSN268201800002I). Core support, including phenotype harmonization, data management, sample-identity QC, and general program coordination, was provided by the TOPMed Data Coordinating Center (R01HL-120393; U01HL-120393; contract HHSN268201800001I). See the supplemental information for acknowledgments of individual studies in the TOPMed data. We thank Ruoyi Cai for helpful discussions about the UK Biobank data, Kelsey Grinde for helpful discussions about the OU process, and Elizabeth Thompson, Kelley Harris, and Ryan Waples for feedback on early drafts of this manuscript.

Author contributions

S.D.T. planned the study, wrote the software, conducted the analysis, and wrote the manuscript. S.D.T. and S.R.B. developed the method. S.R.B. proposed the study and contributed to editing the manuscript.

Declaration of interests

The authors declare no competing interests.

Appendix A: Accuracy of IBD segment detection

In a pilot study of ten population bottleneck simulations, we placed mutations on the `msprime` tree sequence at a genome-wide rate of 1e−8. Then, we inferred IBD segments with the `hap-ibd` and `ibd-ends` analysis workflow in Temple et al.[14] Figures S17A and S17B illustrate one simulation of the true `tskibd` and inferred IBD rate processes across the genome. We observed similar genome-wide median IBD rates and significance thresholds between the true and inferred IBD rate processes. The inferred IBD rates are within 95%–105% of the corresponding true IBD rates (Figure S17C). Across the ten simulations, the average estimates of $\widehat{\theta}$ are 69 and 75, and the average standard deviations $\widehat{\sigma}_{1:M}$ are 19 and 20 for the true and inferred IBD rate processes, respectively.

We also conducted the same pilot study for five simulations of the constant size population of 50,000 diploid individuals. Figure S18A illustrates one simulation of the inferred IBD rates divided by the true IBD rates across the genome. The inferred IBD rates are within 90%–95% of the corresponding true IBD rates. We also observed a pattern of higher inferred IBD rates near the chromosome ends than the genome-wide median IBD rate. We ran `ibd-ends` again with the hidden parameter `ne = 50000`, observing only marginal differences compared to the software default setting (Figures S18B and S18C). For this demographic scenario, the differential detection accuracy of `ibd-ends` near chromosome ends could affect the control of the FWER.

Appendix B: Flanking low-mappability regions

We also contrasted genome-wide IBD rates to IBD rates flanking problematic regions (low mappability, segmental duplications, difficult to sequence, etc.) from the Genome in a Bottle (GIAB) project. We subsampled the list of problematic regions to those spanning more than 50 and 100 kb, and we combined consecutive regions if they lay within 25 and 50 kb of each other. (Note that most, but not all, of the problematic regions and their sizes are the same in the two lists as defined. The point we are making is that the following result is robust to different definitions of a large problematic "region.") Then, we computed the IBD rates flanking these regions at intervals of 100 kb up to 1 Mb.

For the TOPMed EUR1 and AFR ancestry groups, Figure S19 shows IBD rates in these flanking regions versus the genome-wide distribution. Overall, IBD rates are not uniformly elevated around GIAB problematic regions, and there are more extremely small IBD rates flanking the GIAB problematic regions compared to the genome-wide distribution. For example, the general trend in the TOPMed EUR1 data is that IBD rates flanking the GIAB problematic regions are smaller than the genome-wide distribution, but the IBD rates containing *OCA2* and *HNFB1*—which flank GIAB problematic regions—are extremes. The general trend in the TOPMed AFR data is that IBD rates flanking the GIAB problematic regions are smaller than the genome-wide distribution, but the IBD rates containing *XYLT1*—which flank GIAB problematic regions—are extremes.

Appendix C: Multiple testing by using different segment length thresholds

A multiple-testing adjustment in a joint ≥ 2.0 and ≥ 3.0 cM scan should not be drastically different from the multiple-testing adjustment in the ≥ 2.0 cM scan because the individual ≥ 2.0 and ≥ 3.0 cM scans are highly correlated. In the population bottleneck simulations, we calculated that the medians of estimates $\widehat{\theta}$ for the ≥ 2.0 and ≥ 3.0 cM IBD rates are roughly 63 and 40. We also calculated that the median of cross-correlations between the ≥ 2.0 and ≥ 3.0 cM (standardized) IBD rates is roughly 0.68. Next, we simulated a two-dimensional standardized OU process 2,000 times with the cross-correlation parameter $\rho = 0.68$ and autocorrelation parameters $\theta_1 = 63$ and $\theta_2 = 40$. The data for each simulation are equivalent to ten chromosomes of length 100 cM and testing every 0.02 cM. From these simulations, we calculated that the 95th percentiles of the maxima of the marginal OU processes with $\theta_1 = 63$ and $\theta_2 = 40$ are 4.36 and 4.24, which correspond to genome-wide significance levels of 6.50e−6 and 1.12e−5. We also calculated that the 95th percentile of the maxima of the two-dimensional OU process is roughly 4.47, corresponding to a significance level of 3.91e−6.

Web resources

Bhérer et al.[85] genetic map, https://github.com/cbherer/Bherer_etal_SexualDimorphismRecombination

deCODE[83] genetic map, https://www.science.org/doi/suppl/10.1126/science.aau1043/suppl_file/aau1043_datas3.gz

GIAB problematic regions, https://hgdownload.soe.ucsc.edu/gbdb/hg38/problematic/GIAB/alldifficultregions.bb

hap-ibd, https://github.com/browning-lab/hap-ibd

ibd-ends, https://github.com/browning-lab/ibd-ends

isweep, https://github.com/sdtemple/isweep

OMIM, https://www.omim.org

tskit, https://tskit.dev/

tskibd, https://github.com/bguo068/tskibd

UCSC Genome Browser, https://genome.ucsc.edu

Supplemental information

Supplemental information can be found online at https://doi.org/10.1016/j.ajhg.2025.09.004.

Received: January 30, 2025
Accepted: September 3, 2025
Published: September 26, 2025

References

1. Vitti, J.J., Grossman, S.R., and Sabeti, P.C. (2013). Detecting natural selection in genomic data. Annu. Rev. Genet. *47*, 97–120. https://doi.org/10.1146/annurev-genet-111212-133526.
2. Pankratov, V., Yunusbaeva, M., Ryakhovsky, S., Zarodniuk, M., Estonian Biobank Research Team, and Yunusbayev, B. (2022). Prioritizing autoimmunity risk variants for functional analyses by fine-mapping mutations under natural selection. Nat. Commun. *13*, 7069. https://doi.org/10.1038/s41467-022-34461-9.
3. Sams, A.J., Dumaine, A., Nédélec, Y., Yotova, V., Alfieri, C., Tanner, J.E., Messer, P.W., and Barreiro, L.B. (2016). Adaptively introgressed Neandertal haplotype at the *OAS* locus functionally impacts innate immune responses in humans. Genome Biol. *17*, 246. https://doi.org/10.1186/s13059-016-1098-6.
4. Anopheles gambiae 1000 Genomes Consortium (2017). Genetic diversity of the African malaria vector Anopheles gambiae. Nature *552*, 96–100. https://doi.org/10.1038/nature24995.
5. Garud, N.R. (2023). Understanding soft sweeps: a signature of rapid adaptation. Nat. Rev. Genet. *24*, 420. https://doi.org/10.1038/s41576-023-00585-x.
6. Guo, B., Borda, V., Laboulaye, R., Spring, M.D., Wojnarski, M., Vesely, B.A., Silva, J.C., Waters, N.C., O'Connor, T.D., and Takala-Harrison, S. (2024). Strong positive selection biases identity-by-descent-based inferences of recent demography and population structure in Plasmodium falciparum. Nat. Commun. *15*, 2499. https://doi.org/10.1038/s41467-024-46659-0.
7. Kimura, M. (1983). The Neutral Theory of Molecular Evolution (Cambridge University Press).
8. Ohta, T. (1973). Slightly deleterious mutant substitutions in evolution. Nature *246*, 96–98. https://doi.org/10.1038/246096a0.
9. Hermisson, J., and Pennings, P.S. (2005). Soft sweeps: molecular population genetics of adaptation from standing genetic variation. Genetics *169*, 2335–2352. https://doi.org/10.1534/genetics.104.036947.
10. Hermisson, J., and Pennings, P.S. (2017). Soft sweeps and beyond: understanding the patterns and probabilities of selection footprints under rapid adaptation. Methods Ecol. Evol. *8*, 700–716. https://doi.org/10.1111/2041-210X.12808.
11. Pennings, P.S., and Hermisson, J. (2006). Soft sweeps III: the signature of positive selection from recurrent mutation. PLoS Genet. *2*, e186. https://doi.org/10.1371/journal.pgen.0020186.
12. Pennings, P.S., and Hermisson, J. (2006). Soft sweeps II—molecular population genetics of adaptation from recurrent mutation or migration. Mol. Biol. Evol. *23*, 1076–1084. https://doi.org/10.1093/molbev/msj117.
13. Crow, J.F., and Kimura, M. (1970). An Introduction to Population Genetics Theory (Harper & Row).
14. Temple, S.D., Waples, R.K., and Browning, S.R. (2024). Modeling recent positive selection using identity-by-descent segments. Am. J. Hum. Genet. *111*, 2510–2529. https://doi.org/10.1016/j.ajhg.2024.08.023.
15. Kreitman, M., and Akashi, H. (1995). Molecular evidence for natural selection. Annu. Rev. Ecol. Syst. *26*, 403–422.
16. McDonald, J.H., and Kreitman, M. (1991). Adaptive protein evolution at the Adh locus in Drosophila. Nature *351*, 652–654. https://doi.org/10.1038/351652a0.
17. Sabeti, P.C., Varilly, P., Fry, B., Lohmueller, J., Hostetter, E., Cotsapas, C., Xie, X., Byrne, E.H., McCarroll, S.A., Gaudet, R., et al. (2007). Genome-wide detection and characterization of positive selection in human populations. Nature *449*, 913–918. https://doi.org/10.1038/nature06250.
18. Salter-Townshend, M., and Myers, S. (2019). Fine-scale inference of ancestry segments without prior knowledge of admixing groups. Genetics *212*, 869–889. https://doi.org/10.1534/genetics.119.302139.
19. Fay, J.C., and Wu, C.I. (2000). Hitchhiking under positive Darwinian selection. Genetics *155*, 1405–1413. https://doi.org/10.1093/genetics/155.3.1405.
20. Field, Y., Boyle, E.A., Telis, N., Gao, Z., Gaulton, K.J., Golan, D., Yengo, L., Rocheleau, G., Froguel, P., McCarthy, M.I., and Pritchard, J.K. (2016). Detection of human adaptation during the past 2000 years. Science *354*, 760–764. https://doi.org/10.1126/science.aag0776.
21. Tajima, F. (1989). Statistical method for testing the neutral mutation hypothesis by DNA polymorphism. Genetics *123*, 585–595. https://doi.org/10.1093/genetics/123.3.585.
22. Akbari, A., Vitti, J.J., Iranmehr, A., Bakhtiari, M., Sabeti, P. C., Mirarab, S., and Bafna, V. (2018). Identifying the favored mutation in a positive selective sweep. Nat. Methods *15*, 279–282. https://doi.org/10.1038/nmeth.4606.
23. Albrechtsen, A., Moltke, I., and Nielsen, R. (2010). Natural selection and the distribution of identity-by-descent in the human genome. Genetics *186*, 295–308. https://doi.org/10.1534/genetics.110.113977.
24. Browning, S.R., and Browning, B.L. (2020). Probabilistic estimation of identity by descent segment endpoints and detection of recent selection. Am. J. Hum. Genet. *107*, 895–910. https://doi.org/10.1016/j.ajhg.2020.09.010.
25. Ferrer-Admetlla, A., Liang, M., Korneliussen, T., and Nielsen, R. (2014). On detecting incomplete soft or hard selective sweeps using haplotype structure. Mol. Biol. Evol. *31*, 1275–1291. https://doi.org/10.1093/molbev/msu077.
26. Garud, N.R., Messer, P.W., Buzbas, E.O., and Petrov, D.A. (2015). Recent selective sweeps in North American Drosophila melanogaster show signatures of soft sweeps. PLoS Genet. *11*, e1005004. https://doi.org/10.1371/journal.pgen.1005004.
27. Harris, A.M., Garud, N.R., and DeGiorgio, M. (2018). Detection and classification of hard and soft sweeps from unphased genotypes by multilocus genotype identity. Genetics *210*, 1429–1452. https://doi.org/10.1534/genetics.118.301502.
28. Nait Saada, J., Kalantzis, G., Shyr, D., Cooper, F., Robinson, M., Gusev, A., and Palamara, P.F. (2020). Identity-by-descent detection across 487,409 British samples reveals fine scale population structure and ultra-rare variant associations. Nat. Commun. *11*, 6130. https://doi.org/10.1038/s41467-020-19588-x.
29. O'Reilly, P.F., Birney, E., and Balding, D.J. (2008). Confounding between recombination and selection, and the Ped/Pop method for detecting selection. Genome Res. *18*, 1304–1313. https://doi.org/10.1101/gr.067181.107.

30. Sabeti, P.C., Reich, D.E., Higgins, J.M., Levine, H.Z.P., Richter, D.J., Schaffner, S.F., Gabriel, S.B., Platko, J.V., Patterson, N.J., McDonald, G.J., et al. (2002). Detecting recent positive selection in the human genome from haplotype structure. Nature *419*, 832–837. https://doi.org/10.1038/nature01140.
31. Voight, B.F., Kudaravalli, S., Wen, X., and Pritchard, J.K. (2006). A map of recent positive selection in the human genome. PLoS Biol. *4*, e72. https://doi.org/10.1371/journal.pbio.0040072.
32. Palamara, P.F., Terhorst, J., Song, Y.S., and Price, A.L. (2018). High-throughput inference of pairwise coalescence times identifies signals of selection and enriched disease heritability. Nat. Genet. *50*, 1311–1317. https://doi.org/10.1038/s41588-018-0177-x.
33. Speidel, L., Forest, M., Shi, S., and Myers, S.R. (2019). A method for genome-wide genealogy estimation for thousands of samples. Nat. Genet. *51*, 1321–1329. https://doi.org/10.1038/s41588-019-0484-x.
34. Stern, A.J., Wilton, P.R., and Nielsen, R. (2019). An approximate full-likelihood method for inferring selection and allele frequency trajectories from DNA sequence data. PLoS Genet. *15*, e1008384. https://doi.org/10.1371/journal.pgen.1008384.
35. Vaughn, A.H., and Nielsen, R. (2024). Fast and accurate estimation of selection coefficients and allele histories from ancient and modern DNA. Mol. Biol. Evol. *41*, msae156. https://doi.org/10.1093/molbev/msae156.
36. Peter, B.M., Huerta-Sanchez, E., and Nielsen, R. (2012). Distinguishing between selective sweeps from standing variation and from a de novo mutation. PLoS Genet. *8*, e1003011. https://doi.org/10.1371/journal.pgen.1003011.
37. Mathieson, I., and Terhorst, J. (2022). Direct detection of natural selection in Bronze Age Britain. Genome Res. *32*, 2057–2067. https://doi.org/10.1101/gr.276862.122.
38. Mathieson, I., Lazaridis, I., Rohland, N., Mallick, S., Patterson, N., Roodenberg, S.A., Harney, E., Stewardson, K., Fernandes, D., Novak, M., et al. (2015). Genome-wide patterns of selection in 230 ancient Eurasians. Nature *528*, 499–503. https://doi.org/10.1038/nature16152.
39. Hejase, H.A., Mo, Z., Campagna, L., and Siepel, A. (2022). A deep-learning approach for inference of selective sweeps from the ancestral recombination graph. Mol. Biol. Evol. *39*, msab332. https://doi.org/10.1093/molbev/msab332.
40. Kern, A.D., and Schrider, D.R. (2018). diploS/HIC: an updated approach to classifying selective sweeps. G3: Genes, Genomes, Genetics. *8*, 1959–1970. https://doi.org/10.1534/g3.118.200262.
41. Mo, Z., and Siepel, A. (2023). Domain-adaptive neural networks improve supervised machine learning based on simulated population genetic data. PLoS Genet. *19*, e1011032. https://doi.org/10.1371/journal.pgen.1011032.
42. Riley, R., Mathieson, I., and Mathieson, S. (2024). Interpreting generative adversarial networks to infer natural selection from genetic data. Genetics *226*, iyae024. https://doi.org/10.1093/genetics/iyae024.
43. Schrider, D.R., and Kern, A.D. (2016). S/HIC: Robust identification of soft and hard sweeps using machine learning. PLoS Genet. *12*, e1005928. https://doi.org/10.1371/journal.pgen.1005928.
44. Whitehouse, L.S., and Schrider, D.R. (2023). Timesweeper: accurately identifying selective sweeps using population genomic time series. Genetics *224*, iyad084. https://doi.org/10.1093/genetics/iyad084.
45. Stern, A.J., and Nielsen, R. (2019). Detecting natural selection. In Handbook of Statistical Genomics: Two Volume Set (Wiley), p. 397. https://doi.org/10.1002/9781119487845.ch14.
46. Sidak, Z. (1967). Rectangular confidence regions for the means of multivariate normal distributions. J. Am. Stat. Assoc. *62*, 626–633. https://doi.org/10.1080/01621459.1967.10482935.
47. Benjamini, Y., and Hochberg, Y. (1995). Controlling the false discovery rate: a practical and powerful approach to multiple testing. J. R. Stat. Soc. Series B Stat. Methodol. *57*, 289–300. https://doi.org/10.1111/j.2517-6161.1995.tb02031.x.
48. Hernandez, M., and Perry, G.H. (2021). Scanning the human genome for "signatures" of positive selection: transformative opportunities and ethical obligations. Evol. Anthropol. *30*, 113–121. https://doi.org/10.1002/evan.21893.
49. Chen, Z., Boehnke, M., Wen, X., and Mukherjee, B. (2021). Revisiting the genome-wide significance threshold for common variant GWAS. G3: Genes, Genomes, Genetics *11*, jkaa056. https://doi.org/10.1093/g3journal/jkaa056.
50. Fadista, J., Manning, A.K., Florez, J.C., and Groop, L. (2016). The (in) famous GWAS P-value threshold revisited and updated for low-frequency variants. Eur. J. Hum. Genet. *24*, 1202–1205. https://doi.org/10.1038/ejhg.2015.269.
51. Browning, S.R., and Thompson, E.A. (2012). Detecting rare variant associations by identity-by-descent mapping in case-control studies. Genetics *190*, 1521–1531. https://doi.org/10.1534/genetics.111.136937.
52. Conneely, K.N., and Boehnke, M. (2007). So many correlated tests, so little time! Rapid adjustment of P values for multiple correlated tests. Am. J. Hum. Genet. *81*, 1158–1168. https://doi.org/10.1086/522036.
53. Grinde, K.E. (2019). Statistical Inference in Admixed Populations. Ph.D. thesis (University of Washington).
54. Grinde, K.E., Brown, L.A., Reiner, A.P., Thornton, T.A., and Browning, S.R. (2019). Genome-wide significance thresholds for admixture mapping studies. Am. J. Hum. Genet. *104*, 454–465. https://doi.org/10.1016/j.ajhg.2019.01.008.
55. Seaman, S.R., and Müller-Myhsok, B. (2005). Rapid simulation of P values for product methods and multiple-testing adjustment in association studies. Am. J. Hum. Genet. *76*, 399–408. https://doi.org/10.1086/428140.
56. Siegmund, D., and Yakir, B. (2007). The Statistics of Gene Mapping (Springer). https://doi.org/10.1007/978-0-387-49686-3.
57. Feingold, E., Brown, P.O., and Siegmund, D. (1993). Gaussian models for genetic linkage analysis using complete high-resolution maps of identity by descent. Am. J. Hum. Genet. 53, 234–251.
58. Ségurel, L., and Bon, C. (2017). On the evolution of lactase persistence in humans. Annu. Rev. Genomics Hum. Genet. *18*, 297–319. https://doi.org/10.1146/annurev-genom-091416-035340.
59. Taliun, D., Harris, D.N., Kessler, M.D., Carlson, J., Szpiech, Z.A., Torres, R., Taliun, S.A.G., Corvelo, A., Gogarten, S. M., Kang, H.M., et al. (2021). Sequencing of 53,831 diverse genomes from the NHLBI TOPMed program. Nature *590*, 290–299. https://doi.org/10.1038/s41586-021-03205-y.
60. Granka, J.M., Henn, B.M., Gignoux, C.R., Kidd, J.M., Bustamante, C.D., and Feldman, M.W. (2012). Limited evidence

for classic selective sweeps in African populations. Genetics *192*, 1049–1064. https://doi.org/10.1534/genetics.112.144071.

61. Temple, S.D., and Thompson, E.A. (2025). Identity-by-descent segments in large samples. Theor. Popul. Biol. *165*, 10–21. https://doi.org/10.1016/j.tpb.2025.06.003.
62. Temple, S.D. (2024). Statistical Inference Using Identity-By-Descent Segments: Perspectives on Recent Positive Selection. Ph.D. thesis (University of Washington).
63. Murphy, D.A., Elyashiv, E., Amster, G., and Sella, G. (2023). Broad-scale variation in human genetic diversity levels is predicted by purifying selection on coding and non-coding elements. eLife *12*, e76065. https://doi.org/10.7554/eLife.76065.
64. Grinde, K.E., Browning, B.L., Reiner, A.P., Thornton, T.A., and Browning, S.R. (2024). Adjusting for principal components can induce collider bias in genome-wide association studies. PLoS Genet. *20*, e1011242. https://doi.org/10.1371/journal.pgen.1011242.
65. Baumdicker, F., Bisschop, G., Goldstein, D., Gower, G., Ragsdale, A.P., Tsambos, G., Zhu, S., Eldon, B., Ellerman, E.C., Galloway, J.G., et al. (2022). Efficient ancestry and mutation simulation with msprime 1.0. Genetics *220*, iyab229. https://doi.org/10.1093/genetics/iyab229.
66. Temple, S.D., Browning, S.R., and Thompson, E.A. (2025). Fast simulation of identity-by-descent segments. Bull. Math. Biol. *87*, 84. https://doi.org/10.1007/s11538-025-01464-8.
67. Fisher, R.A. (1923). XXI.—on the dominance ratio. Proc. R. Soc. Edinb. *42*, 321–341. https://doi.org/10.1017/S0370164600023993.
68. Haldane, J.B.S. (1924). A mathematical theory of natural and artificial selection. Part I. Math. Proc. Camb. Philos. Soc. *23*, 19–41. https://doi.org/10.1017/S0305004100011750.
69. Wright, S. (1931). Evolution in Mendelian populations. Genetics *16*, 97–159. https://doi.org/10.1093/genetics/16.2.97.
70. Kern, A.D., and Schrider, D.R. (2016). Discoal: flexible coalescent simulations with selection. Bioinformatics *32*, 3839–3841. https://doi.org/10.1093/bioinformatics/btw556.
71. Bycroft, C., Freeman, C., Petkova, D., Band, G., Elliott, L.T., Sharp, K., Motyer, A., Vukcevic, D., Delaneau, O., O'Connell, J., et al. (2018). The UK Biobank resource with deep phenotyping and genomic data. Nature *562*, 203–209. https://doi.org/10.1038/s41586-018-0579-z.
72. Browning, B.L., Tian, X., Zhou, Y., and Browning, S.R. (2021). Fast two-stage phasing of large-scale sequence data. Am. J. Hum. Genet. *108*, 1880–1890. https://doi.org/10.1016/j.ajhg.2021.08.005.
73. Gogarten, S.M., Sofer, T., Chen, H., Yu, C., Brody, J.A., Thornton, T.A., Rice, K.M., and Conomos, M.P. (2019). Genetic association testing using the GENESIS R/Bioconductor package. Bioinformatics *35*, 5346–5348. https://doi.org/10.1093/bioinformatics/btz567.
74. Zheng, X., Levine, D., Shen, J., Gogarten, S.M., Laurie, C., and Weir, B.S. (2012). A high-performance computing toolset for relatedness and principal component analysis of SNP data. Bioinformatics *28*, 3326–3328. https://doi.org/10.1093/bioinformatics/bts606.
75. Alexander, D.H., Novembre, J., and Lange, K. (2009). Fast model-based estimation of ancestry in unrelated individuals. Genome Res. *19*, 1655–1664. https://doi.org/10.1101/gr.094052.109.
76. Wu, Y., Gettler, K., Kars, M.E., Giri, M., Li, D., Bayrak, C.S., Zhang, P., Jain, A., Maffucci, P., Sabic, K., et al. (2023). Identifying high-impact variants and genes in exomes of Ashkenazi Jewish inflammatory bowel disease patients. Nat. Commun. *14*, 2256. https://doi.org/10.1038/s41467-023-37849-3.
77. Browning, S.R., and Browning, B.L. (2015). Accurate non-parametric estimation of recent effective population size from segments of identity by descent. Am. J. Hum. Genet. *97*, 404–418. https://doi.org/10.1016/j.ajhg.2015.07.012.
78. Carmi, S., Hui, K.Y., Kochav, E., Liu, X., Xue, J., Grady, F., Guha, S., Upadhyay, K., Ben-Avraham, D., Mukherjee, S., et al. (2014). Sequencing an Ashkenazi reference panel supports population-targeted personal genomics and illuminates Jewish and European origins. Nat. Commun. *5*, 4835. https://doi.org/10.1038/ncomms5835.
79. Tian, X., Browning, B.L., and Browning, S.R. (2019). Estimating the genome-wide mutation rate with three-way identity by descent. Am. J. Hum. Genet. *105*, 883–893. https://doi.org/10.1016/j.ajhg.2019.09.012.
80. Raj, A., Stephens, M., and Pritchard, J.K. (2014). fastSTRUCTURE: variational inference of population structure in large SNP data sets. Genetics *197*, 573–589. https://doi.org/10.1534/genetics.114.164350.
81. International HapMap Consortium (2003). The international HapMap project. Nature *426*, 789–796. https://doi.org/10.1038/nature02168.
82. Byrska-Bishop, M., Evani, U.S., Zhao, X., Basile, A.O., Abel, H.J., Regier, A.A., Corvelo, A., Clarke, W.E., Musunuri, R., Nagulapalli, K., et al. (2022). High-coverage whole-genome sequencing of the expanded 1000 Genomes Project cohort including 602 trios. Cell *185*, 3426–3440.e19. https://doi.org/10.1016/j.cell.2022.08.004.
83. Halldorsson, B.V., Palsson, G., Stefansson, O.A., Jonsson, H., Hardarson, M.T., Eggertsson, H.P., Gunnarsson, B., Oddsson, A., Halldorsson, G.H., Zink, F., et al. (2019). Characterizing mutagenic effects of recombination through a sequence-level genetic map. Science *363*, eaau1043. https://doi.org/10.1126/science.aau1043.
84. Cai, R., Browning, B.L., and Browning, S.R. (2023). Identity-by-descent-based estimation of the X chromosome effective population size with application to sex-specific demographic history. G3: Genes, Genomes, Genetics *13*, jkad165. https://doi.org/10.1093/g3journal/jkad165.
85. Bhérer, C., Campbell, C.L., and Auton, A. (2017). Refined genetic maps reveal sexual dimorphism in human meiotic recombination at multiple scales. Nat. Commun. *8*, 14994. https://doi.org/10.1038/ncomms14994.
86. Temple, S.D., Chapman, N.H., Choi, S.H., DeStefano, A.L., Thornton, T.A., Wijsman, E.M., and Blue, E.E. (2025). Multiple-testing corrections in case-control studies using identity-by-descent segments. Preprint at bioRxiv. https://doi.org/10.1101/2025.07.03.663057.
87. Amemiya, H.M., Kundaje, A., and Boyle, A.P. (2019). The ENCODE blacklist: Identification of problematic regions of the genome. Sci. Rep. *9*, 9354. https://doi.org/10.1038/s41598-019-45839-z.
88. Zook, J.M., Chapman, B., Wang, J., Mittelman, D., Hofmann, O., Hide, W., and Salit, M. (2014). Integrating human sequence data sets provides a resource of benchmark

SNP and indel genotype calls. Nat. Biotechnol. *32*, 246–251. https://doi.org/10.1038/nbt.2835.

89. Karczewski, K.J., Gupta, R., Kanai, M., Lu, W., Tsuo, K., Wang, Y., Walters, R.K., Turley, P., Callier, S., Shah, N.N., et al. (2024). Pan-UK Biobank GWAS improves discovery, analysis of genetic architecture, and resolution into ancestry-enriched effects. Preprint at bioRxiv. https://doi.org/10.1101/2024.03.13.24303864.
90. Wetzel, A.S., and Darbro, B.W. (2022). A comprehensive list of human microdeletion and microduplication syndromes. BMC Genom. Data *23*, 82. https://doi.org/10.1186/s12863-022-01093-3.
91. Gittelman, R.M., Schraiber, J.G., Vernot, B., Mikacenic, C., Wurfel, M.M., and Akey, J.M. (2016). Archaic hominin admixture facilitated adaptation to Out-of-Africa environments. Curr. Biol. *26*, 3375–3382. https://doi.org/10.1016/j.cub.2016.10.041.
92. Horikawa, Y., Iwasaki, N., Hara, M., Furuta, H., Hinokio, Y., Cockburn, B.N., Lindner, T., Yamagata, K., Ogata, M., Tomonaga, O., et al. (1997). Mutation in hepatocyte nuclear factor-1ß gene (*TCF2*) associated with MODY. Nat. Genet. *17*, 384–385. https://doi.org/10.1038/ng1297-384.
93. Gudmundsson, J., Sulem, P., Steinthorsdottir, V., Bergthorsson, J.T., Thorleifsson, G., Manolescu, A., Rafnar, T., Gudbjartsson, D., Agnarsson, B.A., Baker, A., et al. (2007). Two variants on chromosome 17 confer prostate cancer risk, and the one in *TCF2* protects against type 2 diabetes. Nat. Genet. *39*, 977–983. https://doi.org/10.1038/ng2062.
94. Papadakis, K.A., Prehn, J., Nelson, V., Cheng, L., Binder, S. W., Ponath, P.D., Andrew, D.P., and Targan, S.R. (2000). The role of thymus-expressed chemokine and its receptor *CCR9* on lymphocytes in the regional specialization of the mucosal immune system. J. Immunol. *165*, 5069–5076. https://doi.org/10.4049/jimmunol.165.9.5069.
95. Shelton, J.F., Shastri, A.J., Ye, C., Weldon, C.H., Filshtein-Sonmez, T., Coker, D., Symons, A., Esparza-Gordillo, J., 23andMe COVID-19 Team, Aslibekyan, S., and Auton, A. (2021). Trans-ancestry analysis reveals genetic and nongenetic associations with COVID-19 susceptibility and severity. Nat. Genet. *53*, 801–808. https://doi.org/10.1038/s41588-021-00854-7.
96. Browning, S.R., Browning, B.L., Zhou, Y., Tucci, S., and Akey, J.M. (2018). Analysis of human sequence data reveals two pulses of archaic Denisovan admixture. Cell *173*, 53–61.e9. https://doi.org/10.1016/j.cell.2018.02.031.
97. Ding, Q., Hu, Y., Xu, S., Wang, J., and Jin, L. (2014). Neanderthal introgression at chromosome 3p21.31 was under positive natural selection in East Asians. Mol. Biol. Evol. *31*, 683–695. https://doi.org/10.1093/molbev/mst260.
98. Stefansson, H., Helgason, A., Thorleifsson, G., Steinthorsdottir, V., Masson, G., Barnard, J., Baker, A., Jonasdottir, A., Ingason, A., Gudnadottir, V.G., et al. (2005). A common inversion under selection in Europeans. Nat. Genet. *37*, 129–137. https://doi.org/10.1038/ng1508.
99. Gallego Romero, I., Basu Mallick, C., Liebert, A., Crivellaro, F., Chaubey, G., Itan, Y., Metspalu, M., Eaaswarkhanth, M., Pitchappan, R., Villems, R., et al. (2012). Herders of Indian and European cattle share their predominant allele for lactase persistence. Mol. Biol. Evol. *29*, 249–260. https://doi.org/10.1093/molbev/msr190.
100. Kidd, K.K., Pakstis, A.J., Donnelly, M.P., Bulbul, O., Cherni, L., Gurkan, C., Kang, L., Li, H., Yun, L., Paschou, P., et al. (2020). The distinctive geographic patterns of common pigmentation variants at the *OCA2* gene. Sci. Rep. *10*, 15433. https://doi.org/10.1038/s41598-020-72262-6.
101. Lewis, M.J., Vyse, S., Shields, A.M., Boeltz, S., Gordon, P.A., Spector, T.D., Lehner, P.J., Walczak, H., and Vyse, T.J. (2015). *UBE2L3* polymorphism amplifies NF-κB activation and promotes plasma cell development, linking linear ubiquitination to multiple autoimmune diseases. Am. J. Hum. Genet. *96*, 221–234. https://doi.org/10.1016/j.ajhg.2014.12.024.
102. Burnside, R.D. (2015). 22q11.21 deletion syndromes: A review of proximal, central, and distal deletions and their associated features. Cytogenet. Genome Res. *146*, 89–99. https://doi.org/10.1159/000438708.
103. Schreml, J., Durmaz, B., Cogulu, O., Keupp, K., Beleggia, F., Pohl, E., Milz, E., Coker, M., Ucar, S.K., Nürnberg, G., et al. (2014). The missing "link": an autosomal recessive short stature syndrome caused by a hypofunctional *XYLT1* mutation. Hum. Genet. *133*, 29–39. https://doi.org/10.1007/s00439-013-1351-y.
104. Mis, E.K., Liem, K.F., Jr., Kong, Y., Schwartz, N.B., Domowicz, M., and Weatherbee, S.D. (2014). Forward genetics defines *XYLT1* as a key, conserved regulator of early chondrocyte maturation and skeletal length. Dev. Biol. *385*, 67–82. https://doi.org/10.1016/j.ydbio.2013.10.014.
105. Tropeano, M., Andrieux, J., and Collier, D.A. (2014). Clinical utility gene card for: 16p13.11 microdeletion syndrome. Eur. J. Hum. Genet. *22*, 713. https://doi.org/10.1038/ejhg.2013.230.
106. Oster, S.F., Bodeker, M.O., He, F., and Sretavan, D.W. (2003). Invariant *SEMA5A* inhibition serves an ensheathing function during optic nerve development. Development *130*, 775–784. https://doi.org/10.1242/dev.00299.
107. Kim, U., Wooding, S., Ricci, D., Jorde, L.B., and Drayna, D. (2005). Worldwide haplotype diversity and coding sequence variation at human bitter taste receptor loci. Hum. Mutat. *26*, 199–204. https://doi.org/10.1002/humu.20203.
108. Hao, J., Han, X., Huang, H., Yu, X., Fang, J., Zhao, J., Prayson, R.A., Bao, S., and Yu, J.S. (2023). *SEMA3C* signaling is an alternative activator of the canonical WNT pathway in glioblastoma. Nat. Commun. *14*, 2262. https://doi.org/10.1038/s41467-023-37397-w.
109. Hardison, R.C. (2012). Evolution of hemoglobin and its genes. Cold Spring Harb. Perspect. Med. *2*, a011627. https://doi.org/10.1101/cshperspect.a011627.
110. Tishkoff, S.A., and Williams, S.M. (2002). Genetic analysis of African populations: human evolution and complex disease. Nat. Rev. Genet. *3*, 611–621. https://doi.org/10.1038/nrg865.
111. Freudiger, A., Jovanovic, V.M., Huang, Y., Snyder-Mackler, N., Conrad, D.F., Miller, B., Montague, M.J., Westphal, H., Stadler, P.F., Bley, S., et al. (2025). Estimating realized relatedness in free-ranging macaques by inferring identity-by-descent segments. Proc. Natl. Acad. Sci. USA *122*, e2401106122. https://doi.org/10.1073/pnas.240110612.
112. Ringbauer, H., Huang, Y., Akbari, A., Mallick, S., Olalde, I., Patterson, N., and Reich, D. (2024). Accurate detection of identity-by-descent segments in human ancient DNA. Nat.

Genet. *56*, 143–151. https://doi.org/10.1038/s41588-023-01582-w.

113. Whitehouse, L.S., Ray, D.D., and Schrider, D.R. (2024). Tree sequences as a general-purpose tool for population genetic inference. Mol. Biol. Evol. *41*, msae223. https://doi.org/10.1093/molbev/msae223.
114. Akbari, A., Barton, A.R., Gazal, S., Li, Z., Kariminejad, M., Perry, A., Zeng, Y., Mittnik, A., Patterson, N., Mah, M., et al. (2024). Pervasive findings of directional selection realize the promise of ancient DNA to elucidate human adaptation. Preprint at bioRxiv. https://doi.org/10.1101/2024.09.14.613021.
115. Gusev, A., Palamara, P.F., Aponte, G., Zhuang, Z., Darvasi, A., Gregersen, P., and Pe'er, I. (2012). The architecture of long-range haplotypes shared within and across populations. Mol. Biol. Evol. *29*, 473–486. https://doi.org/10.1093/molbev/msr133.
116. Shemirani, R., Belbin, G.M., Cullina, S., Caggiano, C., Gignoux, C., Zaitlen, N., and Kenny, E.E. (2025). SPC: a SPectral component approach to address recent population structure in genomic analysis. Preprint at medRxiv. https://doi.org/10.1101/2025.06.04.25328990.
117. Maller, R.A., Müller, G., and Szimayer, A. (2009). Ornstein–Uhlenbeck Processes and Extensions (Springer), pp. 421–437.
118. Amorim, C.E., Nunes, K., Meyer, D., Comas, D., Bortolini, M.C., Salzano, F.M., and Hünemeier, T. (2017). Genetic signature of natural selection in first Americans. Proc. Natl. Acad. Sci. USA *114*, 2195–2199. https://doi.org/10.1073/pnas.1620541114.
119. Bryk, J., Hardouin, E., Pugach, I., Hughes, D., Strotmann, R., Stoneking, M., and Myles, S. (2008). Positive selection in East Asians for an *EDAR* allele that enhances NF-κb activation. PLoS One *3*, e2209. https://doi.org/10.1371/journal.pone.0002209.

The American Journal of Human Genetics, Volume *112*

Supplemental information

Multiple-testing corrections in selection scans using identity-by-descent segments

Seth D. Temple and Sharon R. Browning

Supplemental figures

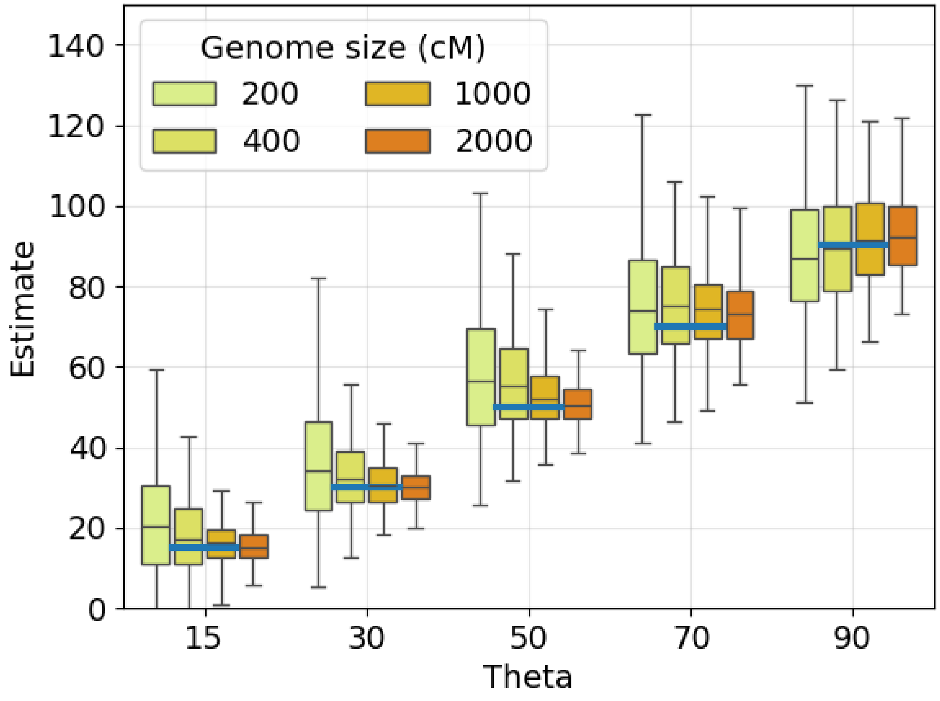

Figure S1: Estimating the exponential decay parameter θ from simulated Ornstein-Uhlenbeck processes. The 1st, 25th, 50th, 75th, and 99th percentiles of estimates $\hat{\theta}$ (y-axis) are shown for true θ (x-axis and horizontal blue lines). We estimate θ with different genome lengths (colors in legend) and step size 0.02 cM. Percentiles are taken over five hundred simulations for each θ.

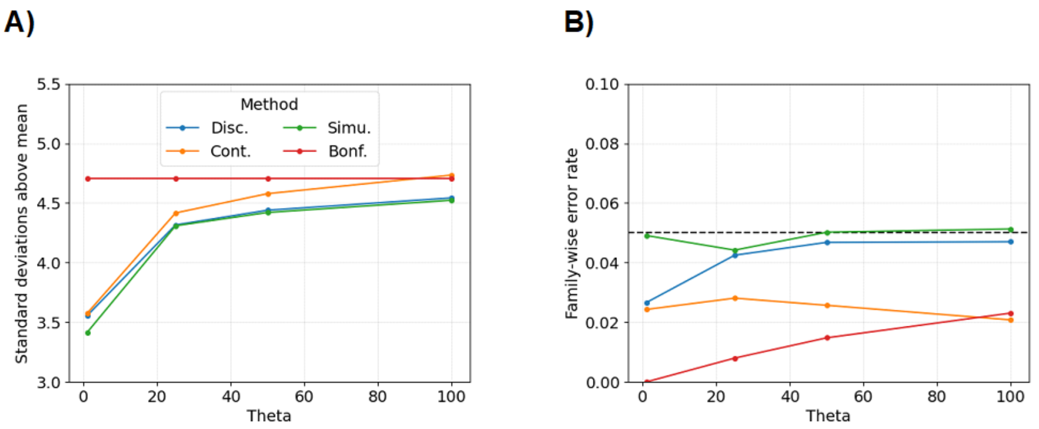

Figure S2: Multiple-testing approaches in simulations of Ornstein-Uhlenbeck processes. Line plots show A) standard deviations above the mean thresholds or B) family-wise error rates (y-axis) with different θ (x-axis). The multiple-testing approaches are (blue) the discrete-spacing analytical approximation, (orange) the continuous-spacing analytical approximation, (green) the simulation-based approach, and (red) the Bonferroni correction. The simulation-based approach is based on ten thousand simulations. The step size is hypothesis testing every 0.05 cM (50 kb). The data for each simulation is equivalent to twenty chromosomes, each of length 100 cM.

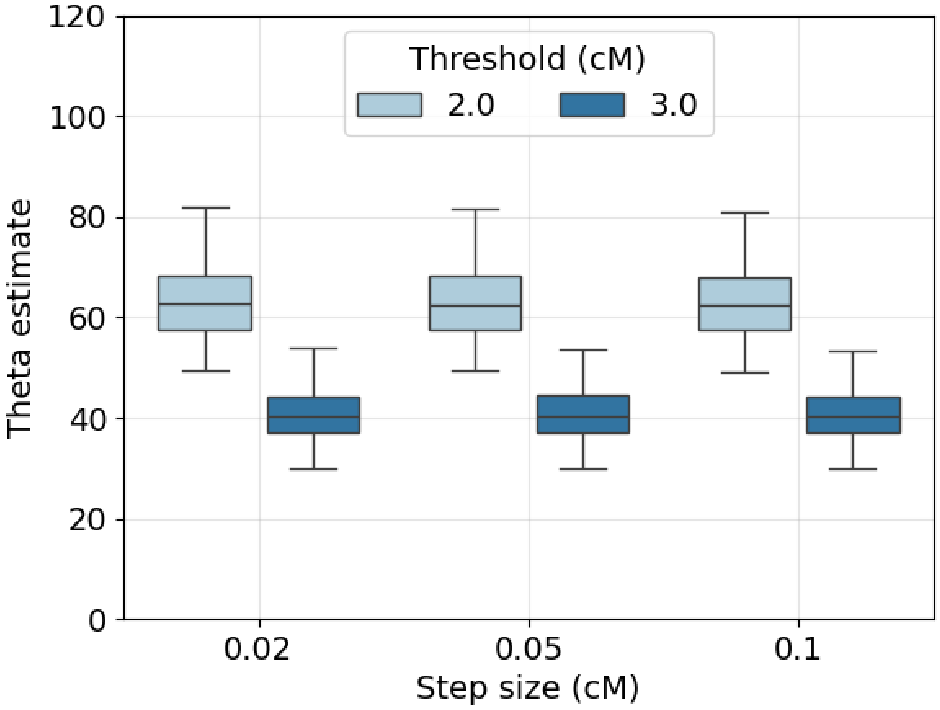

Figure S3: Estimating the exponential decay parameter θ from simulated IBD rate processes with different cM length thresholds. Box plots show the 1st, 25th, 50th, 75th, and 99th percentiles of estimates $\hat{\theta}$ using the IBD rate processes with simulated true IBD segments (dark blue) ≥ 2.0 cM and (light blue) ≥ 3.0 cM from `tskibd`. Estimates $\hat{\theta}$ are based on autocovariances calculated at different step sizes (x-axis). There are fifteen hundred simulations for each step size. The demographic model is the population bottleneck. The data for each simulation is equivalent to ten chromosomes of uniform length 100 cM.

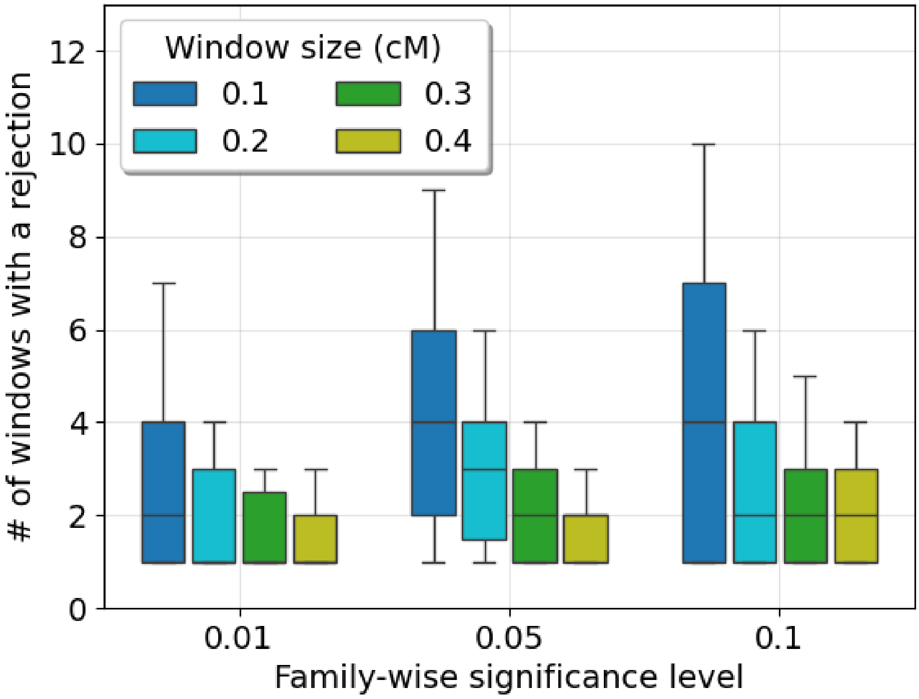

Figure S4: The number of windows with a rejected hypothesis test. Box plots show the 10th, 25th, 50th, 75th, and 90th percentiles of the number of non-overlapping windows with at least one rejection of the null hypothesis (y-axis). Windows sizes are 0.1, 0.2, 0.3, and 0.4 cM (colors in legend) with IBD rates calculated every 0.02 cM. Simulations in which there are no genome-wide significant tests are not included in the box plots. The multiple-testing method is the discrete-spacing analytical approximation using true IBD segments $\geq$ 2.0 cM. There are five hundred simulations for each family-wise significance level (x-axis). The demographic model is the population bottleneck. The data for each simulation is equivalent to ten chromosomes of uniform length 100 cM.

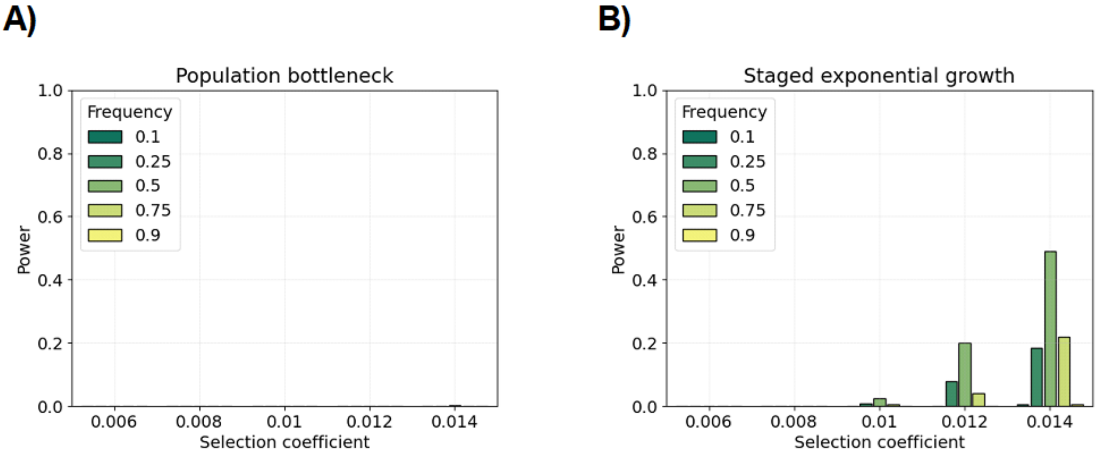

Figure S5: Power simulations for the ≥ 3.0 cM scan in different demographic models. Bar plots show the statistical power (y-axis) in the A) population bottleneck and B) staged exponential growth models using true IBD segments ≥ 3.0 cM overlapping the selected allele. Power is the proportion of tests where the null model is rejected at the p value threshold corresponding to the 0.05 family-wise significance level. Hypothesis testing is based on the discrete-spacing analytical threshold using the step size 0.02 cM. There are two hundred simulations for each pair of selection coefficient (x-axis) and current-day allele frequency (colors in legend).

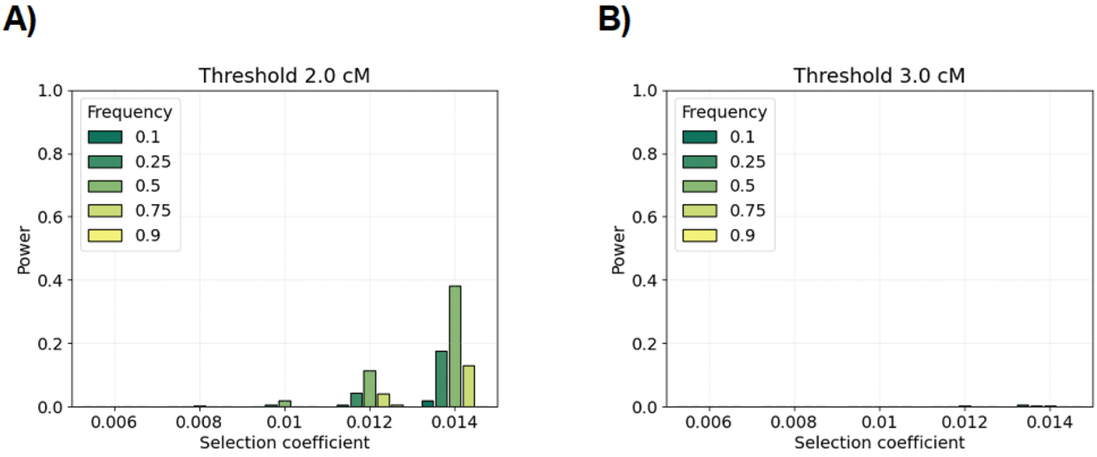

Figure S6: Power simulations in a constant size population. Bar plots show the statistical power (y-axis) using true IBD segments A) ≥ 2.0 cM or B) ≥ 3.0 cM overlapping the selected allele. Power is the proportion of tests where the null model is rejected at the p value threshold corresponding to the 0.05 family-wise significance level. Hypothesis testing is based on the discrete-spacing analytical threshold using the step size 0.02 cM. There are two hundred simulations for each pair of selection coefficient (x-axis) and current-day allele frequency (colors in legend).

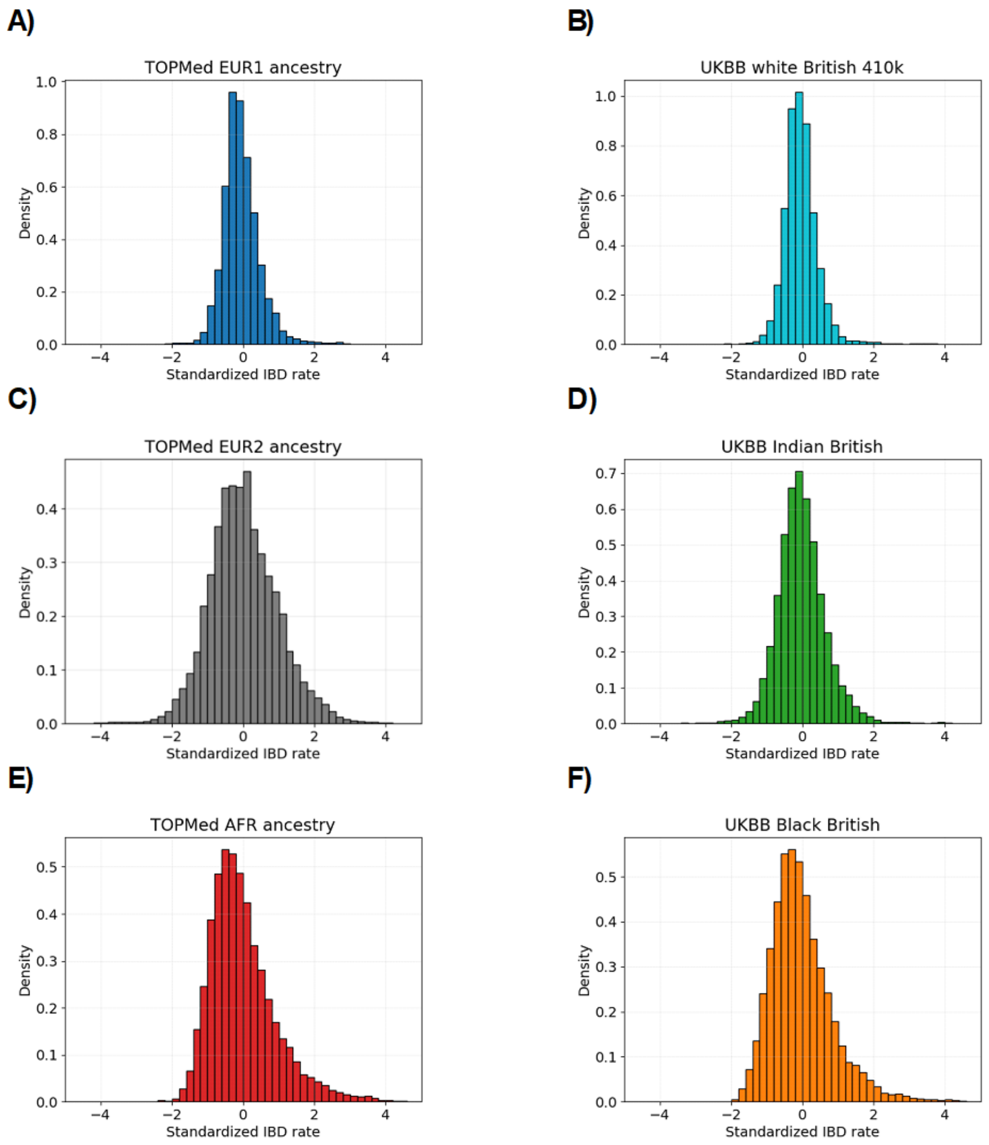

Figure S7: Histograms of IBD rates in human populations. The standardized IBD rates ≥ 2.0 cM (x-axis) are shown for A) TOPMed EUR1, B) UKBB white British, C) TOPMed EUR2, D) UKBB Indian British, E) TOPMed AFR ancestry, and F) UKBB Black British sample sets. Each histogram has fifty bins, and the x-axes range from -5 to 5.

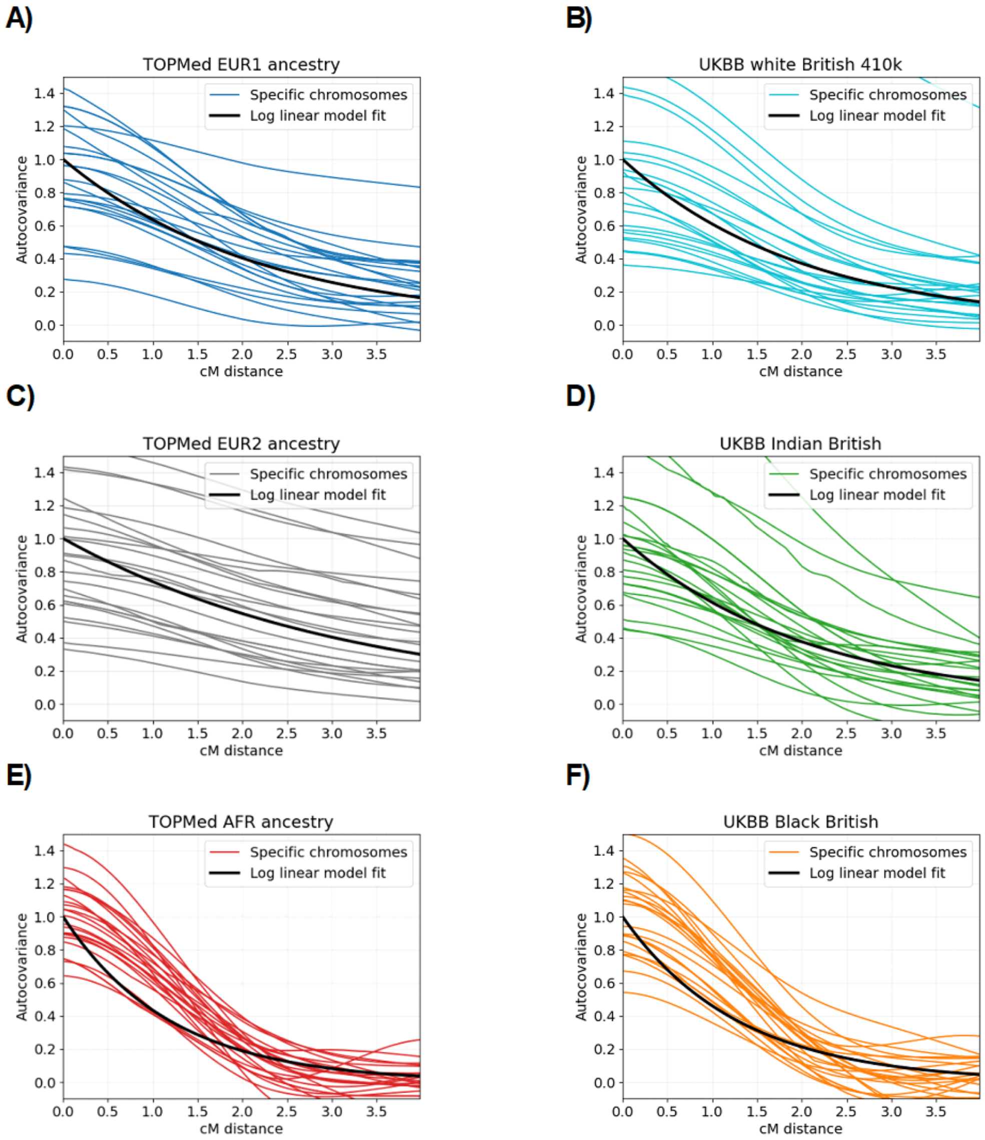

Figure S8: Estimating exponential decay parameter θ in real data. Each faint colored line shows estimated autocovariances (y-axis) for different cM distances (x-axis) and a specific chromosome. The black lines show the predicted autocovariances from the fitted Ornstein-Uhlenbeck processes using estimates $\hat{\theta}$. The data for each subplot is based on A) TOPMed EUR1 ancestry, B) UKBB white British, C) TOPMed EUR2 ancestry, D) UKBB Indian British, E) TOPMed AFR ancestry, and F) UKBB Black British sample sets.

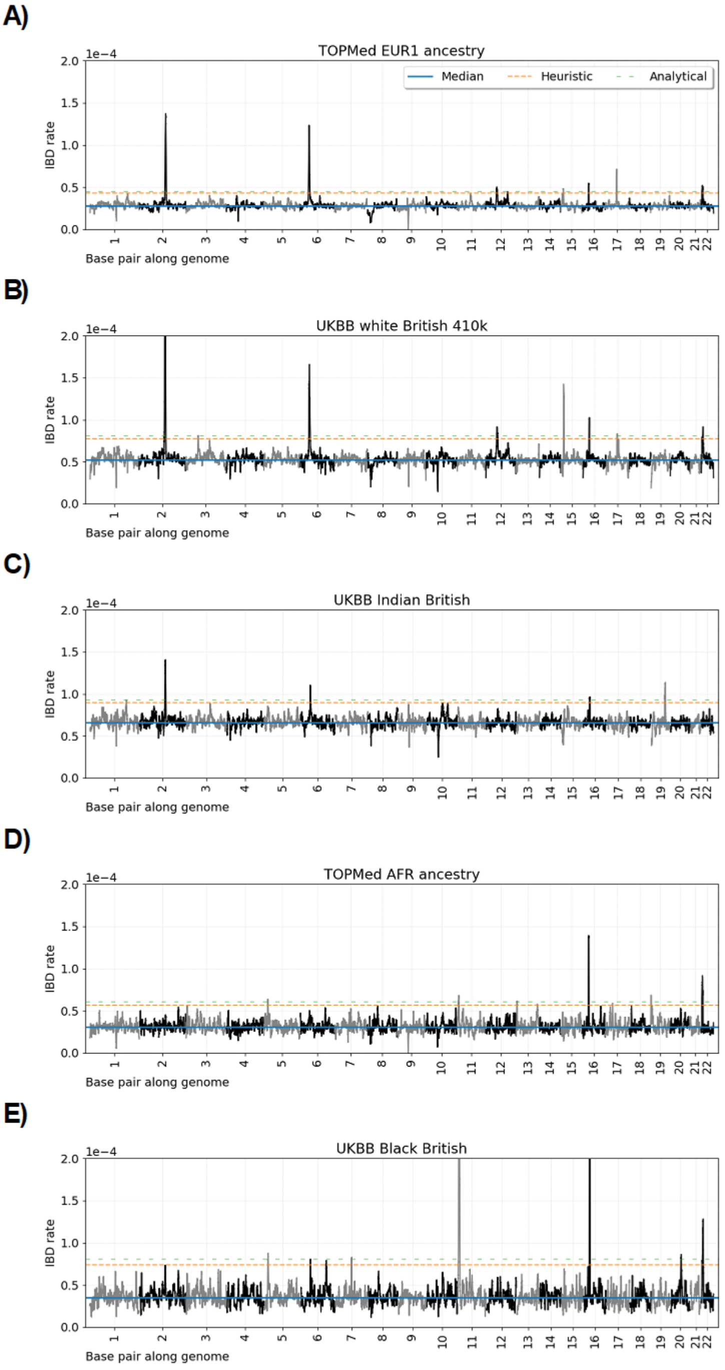

Figure S9: Genome-wide IBD rate scans using the ≥ 3.0 cM threshold. Line plots show IBD rates every 0.02 cM (y-axis) for base pair positions along twenty-two human autosomes. The data for each subplot is based on A) TOPMed EUR1 ancestry, B) UKBB white British, C) UKBB Indian British, D) TOPMed AFR ancestry, and E) UKBB Black British sample sets. Horizontal dashed lines show (blue) the genome-wide median IBD rate, (orange) the heuristic threshold of four standard deviations above the median, and (green) the analytical multiple-testing threshold.

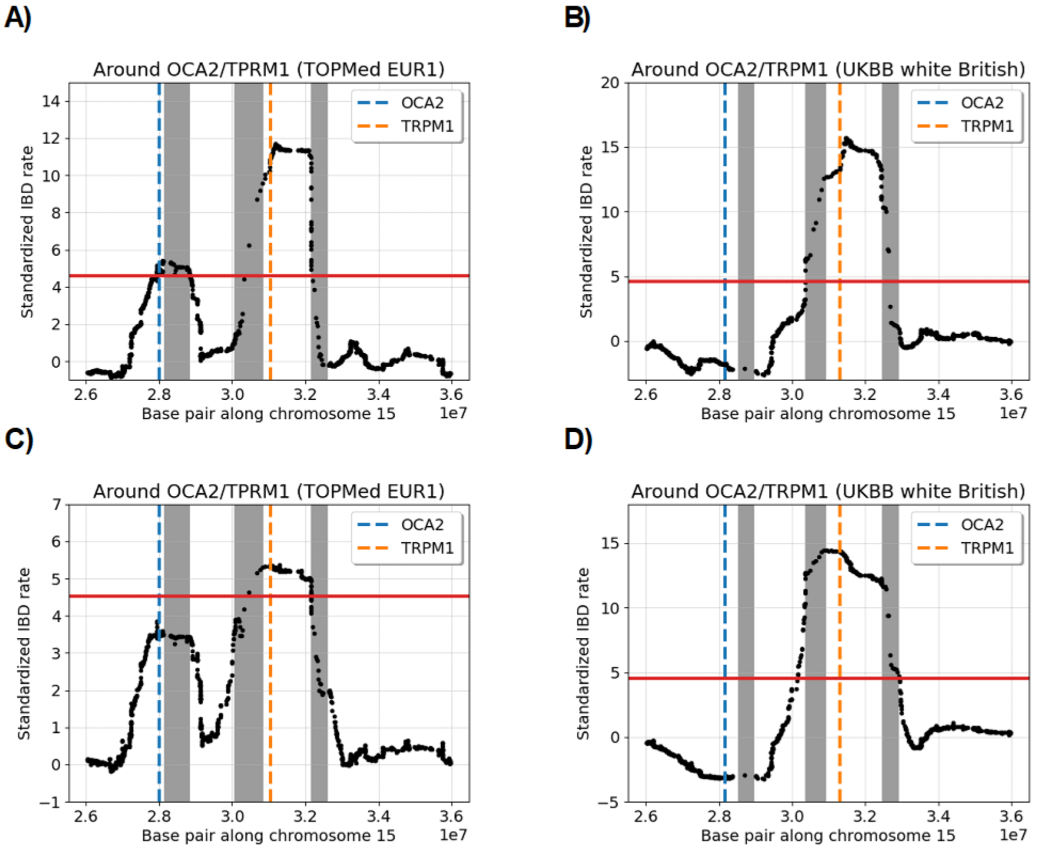

Figure S10: IBD rates around pigmentation genes in European ancestry samples. Scatter plots show the standardized IBD rates by physical base pair (chromosome 15) for A,C) TOPMed EUR1 and B,D) UKBB white British samples. The IBD segment detection thresholds are A-B) 2.0 cM and C-D) 3.0 cM. The horizontal red lines mark the genome-wide significance thresholds. The vertical blue and orange dashed lines mark the approximate midpoints of *OCA2* and *TRPM1*. The y-axis scales may differ to accentuate the dataset-specific signal. The physical base pairs are with respect to different assemblies. Problematic regions are shaded in gray.

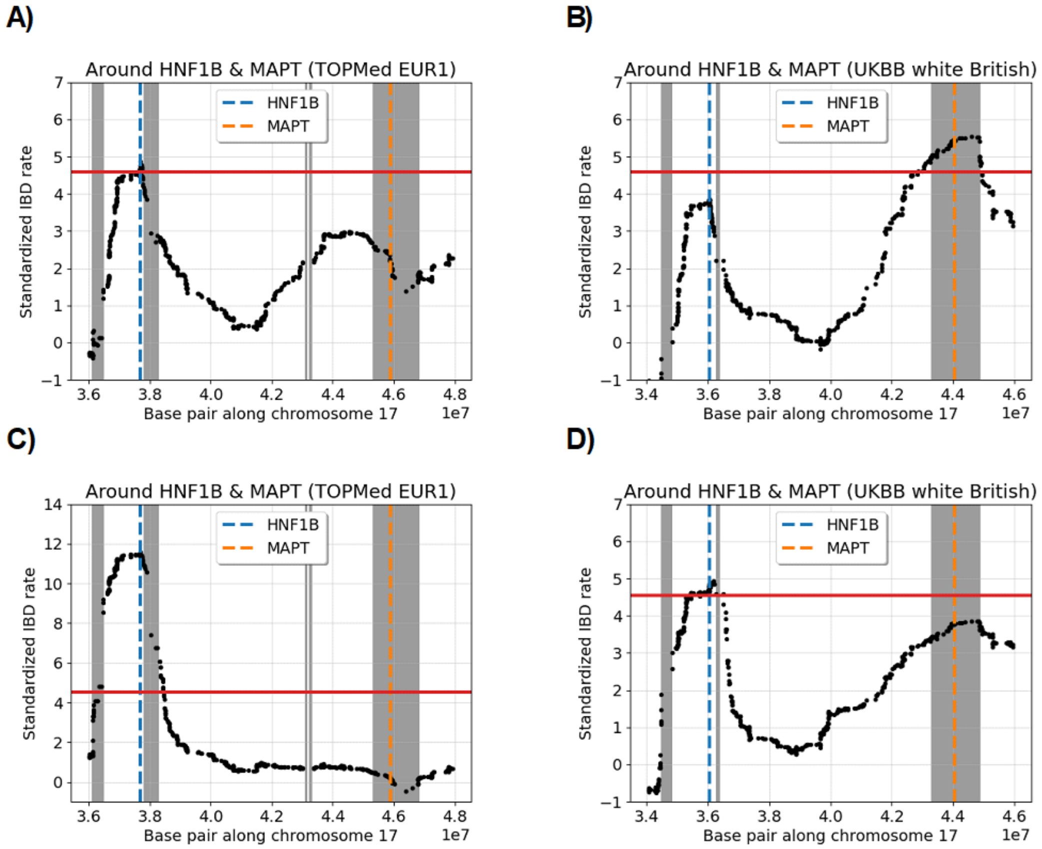

Figure S11: IBD rates around *HNF1B* and *MAPT* in European ancestry samples. Scatter plots show the standardized IBD rates by physical base pair (chromosome 17) for A,C) TOPMed EUR1, and B,D) UKBB white British samples. The IBD segment detection thresholds are A-B) 2.0 cM and C-D) 3.0 cM. The horizontal red lines mark the genome-wide significance thresholds. The vertical blue and orange dashed lines mark the approximate midpoints of *HNF1B* and *MAPT*. The y-axis scales may differ to accentuate the dataset-specific signal. The physical base pairs are with respect to different assemblies. Problematic regions are shaded in gray.

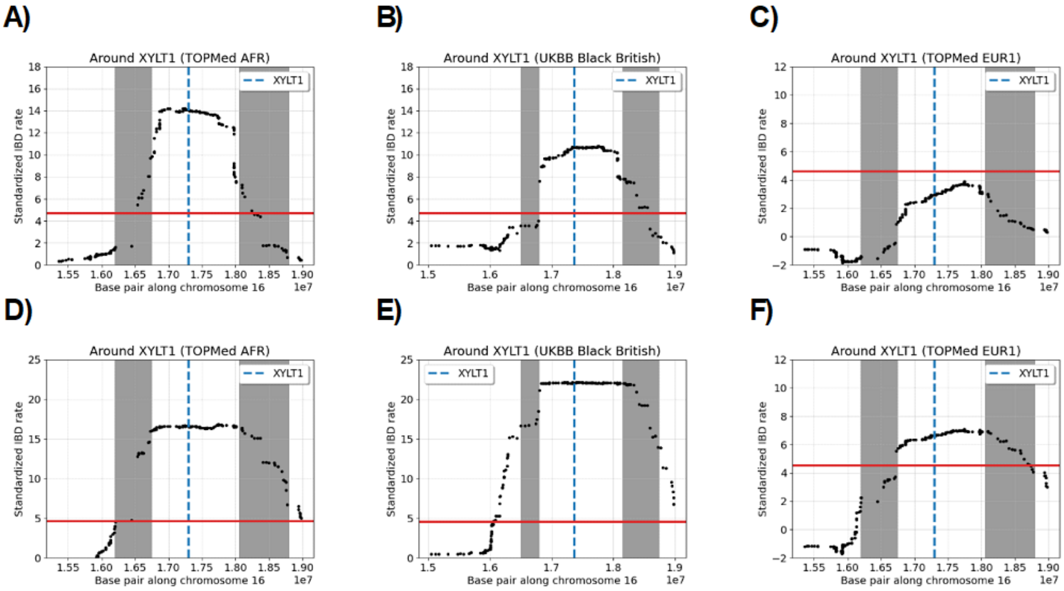

Figure S12: IBD rates around *XYLT1* in African and European ancestry samples. Scatter plots show the standardized IBD rates by physical base pair (chromosome 16) for A,D) TOPMed AFR, B,E) UKBB Black British, and C,F) TOPMed EUR1 samples. The IBD segment detection thresholds are A-C) 2.0 cM and D-F) 3.0 cM. The horizontal red lines mark the genome-wide significance thresholds. The vertical blue dashed lines mark the approximate midpoint of *XYLT1*. The y-axis scales may differ to accentuate the dataset-specific signal. The physical base pairs are with respect to different assemblies. Problematic regions are shaded in gray.

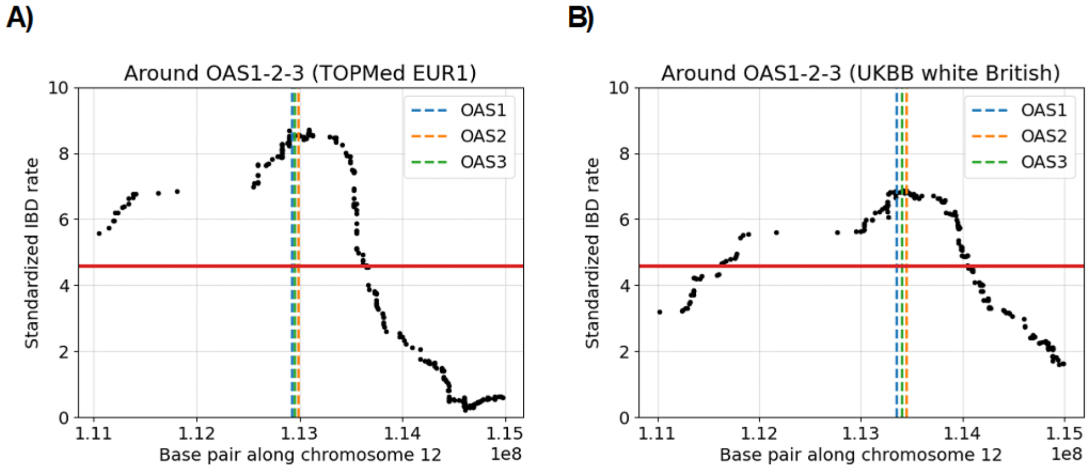

Figure S13: IBD rates around *OAS1-2-3* in European ancestry samples. Scatter plots show the standardized IBD rates by physical base pair (chromosome 12) for A) TOPMed EUR1 and B) UKBB white British samples. The IBD segment detection thresholds is 2.0 cM. The horizontal red lines mark the genome-wide significance thresholds. The vertical blue, orange, and green dashed lines mark the approximate midpoints of *OAS1-2-3*. The physical base pairs are with respect to different assemblies. There are no large problematic regions around this locus.

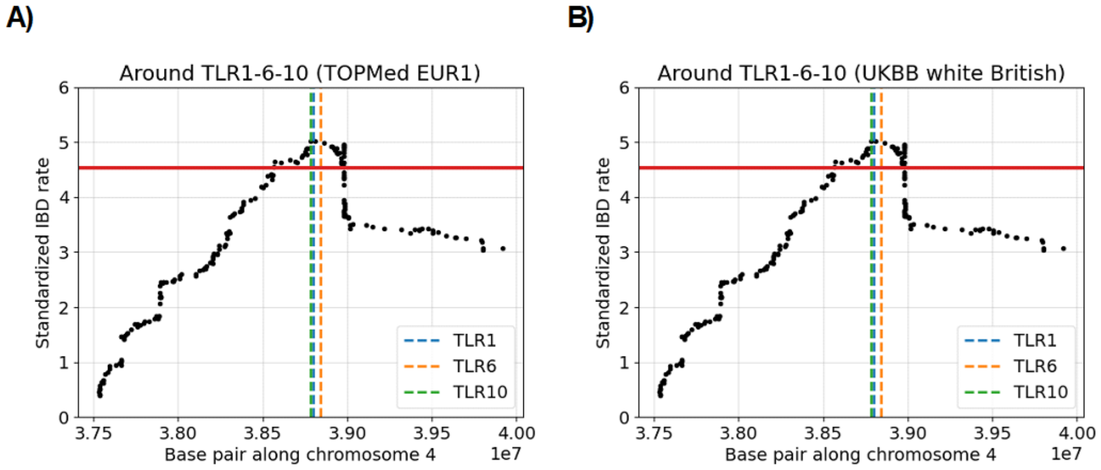

Figure S14: IBD rates around toll-like receptor genes in European ancestry samples. Scatter plots show the standardized IBD rates by physical base pair (chromosome 4) for A) TOPMed EUR1 and B) UKBB white British samples. The IBD segment detection threshold is 2.0 cM. The horizontal red lines mark the genome-wide significance thresholds. The vertical blue, orange, and green dashed lines mark the approximate midpoints of *TLR1-6-10*. The physical base pairs are with respect to different assemblies. There are no large problematic regions around this locus.

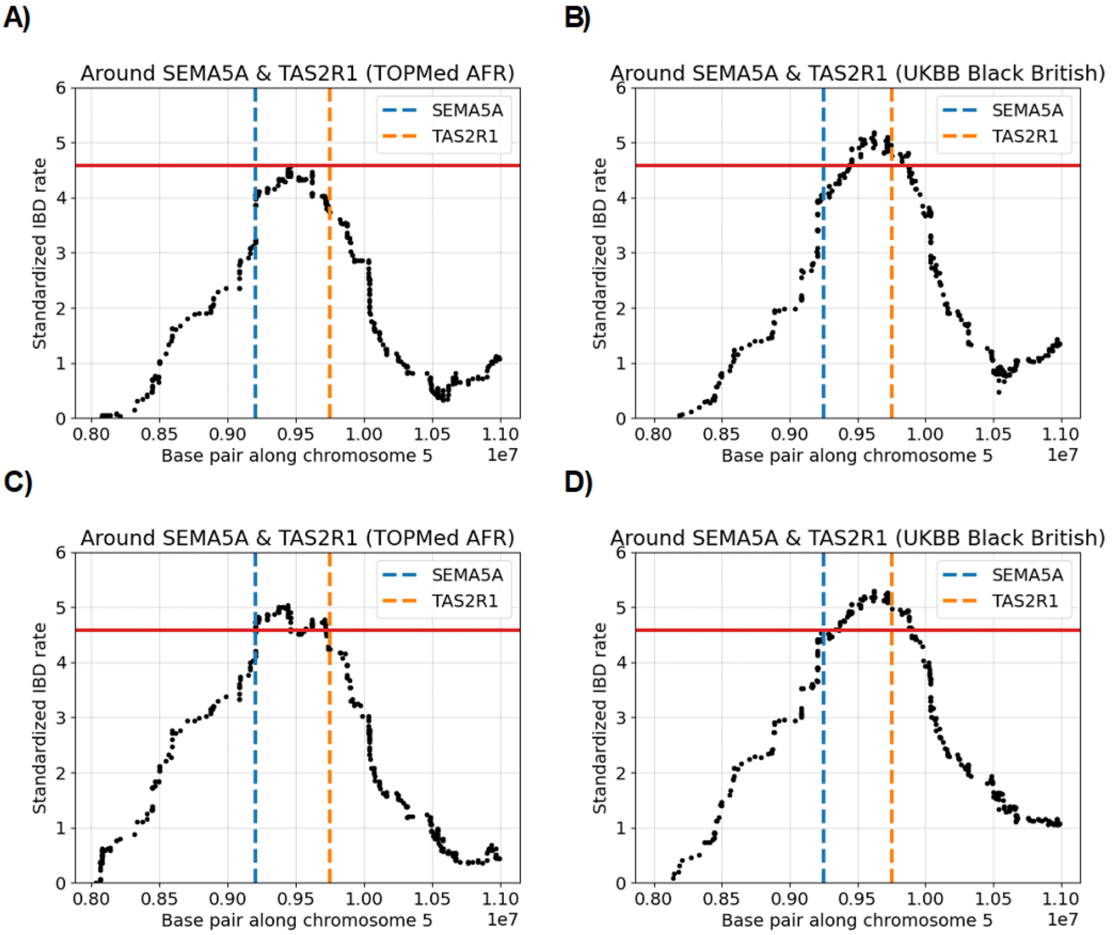

Figure S15: IBD rates around *SEMA5A* and *TAS2R1* in African ancestry samples. Scatter plots show the standardized IBD rates by physical base pair (chromosome 5) for A,C) TOPMed AFR, and B,D) UKBB Black British samples. The IBD segment detection thresholds are A-B) 2.0 cM and C-D) 3.0 cM. The horizontal red lines mark the genome-wide significance thresholds. The vertical blue and orange dashed lines mark the approximate midpoints of *SEMA5A* and *TAS2R1*. The physical base pairs are with respect to different assemblies. There are no large problematic regions around this locus.

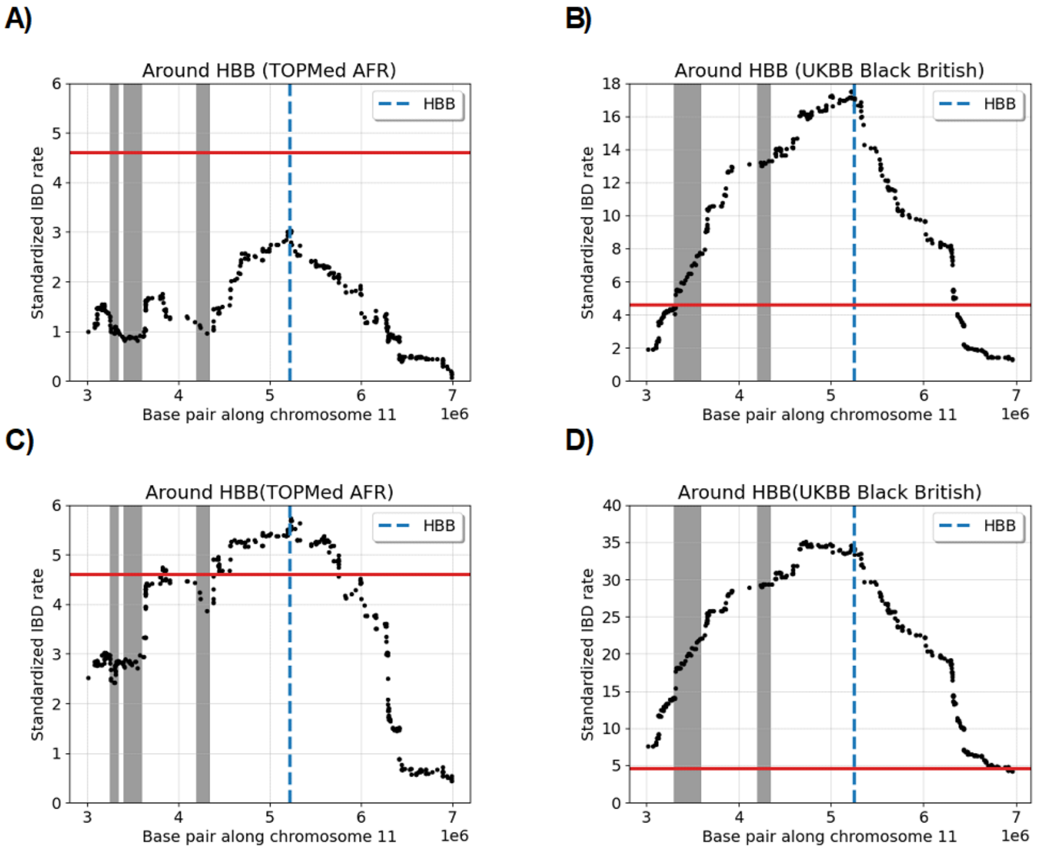

Figure S16: IBD rates around hemoglobin beta genes of African ancestry samples. Scatter plots show the standardized IBD rates by physical base pair (chromosome 11) for A,C) TOPMed AFR and B,D) UKBB Black British samples. The IBD segment detection thresholds are A-B) 2.0 cM and C-D) 3.0 cM. The horizontal red lines mark the genome-wide significance thresholds. The vertical blue dashed lines mark the approximate midpoint of *HBB*. The y-axis scales may differ to accentuate the dataset-specific signal. The physical base pairs are with respect to different assemblies. Problematic regions are shaded in gray.

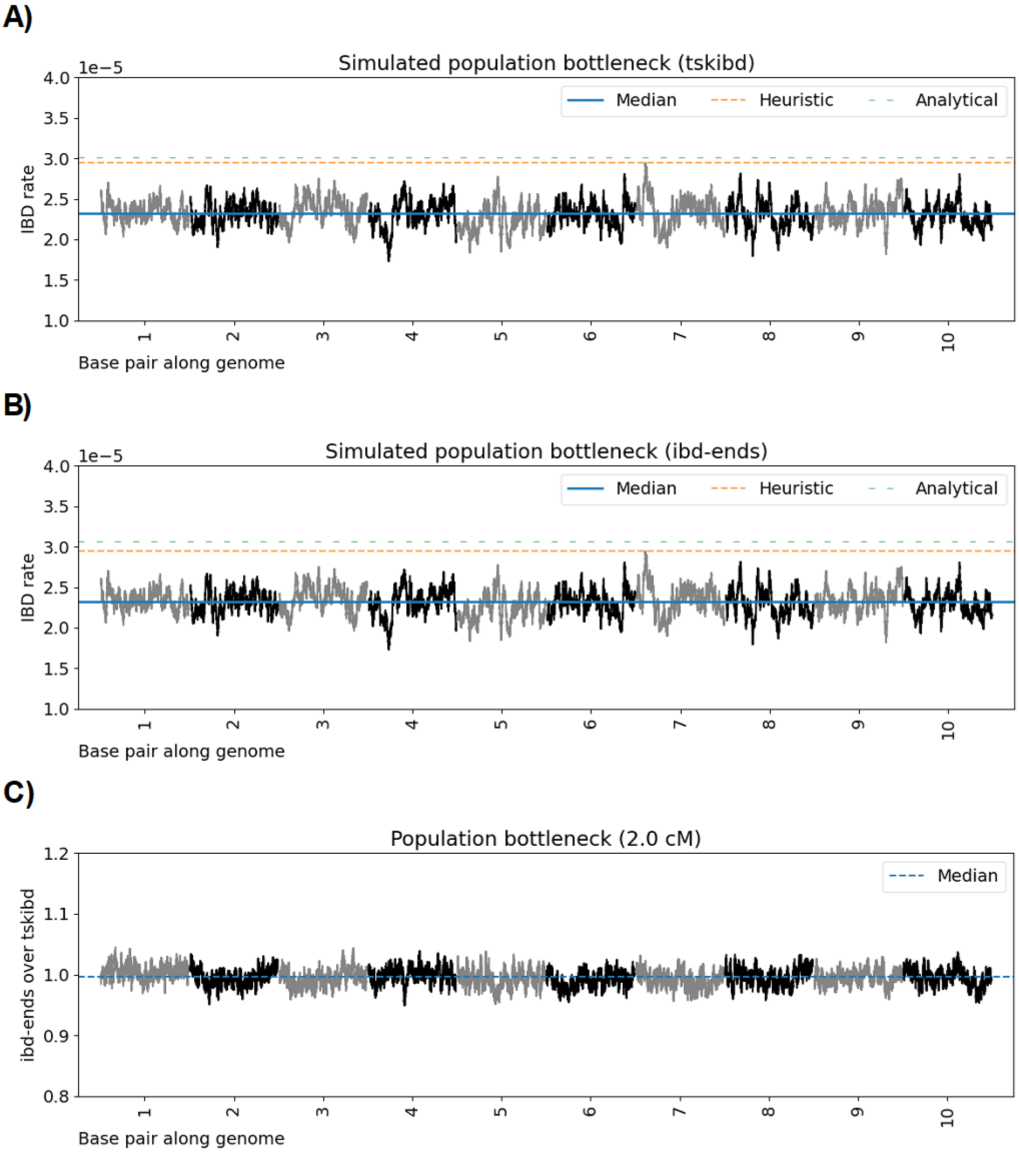

Figure S17: Genome-wide IBD rate scan in a simulated population bottleneck scenario. Line plots show $\geq$ 2.0 cM IBD rates (y-axis) for cM positions along ten simulated chromosomes. Scans are based on A) `tskibd` true IBD segments [6] or B) `ibd-ends` inferred IBD segments [24]. In C), we divide the IBD rates in B) from those in A). Each chromosome is 100 cM. The IBD rate is calculated every 0.02 cM. Data is based on twenty-five hundred diploid samples from the simulated population bottleneck demographic scenario. Horizontal dashed lines show (blue) the genome-wide median IBD rate, (orange) the heuristic threshold of four standard deviations above the median, and (green) the discrete-spacing analytical threshold). The family-wise significance level is 0.05.

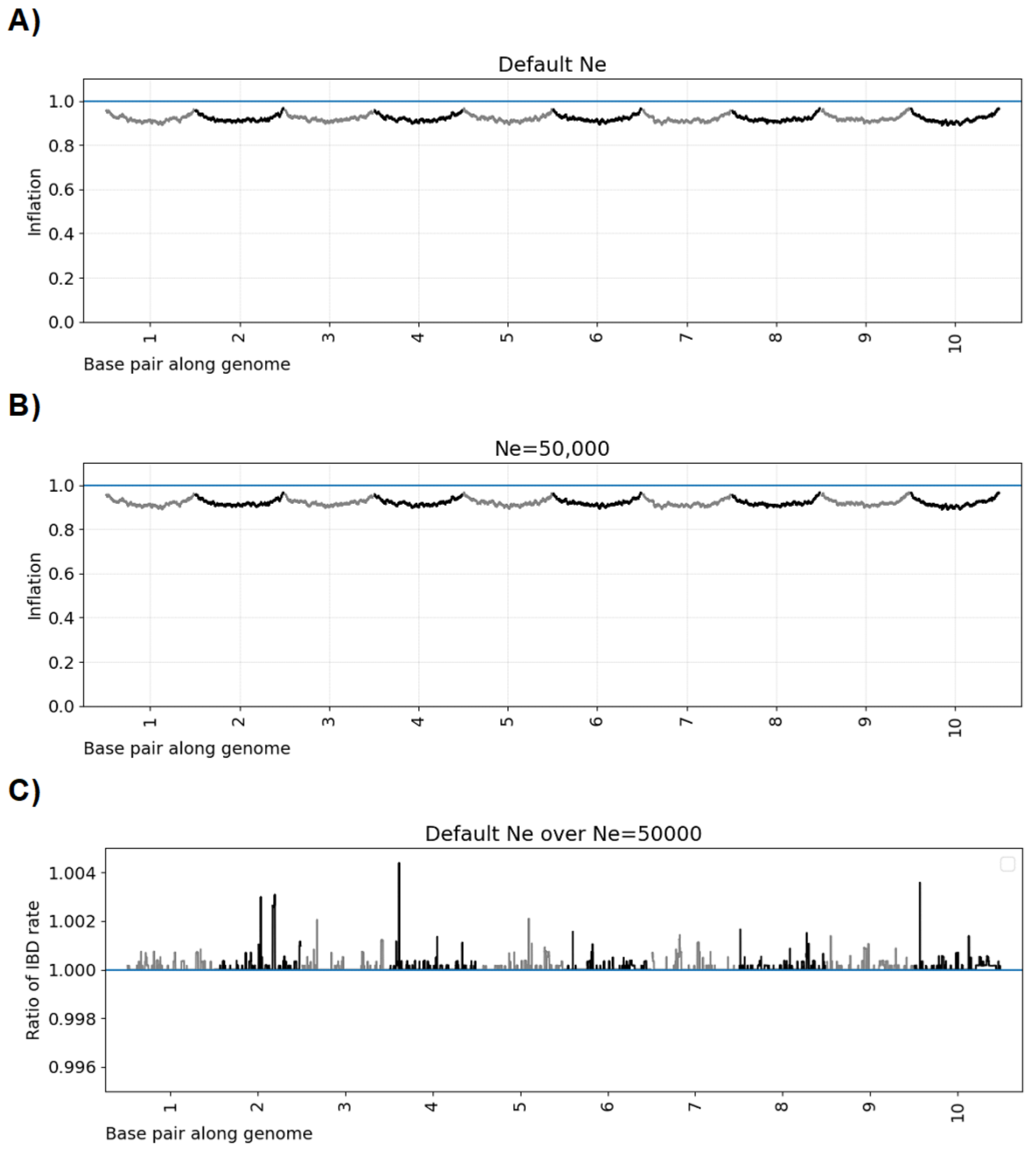

Figure S18: Genome-wide IBD rate scan in a simulated constant population size scenario. Line plots show inferred IBD rates over true IBD rates (y-axis) for cM positions along ten simulated chromosomes. Scans are based on using `ibd-ends`'s A) default `ne` setting versus B) `ne=50000`. In C), we divide the inferred IBD rates in A) and B). Each chromosome is 100 cM. The IBD rate is calculated every 0.02 cM. Data is based on twenty-five hundred diploid samples from the simulated scenario of a constant population of fifty thousand individuals. The IBD segment detection threshold is ≥ 2.0 cM.

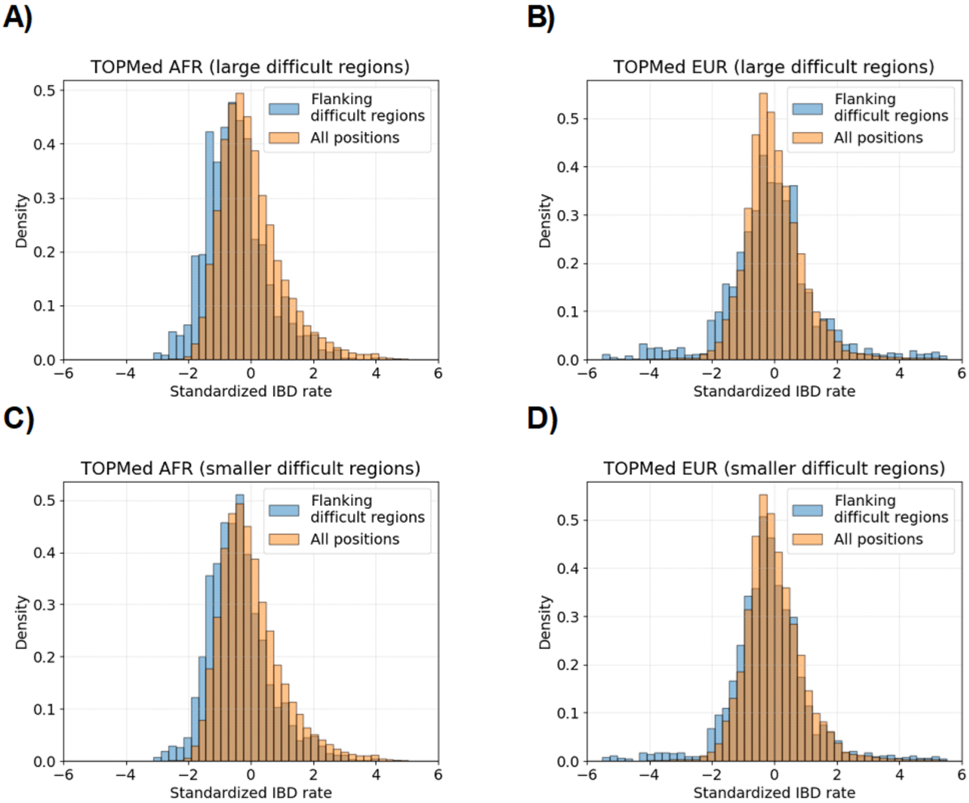

Figure S19: IBD rates flanking low mappability regions. Histograms show standardized IBD rates genome-wide (orange) and near low mappability regions (blue) in the TOPMed AFR A,C) and TOPMed EUR1 B,D) groups. We considered big low mappability regions from the GIAB problematic regions if they spanned A,B) more than 100 kb (referred to “large”) or C,D) 50 kb (referred to as “small”). We concatenated “large” and “smaller” regions if they were within 50 and 25 kb of each other, respectively. By flanking, we mean that the IBD rates are up to 1 Mb directly to the left and right of a GIAB problematic region.

Supplemental tables

Family-wise level	Adjusted Analytical	Simulation	Bonferroni	FWER Analytical	Simulation
0.01	1.58e-6	2.01e-6	2.08e-7	0.008	0.012
0.05	9.23e-6	1.06e-5	1.04e-6	0.030	0.034
0.10	2.03e-5	2.29e-5	2.08e-6	0.066	0.078

Table S1: Significance levels and family-wise error rates after multiple-testing corrections with IBD segments $\geq$ 3.0 cM. Significance levels are adjusted for multiple testing based on scans over 10 chromosomes of size 100 cM and tests every 0.02 cM (50,000 total tests). The multiple-testing analytical and simulation-based thresholds are based on a fitted Ornstein-Uhlenbeck process. Each simulation has a different threshold as a result of estimating θ. Family-wise error rate (FWER) is the percentage of five hundred genome-wide scans with at least one statistically significant result. The demographic scenario is the population bottleneck.

Dataset	Chr	Rate (1e-4)	Region size (cM)	Position (Mb)	Genes	p value
TOPMed EUR1 (GRCh38)	2	1.37	7.94	134.84 (132.29-140.09)	*LCT*†	2.59e-187
	6	1.23	8.04	31.03 (23.91-36.38)	*MHC**†	2.14e-143
	17	0.71	3.44	37.68 (36.33-38.44)	*HNF1B**†	2.59e-31
	16	0.54	3.00	17.74 (16.73-18.72)	*XYLT1**	8.50e-13
	22	0.52	5.12	20.23 (19.15-21.10)	.	6.37e-11
	12	0.50	5.10	51.38 (48.80-53.20)	*KRT*†	1.74e-9
	15	0.48	2.28	31.30 (30.46-32.15)	*TRPM1**†	4.19e-8
UKBB white British 410k (GRCh37)	2	3.98	8.72	135.91 (132.86-141.34)	*LCT*†	$< 5e\text{-}324$
	6	1.65	7.92	30.80 (23.91-36.34)	*MHC**†	2.63e-74
	15	1.42	3.50	30.94 (30.16-32.92)	*TRPM1**†	1.26e-47
	16	1.02	3.28	18.25 (16.37-18.95)	*XYLT1**	6.40e-16
	22	0.91	3.46	21.53 (20.98-21.61)	.	1.91e-10
	12	0.91	5.12	51.78 (49.38-53.76)	*KRT*†	2.04e-10
	17	0.83	1.26	36.18 (35.44-36.49)	*HNF1B**†	4.16e-7
UKBB Indian British (GRCh37)	2	1.41	5.12	136.97 (134.36-139.51)	*LCT*†	1.69e-36
	19	1.12	4.92	50.23 (48.47-50.74)	.	5.25e-16
	6	1.10	3.12	33.96 (33.02-36.34)	*MHC**†	4.70e-14
	16	0.96	2.80	18.06 (16.83-18.28)	*XYLT1**	1.65e-7
TOPMed AFR (GRCh38)	16	1.39	3.44	17.73 (16.45-19.09)	*XYLT1**	1.31e-63
	22	0.92	5.56	20.26 (18.95-21.10)	.	3.30e-21
	19	0.69	1.98	1.78 (1.72-2.10)	.	3.56e-9
	11	0.68	2.74	5.23 (3.83-5.75)	*HBB*†	4.83e-9
	5	0.37	1.56	9.44 (9.20-9.72)	*TAS2R1*	2.34e-7
UKBB Black British (GRCh37)	11	3.79	7.78	4.72 (2.76-6.92)	*HBB*†	2.78e-271
	16	2.52	4.14	17.40 (16.06-19.14)	*XYLT1**	4.12e-109
	22	1.28	4.74	21.54 (19.64-22.33)	.	1.68e-21
	5	0.88	1.64	9.62 (9.34-9.90)	*TAS2R1*	5.77e-8
	20	0.86	1.54	40.93 (39.47-40.99)	.	1.30e-7
	7	0.83	0.50	80.35 (80.08-80.40)	*SEMA3C*	7.61e-7

Table S2: Loci detected in the ≥ 3.0 cM selection scans. We report loci where identity-by-descent (IBD) rates exceed the discrete-spacing analytical thresholds of 0.45e-4, 0.81e-4, 0.93e-4, 0.61e-4, and 0.81e-4 for the TOPMed EUR1 ancestry, UKBB white British, UKBB Indian British, TOPMed AFR ancestry, and UKBB Black British sample sets, respectively. The maximum IBD rate is given for each locus. Physical positions for the location of the maximum IBD rate and the span of excess IBD rates are shown in megabases (Mb). We report the size in centiMorgan (cM) of each region, which is defined to be a contiguous stretch of IBD rates exceeding the genome-wide significance threshold. Pedigree-based recombination maps from Halldorsson et al. [83] and Bhérer et al. [85] aligned to the GRCh38 and GRCh37 reference genomes are used for inferring IBD segments in the TOPMed and UKBB sample sets, respectively. p values are calculated assuming the null model that IBD rates are normally distributed. Annotated genes or gene complexes are discussed in the main text. The signals with asterisks overlap hotspots of recurrent copy number variation and/or have multiple alternate locus sequences that have been added by the Genome Reference Consortium. The signals with daggers have previously been reported in the literature. The signals with dots are not discussed with respect to specific genes or gene complexes.

Dataset	Genes	Count of outlier clusters	Proportion of largest cluster	Proportion of second largest cluster	Proportion of all clusters
TOPMed AFR	*XYLT1*	4	2.1	1.4	5.1
UKBB Black British	*XYLT1*	6	2.2	1.2	6.5
	HBB	3	8.6	1.6	11.5
	SEMA3C	3	5.7	1.0	7.7
UKBB Indian British	*LCT*	2	15.2	1.9	17.1
	OCA2	12	1.6	0.6	5.2
	MHC	12	3.4	2.4	13.2
	XYLT1	15	0.6	0.5	4.8
TOPMed EUR	*LCT*	1	65.1	.	65.1
	*OCA2**	1	68.5	.	68.5
	OAS	8	9.4	3.1	19.3
	MHC	7	12.4	8.8	29.5

Table S3: Fine mapping candidate alleles, or lack thereof, for positive selection. We applied the Temple et al. [14] suite of methods to detect unusually large clusters sharing IBD haplotypes. We used the default parameter settings. The sample proportions in the IBD clusters are with respect to the entire sample. We only investigated some loci to compare the signal at *XYLT1* against known positively selected loci *LCT* and *OCA2* and the complex *MHC* region under balancing selection. The *OCA2* result (asterisk) is from the Temple et al. [14] analysis of the Women's Health Initiative data in TOPMed. A more exhaustive analysis of the TOPMed EUR1 results is in Temple et al. [14]. For *LCT* and *OCA2*, there is broad consensus in the literature supporting the selective sweep hypothesis. The dot denotes that there there is not a second excess IBD sharing cluster.

Supplemental acknowledgements

We gratefully acknowledge the individual studies and participants who provided biological samples and data for the TOPMed project. Funding for the Barbados Asthma Genetics Study (BAGS) was provided by the National Institutes of Health (NIH) R01HL104608, R01HL087699, and HL104608 S1. The Mount Sinai BioMe Biobank (BioMe) has been supported by The Andrea and Charles Bronfman Philanthropies and in part by funds from the NHLBI and the National Human Genome Research Institute (NHGRI) (U01HG00638001; U01HG007417; X01HL134588); genome sequencing was funded by contract HHSN268201600037I. The Cleveland Clinic Atrial Fibrillation study (CCAF) was supported by NIH grants R01 HL 090620 and R01 HL 111314, the NIH National Center for Research Resources for Case Western Reserve University and Cleveland Clinic Clinical and Translational Science Award UL1-RR024989, the Cleveland Clinic Department of Cardiovascular Medicine philanthropy research funds, and the Tomsich Atrial Fibrillation Research Fund; genome sequencing was supported by R01HL092577. The Framingham Heart Study (FHS) was supported by contracts NO1-HC-25195, HHSN268201500001I, and 75N92019D00031 from the NHLBI and grant supplement R01 HL092577-06S1; genome sequencing was funded by HHSN268201600034I and U54HG003067. The Hypertension Genetic Epidemiology Network Study (HyperGen) is part of the NHLBI Family Blood Pressure Program; collection of the data represented here was supported by grants U01 HL054472, U01 HL054473, U01 HL054495, and U01 HL054509; genome sequencing was funded by R01HL055673. The Jackson Heart Study is supported and conducted in collaboration with Jackson State University (HHSN268201300049C and HHSN268201300050C), Touga-

loo College (HHSN268201300048C), and the University of Mississippi Medical Center (HHSN268201300046C and HHSN268201300047C) contracts from NHLBI and the National Institute for Minority Health and Health Disparities (NIMHD); genome sequencing was funded by HHSN268201100037C. The My Life, Our Future samples (MLOF) and data are made possible through the partnership of Bloodworks Northwest, the American Thrombosis and Hemostasis Network, the National Hemophilia Foundation, and Bioverativ; genome sequencing was funded by HHSN268201600033I and HHSN268201500016C. The Venous Thromboembolism project (VTE) was funded in part by grants from the NIH, NHLBI (HL66216 and HL83141), and the NHGRI (HG04735). The Vanderbilt Genetic Basis of Atrial Fibrillation study (VUAF) was supported by grants from the American Heart Association (EIA 0940116N) and grants from the National Institutes of Health (HL092217, U19 HL65962, and UL1 RR024975), and by CTSA award (UL1TR000445) from the National Center for Advancing Translational Sciences; genome sequencing was funded by R01HL092577. The Women's Health Initiative program (WHI) is funded by NHLBI through contracts 75N92021D00001, 75N92021D00002, 75N92021D00003, 75N92021D00004, 75N92021D00005; genome sequencing was funded by HHSN268201500014C.
